# Optimal Therapies for Recurrent Glioblastoma: A Bayesian Network Meta-Analysis

**DOI:** 10.3389/fonc.2021.641878

**Published:** 2021-03-29

**Authors:** Wenlin Chen, Yuekun Wang, Binghao Zhao, Penghao Liu, Lei Liu, Yu Wang, Wenbin Ma

**Affiliations:** Department of Neurosurgery, Peking Union Medical College Hospital, Chinese Academy of Medical Sciences and Peking Union Medical College, Beijing, China

**Keywords:** bevacizumab, systematic review, combination therapy, recurrent glioblastoma, Bayesian network meta-analysis

## Abstract

The optimal treatment of recurrent glioblastoma (GBM) remains controversial. Therefore, our study aimed to compare and rank active therapies in recurrent GBM. We performed a systematic review and a Bayesian network meta-analysis. We obtained a treatment hierarchy using the surface under the cumulative ranking curve and mean ranks. A cluster analysis was conducted to aggregate the separated results of three outcomes. The protocol was registered in PROSPERO (CRD42019146794). A total of 1,667 citations were identified, and 15 eligible articles with 17 treatments remained in the final network meta-analysis. Pairwise comparison showed no significant difference on the 6-month progression-free survival (6-m PFS) rate, objective response rate (ORR), and overall survival (OS). Among the reports, cediranib plus lomustine (CCNU) corresponded to the highest rates of grade 3-4 adverse events. Ranking and cluster analysis indicated that bevacizumab (BEV) plus CCNU and regorafenib had a higher efficacy on the ORR, 6-m PFS rate and OS, and that BEV monotherapy or BEV combined with active drug therapies was advantageous for the ORR and 6-m PFS rate. Additionally, tumor treatment fields (TTF) plus BEV showed a relatively higher SUCRA value in OS. According to ranking and cluster analysis, BEV plus CCNU and regorafenib are the primary recommendations for treatment. BEV monotherapy alone or combined with active drug therapies are recommended in patients with severe neurological symptoms. Advanced therapy, such as TTF and immunotherapy, remain to be investigated in future studies.

## Introduction

Glioblastoma (GBM) is a primary central nervous system (CNS) tumor associated with poor prognosis and significant invasiveness (1). The prognosis of GBM remains poor despite first-line therapy, and the median overall survival (OS) is 12-15 months (2), while the 5-year survival does not exceed 5% (3). Tumor recurrence is the leading cause of death, and the rate of recurrence is rather high after initial treatment (4). Most patients (90%) experience recurrence in situ, and no significant relationship has been observed with the choice of therapy. Therefore, GBM recurrence has become a clinically significant issue.

Currently, participating in ongoing clinical trials prior to standard chemotherapy is strongly recommended for patients with recurrent GBM according to the National Comprehensive Cancer Network (NCCN) guideline. Other preferred treatments include bevacizumab (BEV), temozolomide (TMZ), lomustine (CCNU) or carmustine, PCV and regorafenib. The combination therapy of carmustine or CCNU + BEV and TMZ + BEV is also recommended when BEV monotherapy loses efficiency. BEV is considered to be ineffective in OS prolongation according to the European Association for Neuro-Oncology (EANO) guideline (5). Several clinical trials are currently in progress for improving the prognosis of recurrent GBM. The main exploratory therapies are chemotherapy, targeted therapy, vaccines, and tumor treatment fields (TTF). However, the recommendation categories of the second-line treatments in the NCCN guidelines are 2A, 2B, and 3, and further evidence is urgently needed to determine the feasibility of the relevant therapies.

Network meta-analysis (NMA) is a statistical approach that provides both direct and indirect evidence, which anchors the arms of the treatments in different clinical trials (6). NMA can summarize the results of several different randomized controlled trials (RCTs) by forming an evidence chain and combining studies for analysis in a network. It is also able to rank the treatment approaches and present the statistical results in a graphical format (7). NMA has already been used successfully in several fields of medicine (8).

This NMA study established a clinically meaningful hierarchy of efficacy and safety of different therapies for adult patients with recurrent GBM from published RCTs. Our study is the first to systematically integrate and compare the results of clinical trials on treating adult patients with recurrent GBM and aims to provide potential guidance for therapies for recurrent GBM.

## Materials and Methods

### Search Strategy and Selection Criteria

We performed a systematic review, and published RCTs were searched with assistance of a librarian in the following electronic databases: EMBASE, PubMed, MEDLINE and MEDLINE In-Process. We also manually searched for published, unpublished, and ongoing clinical trials in the WHO International Clinical Trials Registry Platform (ICTRP), the EU Clinical Trials Register, the ClinicalTrials.gov results database, the U.S. Food and Drug Administration (FDA) database, and other clinical trial registration databases and drug-approval agencies. We used the following search terms: “((brain OR CNS OR central nervous system OR cranial OR intra* cranial) AND (tumor OR neoplasm* OR cancer OR malignan*) OR (glioma* OR LGG OR HGG OR astrocyt* OR astroglioma OR glioblastoma* OR oligodendrogli* OR ependym* OR oligoastrocytoma* OR astroblastoma* OR ganglioglioma* OR gliosarcoma* OR glial*) AND (recurren* OR recur OR relaps* OR recidivat* OR reappear* OR recrudesc* OR secondary OR progressive))” and included all results from the inception of the databases through May 2020.

We included RCTs of chemotherapy, targeted therapy and immunotherapy in recurrent GBM multiforme involving placebo-controlled, head-to-head, and multiarmed clinical trials. Adult patients (age>18 years) with a histopathological diagnosis of glioma recurrence were included. Single-armed trials, phase I RCTs, repeated reports, and pilot studies were excluded, and *post hoc* analyses were specifically determined. Additionally, quasi-randomized control trials, pseudo-randomized control trials or RCTs with obvious high bias were excluded.

Study selection, data extraction, and risk assessment were completed independently by two reviewers. Any discrepancies were resolved by consensus and arbitration by another group of reviewers. The protocol of this network meta-analysis was registered in PROSPERO (registration number CRD42019146794).

### Outcomes

We set up three outcomes, which included the 6-month progression-free survival (6-m PFS) rate, overall survival (OS), and objective response rate (ORR). The starting point for survival calculations was defined by each clinical trial and mainly constituted the time of randomization or treatment initiation. The 6-m PFS rate was defined as the percentage of patients who remained alive and progression-free at 24 weeks since the starting point. OS was defined as the survival time from the starting point until death. ORR was defined as a complete or partial response observed on two consecutive MRIs obtained four or more weeks apart.

### Data Analysis

A structured data extraction form was developed by a consensus of all the reviewers based on the Cochrane Handbook for Systematic Reviews of Interventions guidelines and Arm-level data were extracted, and missing outcome data were estimated following the methods reported by Tierney et al. (9).

We presented the characteristics of all included studies by generating a network diagram, and descriptive statistics were utilized to show the clinical and methodological features of the included studies. We estimated the summary relative risk (RR) for dichotomous outcomes and the hazard risk (HR) for OS using a Bayesian network meta-analysis (10). The binomial likelihood was used for all outcomes, and the study effect sizes were then synthesized using a random-effects network meta-analysis model. We obtained a treatment hierarchy using the surface under the cumulative ranking curve (SUCRA) and mean ranks. A ranking of all treatments based on efficacy can be determined by the SUCRA value and presented simply as a single number (7). Sensitivity analysis was done to exclude trials including patients with specific genomic alterations. We assessed the goodness-of-fit of the model to the data by calculating the residual deviance. Heterogeneity was evaluated by comparing residual deviance between consistent and inconsistent models. The risk of bias of each included study was assessed under the guidance of the tool described in the Cochrane Collaboration Handbook and the certainty of evidence was evaluated followed the guidelines of Grading of Recommendations Assessment, Development and Evaluation (GRADE) framework (11). A cluster analysis and principal component analysis (PCA) were conducted to aggregate the separated results of comparisons for three outcomes.

Our models were fitted in WinBUGS (MRC Biostatistics Unit, Cambridge, and Imperial College School of Medicine, London, UK. version 1.4.3) and network graphs, result figures, and cluster analysis were performed using R 3.6.3 and STATA (version 15.1; StataCorp LLC, College Station, TX, USA). A rank-heat plot was used to present SUCRA results following the methodology by Veroniki (12). The study funders had no role in the study design, data collection, data analysis, and interpretation or writing of the report. This study was approved by the Institutional Review Board of Peking Union Medical College Hospital, in accordance with the Declaration of Helsinki.

## Results

**Figure 1:**
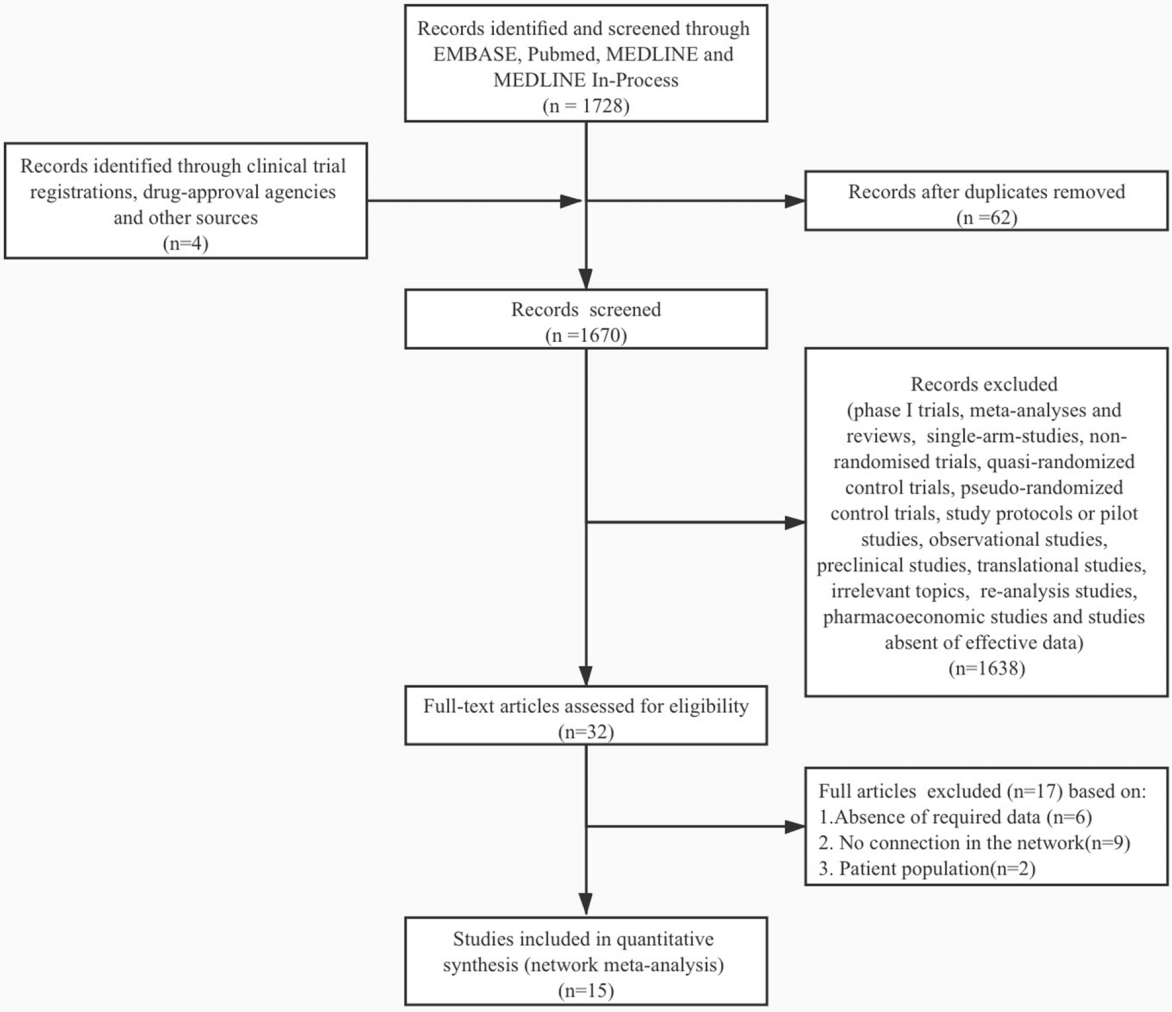
Study selection flowchart. A total of 1667 citations were identified by the primary research, and 15 eligible articles remained in the final network meta-analysis. The flowchart was made under the PRISMA guideline (13).

**Figure 2:**
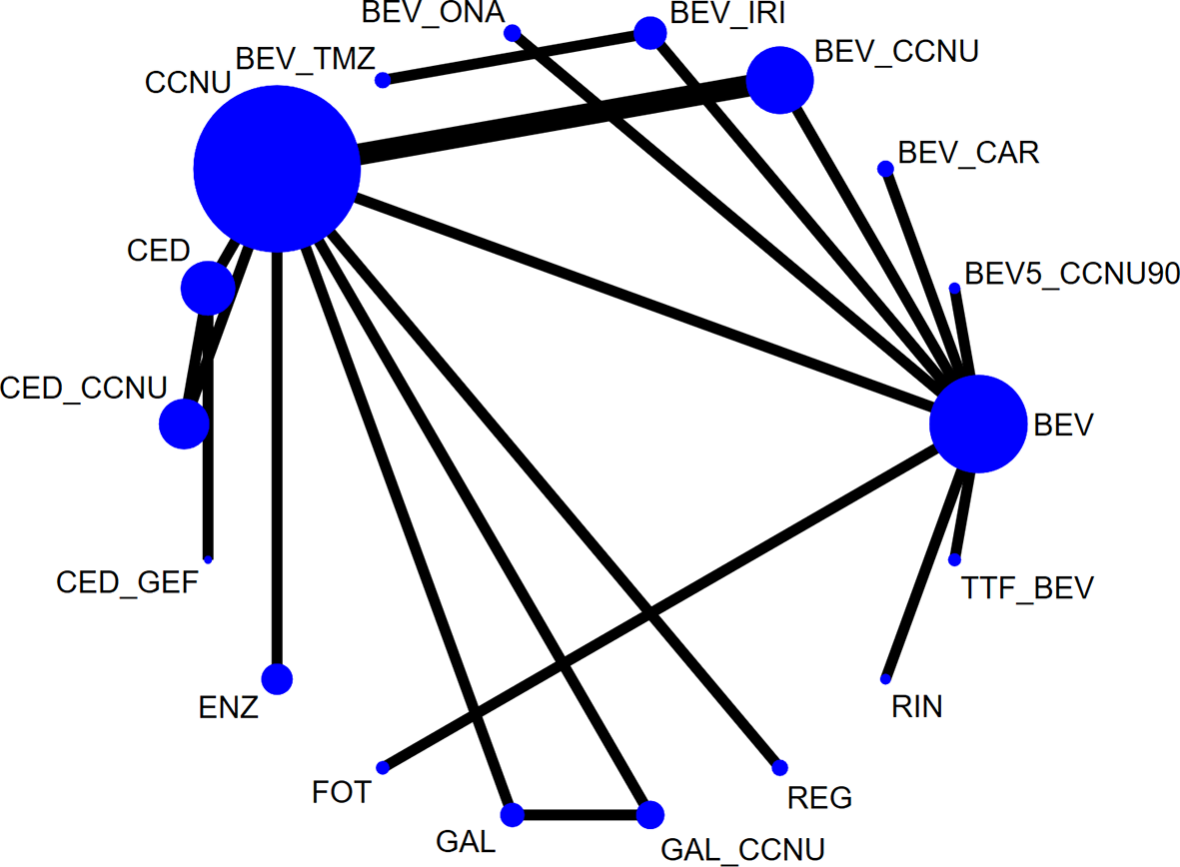
Network plot of all eligible comparisons involved. The size of every solid circle is proportional to the total sample size, and the width of the line is proportional to the number of clinical trials. Network plots of each outcome are shown in **Supplementary Figures**. BEV, bevacizumab monotherapy or combined with placebo; CCNU, lomustine monotherapy or plus placebo; TMZ, temozolomide; BEV_CCNU, bevacizumab plus lomustine; BV5_CCNU90, low-dose bevacizumab (5 mg/kg) plus low-dose lomustine (90 mg/m^2^); BEV_TMZ, bevacizumab plus temozolomide; BEV_ONA, bevacizumab plus onartuzumab; BEV_IRV, bevacizumab plus irinotecan; BEV_CAR, bevacizumab plus carboplatin; GAL, galunisertib; FOT, fotemustine; ENZ, enzastaurin; CED, cediranib; GAL_CCNU, galunisertib plus lomustine; CED_CCNU, cediranib plus lomustine; CED_GEF, cediranib + gefitinib; TTF_BEV, tumor treatment field plus bevacizumab; REG, regorafenib; RIN_BEV, rindopepimut plus bevacizumab.

A total of 1,667 citations were identified by the primary research, and 15 eligible articles remained in the final network meta-analysis (**Figures 1**, **S1**, **S5**, **S9**). The baseline characteristics of the involved studies are shown in **Table 1**. A total of 2253 patients were randomly assigned to 18 treatment arms, which included 17 active drug therapies, of which the majority are based on BEV or CCNU, and TTF plus BEV (**Figure 2**). Four phase III (15, 16, 25, 26) and 11 phase II RCTs (14, 17–24, 27, 28) were included with a median age of involved patients ranging from 52 to 61 years. Assessment of model fitting and risk of bias are shown in the supplementary documents. The median heterogeneity variances were estimated as 0.16 (95%CrI, 0–0.35) for ORR, 0.14 (95% CrI, 0–0.33) for 6m-PFS rate and 0.11 (95%CrI, 0–0.27) for OS, which shown a low-to-moderate heterogeneity (**Table S2**). Six of 17 included RCTs shown a high risk of bias, which were all referred to non-blinded design, based on Cochrane Collaboration Handbook (**Table S4**). The qualitative evaluation of evidence was done followed GRADE guideline, and we evaluate most evidences as low-to moderate (**Tables S5**–**7**).

**Table 1:** Baseline characteristics of included studies.

Study	Phase	Treatment Arms	Number of Patients	Median Age	Sex Ratio (M: F)	KPS-above-80 Ratio	GBM Ratio	Surgery History	RT History	TMZ History	Other chemo-therapy Histories	Steroid Usage
14, J Clin Oncol	II	BEV	85	54	2.14	44.7%	91.8%	un	100%	100%	un	50.6%
		BEV+ Irinotecan	82	57	2.28	37.8%	92.7%	un	100%	100%	un	52.4%
15, J Clin Oncol	III	Enzastaurin	174	un	2.00	51.7%	98.8%	100%	100%	un	un	52.9%
		CCNU	92	un	1.56	48.9%	97.9%	98.9%	98.9%	un	un	52.2%
16, J Clin Oncol	III	Cediranib	131	54	un	50.0%	100%	38.20%	100%	un	un	48.9%
		Cediranib + CCNU	129	54	un	51.2%	100%	38%	100%	un	un	55.0%
		CCNU + Placebo	65	54	un	62.5%	100%	36.90%	100%	un	un	40.0%
17, Lancet Oncol	II	BEV	50	58	1.78	90.0%	100%	10%	100%	0%	un	54.0%
		CCNU	46	56	1.30	87.0%	100%	13%	100%	0%	un	48.0%
		BEV + CCNU (90mg/m2)	44	58	2.14	89.0%	100%	11%	100%	0%	un	48.0%
18, Neuro-Oncol	II	BEV + Carboplatin	60	55	1.31	35.0%	100%	38%	100%	0%	un	83.0%
		BEV	62	55	0.88	35.0%	100%	50%	100%	0%	un	74.0%
19, Neuro-Oncol	II	Galunisertib+ CCNU	79	57.5	2.76	un	100%	un	100%	100%	un	un
		Galunisertib	39	56.6	1.17	un	100%	un	100%	100%	un	un
		CCNU + Placebo	40	56.9	1.35	un	100%	un	100%	100%	un	un
20, Neuro-Oncol	II	BEV	59	59	1.95	un	100%	100%	un	un	un	71.0%
		Fotemustine	32	56	2.56	un	100%	100%	un	un	un	62.0%
21, Plos One	II	Cediranib + Placebo	19	61	2.80	un	100%	100%	un	un	un	un
		Cediranib + Gefitinib	19	55	2.17	un	100%	100%	un	un	un	un
22, J Neuro-Oncol	II	BEV	35	52.6	0.46	68.6%	100%	un	100%	100%	un	un
		BEV (5 mg/kg) + CCNU (90mg/m2)	36	52.8	0.50	63.9%	100%	un	100%	100%	un	un
23, J Clin Oncol	II	BEV + Onartuzumab	64	54.38	2.20	43.75%	100%	un	98.4%	un	un	un
		BEV + Placebo	65	54.76	1.50	15.38%	100%	un	97%	un	un	un
24, J Neuro-Oncol	II	BEV + TMZ	60	58	1.31	50.0%	100%	98%	100%	un	un	32.0%
		BEV + Irinotecan	57	55	1.48	46.0%	100%	97%	100%	un	un	30.0%
25, Clinical Trial Evaluation	III	TTF+BEV	79	57	3.00	un	100%	100%	100%	100%	un	un
		BEV	30	58	3.00	un	100%	100%	100%	100%	un	un
26, New Engl J Med	III	BEV+ CCNU	288	57.1	1.53	un	100%	100%	100%	100%	un	50.0%
		CCNU	149	59.8	1.57	un	100%	100%	100%	100%	un	47.7%
27, Lancet Oncol	II	Regorafenib	59	54.8	2.28	un	100%	100%	100%	100%	un	53.0%
		CCNU	60	58.9	2.53	un	100%	100%	100%	100%	un	62.0%
28, Clin Cancer Res	II	Rindopepimut+BEV	36	59	1.12	81%	100%	100%	100%	100%	un	50.0%
		BEV	37	55	1.47	81%	100%	100%	100%	100%	un	51.0%

Un, No reports available; RT, radiation therapy; TMZ, temozolomide; BEV, bevacizumab; CCNU, lomustine; TTF, Tumor Treating Field; KPS, Karnofsky performance score; PCV, procarbazine, lomustine, vincristine; J Clin Oncol, Journal of Clinical Oncology; Lancet Oncol, Lancet Oncology; J Neuro-Oncol, Journal of Neuro-Oncology; New Engl J Med, New England Journal of Medicine; Clin Cancer Res, Clinical Cancer Research.

### Objective Response Rate, 6-m Progression-Free Survival Rate, and Overall Survival

**Figure 3:**
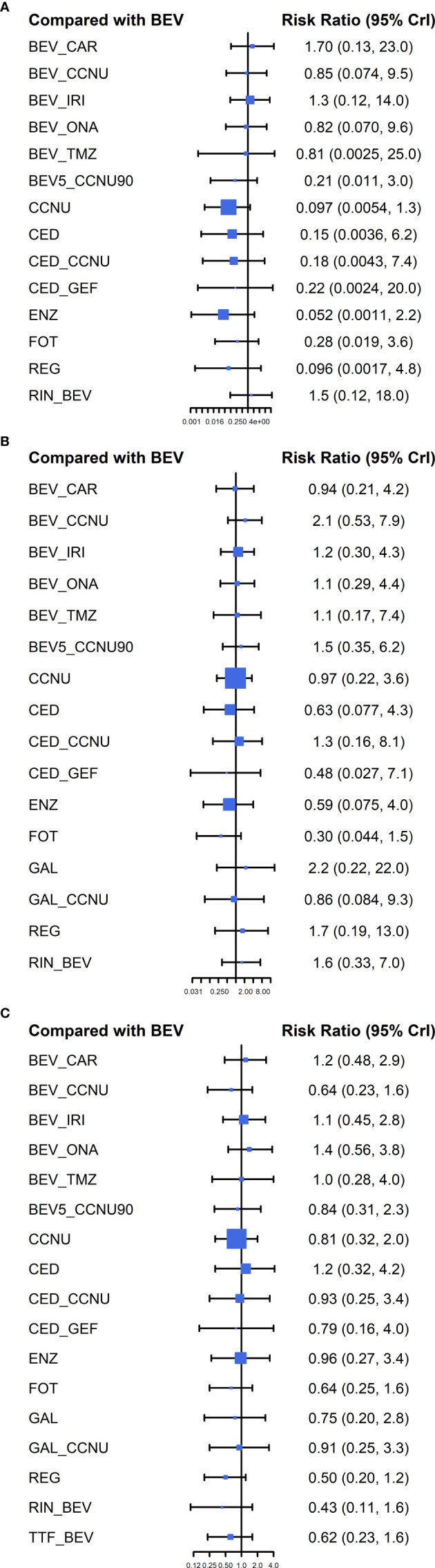
Forest plot of the relative effects compared with BEV. All therapies were compared with BEV in 3 outcomes: **(A)** ORR, **(B)** 6-m PFS rate, and **(C)** OS. The size of every solid square is proportional to the total sample size. The abbreviations are defined in the legend of **Figure 2**.

**Figure 4:**
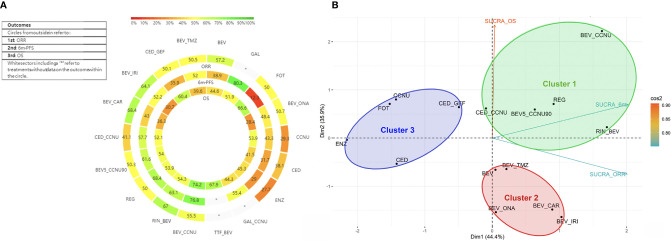
Heat-rank plot and cluster analysis of SUCRA. **(A)** Heat-rank plot of SUCRA. Each circle shows the SUCRA value for ORR, 6-m PFS, and OS from outside to inside. Interactions were labeled, and each sector was colored depending on the SUCRA value. The SUCRA value scale is shown, and “*” refers to missing data. **(B)** Cluster analysis of all 3 outcomes. All therapies involved were divided into three clusters and are shown in 3 circles with distinctive colors. Clusters are shown in a two-dimensional graph by PCA, and the attribution of the SUCRA value of three outcomes to the two dimensions is shown by vectors in different colors. SUCRA_ORR, SUCRA value for ORR; SUCRA_6m, SUCRA value for 6m PFS rate; SUCRA_OS, SUCRA value for OS.

Compared with BEV (**Figure 3**) or CCNU (**Figures S2**, **6**, **10**) monotherapy, other chemotherapy regimens showed no significant positive influence on ORR, 6-m PFS rate and OS. Head-to-head comparisons of all treatments indicated no significant difference among any of the drug therapies involved in our study (**Tables S7**, **9**, **11**). To further reveal the optimal treatment, SUCRA and mean ranks were calculated, which indicated that BEV combined with carboplatin or rindopepimut, followed by BEV plus irinotecan, has higher SUCRA value, while CCNU and enzastaurin were associated with relatively worse outcomes in ORR (**Figures 4A**, **S3** **–** **4**; **Table S8**).

As for 6-m PFS rate, according to SUCRA, BEV plus CCNU and galunisertib tended to be more effective, whereas fotemustine, enzastaurin and cediranib were less frequently recommended (**Figures 4A**, **S7** **–** **8**; **Table S10**). Additionally, regorafenib, rindopepimut plus BEV, and low-dose BEV (5 mg/kg) plus CCNU (90 mg/m^2^) showed slightly less efficacy than a normal dose of BEV plus CCNU combined with other active drugs.

For OS, referring to the results of SUCRA and mean ranks, BEV combined with CCNU was associated with a relatively prolonged OS, followed by TTF plus BEV and fotemustine monotherapy; meanwhile, BEV plus irinotecan or onartuzumab ranked lowest (**Figures 4A**, **S11** **–** **12**; **Table S12**).

### Adverse Effects

Furthermore, 15 RCTs reported categories and frequencies of adverse events for 20 different therapies, rates of specified grade 3 to 4 adverse events, all grade 3 to 4 adverse events and all adverse events. Among the reports, cediranib plus CCNU showed the highest, and fotemustine showed the lowest rates of grade 3-4 adverse events (76.0% vs. 9.4%) (**Table 2**). The highest rate of total adverse events was reported for galunisertib monotherapy, whereas the lowest rate of total adverse events was reported for TMZ monotherapy (94.9% vs. 56.4%). Adverse events were classified according to the National Cancer Institute Common Terminology Criteria for Adverse Events (grade 1, mild; grade 2, moderate; grade 3, severe or medically significant; grade 4, life-threatening).

**Table 2:** Percentage of Patients with Adverse Events According to Treatment.

Treatment	Grade 3-4 Adverse Events (person – times)						All Grade3-4 Adverse Events	All Adverse Events	reference
	Hematologic	Gastro-intestinal	Hemorrhage	Neurologic	Metabolic	Renal			
Bevacizumab+ Lomustine (90mg/m^2^)	156/332 (47.0%)	un	un	un	un	1/44 (2.3%)	180/288 (62.5%)	241/288 (83.7%)	17, 26
Bevacizumab (5mg/kg) +Lomustine (90mg/ m^2^)	24/36 (66.7%)	0	0	0	0	0	un	un	22
Cediranib+ Lomustine	116/129 (89.9%)	un	1/129 (0.8%)	un	un	un	98/129 (76.0%)	un	16
Bevacizumab+ Temozolomide	30/60 (50.0%)	4/60 (6.7%)	4/60 (6.7%)	6/60 (10.0%)	6/60 (10.0%)	0	45/60 (75.0%)	54/60 (90%)	24
Bevacizumab	22/393 (5.6%)	4/343 (1.2%)	2/343 (0.6%)	8/281 (2.8%)	2/343 (0.6%)	6/393 (1.5%)	126/308 (40.9%)	132/144 (91.7%)	14, 17, 18, 20, 22, 23, 25 1
Bevacizumab+ Irinotecan	34/139 (24.5%)	7/139 (5.0%)	4/139 (2.9%)	7/139 (5.0%)	8/57 (14.0%)	1/139 (0.7%)	84/139 (60.4%)	131/139 (94.2%)	14, 24
Bevacizumab+ Carboplatin	13/60 (21.7%)	1/60 (1.7%)	2/60 (3.3%)	un	un	0	37/60 (61.7%)	un	18
Bevacizumab+ Onartuzumab	4/64 (6.3%)	4/64 (6.3%)	0	5/64 (7.8%)	0	0	25/64 (39.1%)	un	23
Lomustine	197/452 (43.6%)	0	2/105 (1.9%)	1/60 (1.7%)	1/60 (1.7%)	0	129/314 (41.1%)	113/189 (59.8%)	15–17, 19 2 1
Fotemustine	14/32 (43.8%)	0	0	0	0	0	3/32 (9.4%)	27/32 (84.4%)	20
Cediranib+ gefitinib	3/29 (10.3%)	1/29 (3.4%)	0	8/29 (27.6%)	2/29 (6.9%)	0	13/29 (44.8%)	19/29 (65.5%)	21
Enzastaurin	1/174 (0.6%)	0	un	un	un	un	un	un	15
Cediranib	9/150 (6.0%)	0	1/150 (0.7%)	2/19 (10.5%)	3/19 (15.8%)	0	94/150 (62.7%)	18/19 (94.7%)	16, 21
Tumor Treating Field+ Bevacizumab	16/144 (11.1%)	5/144 (3.5%)	5/144 (3.5%)	40/144 (27.8%)	4/144 (2.8%)	0	71/144 (49.3%)	un	25
Galunisertib+ Lomustine	23/79 (29.1%)	0	0	0	0	0	20/79 (25.3%)	71/79 (89.9%)	19
Galunisertib	1/79 (1.3%)	0	0	0	0	0	4/39 (10.3%)	37/39 (94.9%)	19
Regorafenib	5/59 (8.5%)	9/59 (15.3%)	0	2/59 (3.4%)	9/59 (15.3%)	0	33/59 (55.9%)	un	17
Rindopepimut+ Bevacizumab	0	0	0	1/36 (2.8%)	2/36 (5.6%)	0	11/36 (30.6%)	un	27

Un, No reports available.

### Optimal Treatment for Recurrent GBM

A higher SUCRA value indicated a better efficacy, which could help determine optimal treatments in recurrent GBM; however, due to the uncertainty, the results must be interpreted carefully (29). As illustrated in **Figure 4B**, except for missing data, BEV plus CCNU, regorafenib and galunisertib showed a relatively prior efficacy in three outcomes.

Cluster analysis was utilized to comprehensively evaluate the efficacy of all therapies and resulted in three distinctive clusters (**Figure 4B**). TTF plus BEV and galunisertib monotherapy alone or combined with CCNU were excluded due to missing data. Cluster 1 involved normal or low-dose BEV plus CCNU, regorafenib, rindopepimut and cediranib plus CCNU. These treatments in the cluster were characterized by higher-to-moderate efficacy on the ORR, 6-m PFS rate and OS. Considering that cediranib plus CCNU showed a higher possibility of having grade 3 to 4 adverse events, BEV plus CCNU and regorafenib were initially recommended. Low-dose BEV plus CCNU had an acceptable adverse effects profile and could be considered for use as an alternative therapy. Cluster 2 (including BEV monotherapy, BEV plus TMZ, irinotecan, onartuzumab or carboplatin) showed advantages in ORR and 6-m PFS rate but not in OS, which proved that BEV combined therapy had an advantage in local control. Cluster 3 (including CCNU, fotemustine, enzastaurin, cediranib monotherapy or cediranib plus gefitinib) showed a moderate-to-high OS efficacy but a lower efficacy on the local control, and these agents were the last to be recommended.

Also, a sensitivity analysis was done to excluded patients with EGFRvIII mutation that treated with rindopepimut (**Tables S13**-**15**). And no significantly different results were found.

## Discussion

To date, a number of clinical trials investigating multiple monotherapies or combination therapies of chemotherapy, targeted therapy and immunotherapy for recurrent GBM have been published. Recently, the EANO guidelines pointed out that there are no well-defined standards of care for recurrent GBMs (5). A relatively large number of treatments make it difficult for clinicians to make a proper selection in clinical practice. Nevertheless, no direct comparison of existing regimens is available, and it is difficult to draw conclusions.

The purpose of our network meta-analysis was to synthesize all the available evidence from current research, compare the efficacy and safety of recurrent GBM therapies, and contribute to identifying the more effective therapies among those currently available. To our knowledge, this study represents the most comprehensive overview of the efficiency and safety data available for the treatment of recurrent GBM, and its main findings can be summarized in the following paragraphs.

There are no statistically significant differences among the included therapies in the three efficiency indicators of OS, 6-m PFS, and ORR. This result may be due to the fact that most clinical trials in recurrent GBM did not yield positive results. It is not so realistic to find a rigidly efficient cure for recurrent GBM in the short term, thus analyzing the published results for a better combination is a more practical option. However, considering that it is difficult for a specific therapy to appear in the short term, and clinicians need to choose among the current therapies, we have ranked the current therapies and determined the relatively effective ones.

The analysis results suggested that BEV (10 mg/kg) and CCNU (90 mg/m^2^) combination therapy and regorafenib are relatively superior for improving OS, 6-m PFS, and ORR, and the adverse effects profile of these two therapies is acceptable. Therefore, BEV and CCNU combination therapy and regorafenib monotherapy are recommended in clinical practice considering its relatively high efficiency. The efficacy of regorafenib has also been emphasized in the latest NCCN guideline, where regorafenib is listed as a preferred regimen.

Our results recommend BEV and CCNU combination therapy after comprehensive analysis, but the phase III EORTC 26101 trial in 2017 reported that although PFS is prolonged in the combination group, the effect of combination therapy did not exceed the effect of CCNU monotherapy in OS, and was only listed in other recommended regimens in NCCN guideline that can be considered after BEV monotherapy failure (26). However, combining the two trials statistically, we found BEV and CCNU had a relatively prior efficacy (17, 26), and this should be interpreted imprecisely in the clinical practice due to tremendous heterogeneity of patients with recurrent GBM.

Recently, molecular pathological status shown as a biomarker of treatment and prognosis for patients with GBM. In our research, five trials reported IDH mutation status (17, 19, 20, 23) (IDH mutant patients accounted for 3–10%) and five reported MGMT promoter methylation (patients with MGMT promoter methylation accounted for 37–68%). In terms of a single study conclusion, the phase II BELOB trial showed that both PFS and OS of IDH mutant patients after BEV and CCNU treatment were significantly higher than that of IDH wild-type patients (17). Considering that the proportion of IDH mutant patients was relatively low, their influence on the result analysis was relatively small. At the same time, the studies showed that patients with MGMT promoter methylation received BEV and CCNU combination therapy (17) and onartuzumab plus BEV treatment (23), PFS and OS were significantly higher than those without MGMT promoter methylation, while OS in patients receiving CCNU and BEV or OS-6 of patients treated with fotemustine or BEV was also higher in methylation-positive patients than those without MGMT promoter methylation (20, 26). Therefore, our research suggests possible future clinical research directions, as multiarmed head-to-head RCTs with subgroup analysis, which was descripted in GBM AGILE trial (30).

The EGFRvIII-targeted therapies have become a specific research field in GBM. In previous studies, the peptide vaccine rindopepimut showed benefits in prolonging OS and symptom control in patients with recurrent GBM with EGFRvIII mutation (28). A majority of EGFRvIII-mutant GBMs maintain the mutation at recurrence. However, a subset of patients may experience EGFRvIII expression change at recurrence, thus reassessing the EGFRvIII status for patients with recurrent GBM is recommended before EGFRvIII-targeted therapies (31).

The included trial only reported the efficiency of TTF and BEV combination therapy on OS. The effect of this therapy on prolonging OS is only second to BEV and CCNU combination therapy. Therefore, this treatment is recommended if the family can financially afford the treatment and the patient has good compliance and can receive better care. We suggest that future clinical trials should focus on the impact on PFS and ORR to determine the efficiency of TTF and BEV combination therapy in the treatment of recurrent GBM. We look forward to further reports on the combination therapies of TTF and other drugs.

The combination therapies including BEV were extremely effective in prolonging PFS and ORR, and this result was related to the effect of BEV in controlling symptoms. Thus far, studies have confirmed the role of BEV in alleviating necrosis and brain edema after radiotherapy (32), and it has been proven to be effective in prolonging PFS for patients with recurrent GBM. Therefore, BEV has been recommended as preferred regimens in the latest version of NCCN guidelines, while the BEV-based combination therapies listed as other recommended regimens. This conclusion suggests that combination therapies, including BEV, are of great significance, especially for patients with tumor-related symptoms such as cerebral edema.

In this study, we performed indirect comparisons and comprehensive rankings of therapies that have not been directly compared in clinical trials, which may support clinicians in the efficient health care of recurrent GBM patients and help clinical teams and patients make joint medical decisions, thereby increasing the possibility of prolonging survival and improving treatment effectiveness. Our study also statistically summarized the incidence of adverse events of various therapies, which can assist clinicians in increasing the surveillance, prevention and management of drug-related toxic effects. We also performed the assessment of RCTs heterogeneity, evidence certainty and risk of bias. We found a low-to-moderate heterogeneity between included RCTs, so we can combine the direct and indirect comparisons between those treatments. However, we found a low-to-moderate qualitative level of each evidence chain, because we downgraded all pairwise comparisons by one level for almost half of trials included were sponsored commercially and with a relatively small sample size, and several single-arm clinical trials were not included. Also, a few clinical trials were not included for a various interaction in control group. And this is one of the limitations of our study and of vital importance when we discuss the optimal therapy for recurrent GBM.

Due to the relatively low incidence of GBM and high degree of malignancy and low mean OS in patients, there are indeed several trials adopted single-arm treatment or non-randomized trial design. Our search was unable to include ongoing studies and trials with additional unpublished data, also, single-arm clinical trials, therefore, a considerable amount of meaningful studies were not included. Secondly, the definitions of 6-m PFS and OS were not exactly same among the included studies. To be specific, five studies calculated efficacy from randomization and two studies defined efficacy from start of treatment, while eight articles did not clarify the specific initial node for efficacy evaluation. The inconsistency among researches may bring certain bias to the statistical results. In addition, the included published results lag behind the current therapy option. Besides TTF, new therapies including neoadjuvant checkpoint inhibitor and laser interstitial thermotherapy has not been included. And these advanced therapies remain to be explored in future studies.

Because the network meta-analysis requires the included studies to form an evidence chain, the regimen not involved in other studies cannot be included. In our analysis, there were nine studies that met the inclusion criteria, but there was no corresponding comparable regimen in other trials. Among them, seven compared the efficacy and safety between two therapies, and the other two explored the efficacy of different doses of cilengitide (33) and ABT-888 (34). In the studies comparing different therapies, most of them did not achieve the statistical different results of the primary endpoints (including TMZ and dose-dense TMZ versus PCV regimen (35), carboplatin and RMP-7 combination therapy versus carboplatin (36), TTF vs physician’s choice chemotherapy (37), afatinib monotherapy and afatinib plus TMZ versus TMZ (38), different dose of single-agent CT-322 and irinotecan combination therapy (39), and cintredekin besudotox versus carmustine (40). TMZ and dose-dense TMZ regimens showed no significant survival benefit than PCV. PD-1 inhibitor pembrolizumab improved both OS and PFS compared with only adjuvant therapy (41). The requirement of the evidence chain also results in some high-quality studies not being included for comparison, which to some extent affects the comprehensiveness of the results.

## Data Availability Statement

The original contributions presented in the study are included in the article/**Supplementary Material**. Further inquiries can be directed to the corresponding author.

## Author Contributions

Study design and manuscript writing: YueW, WC, YuW, and WM. Clinical trials searching: YueW, WC, PL, and LL. Model constructing and statistical analysis: YueW, WC, and BZ. Manuscript formatting and revising: YueW and WC. All authors contributed to the article and approved the submitted version.

## Funding

Beijing Municipal Science & Technology Commission [7202150]; Beijing Municipal Science & Technology Commission [19JCZDJC64200(Z)]; Chinese Academy of Medical Sciences [2016-I2M-2-001]; Peking Union Medical College Hospital [2019ZLH101].

## Conflict of Interest

The authors declare that the research was conducted in the absence of any commercial or financial relationships that could be construed as a potential conflict of interest.
