# Supplementary material for: Optimal Therapies for Recurrent Glioblastoma: A Bayesian Network Meta-Analysis
Source: Front Oncol. 2021 Mar 29;11:641878. doi: 10.3389/fonc.2021.641878 (PMC8039381; doi:10.3389/fonc.2021.641878)
Supplement: Supplementary file 2 [file DataSheet_2.docx]

**Supplementary Results**

[1. Excluded multi-arm RCTs 2](#_Toc64592637)

[2. Assessment of model fitting, evidence certainty and heterogeneity and risk of bias 6](#_Toc64592638)

[3. Supplementary results for objective response rate 21](#_Toc64592639)

[4. Supplementary results for 6m-progression free survival rate 26](#_Toc64592640)

[5. Supplementary results for overall survival 31](#_Toc64592641)

[6. Sensitivity analysis 37](#_Toc64592642)

[7. Changes to the protocol 41](#_Toc64592643)

# 1. Excluded multi-arm RCTs

Un: No reports available; PCV: Procarbazine, lomustine, and vincristine; TMZ: Temozolomide; CB: Cintredekin besudotox; GW: Gliadel wafers; TTF: Tumor , ABT-888:, BCNU:, ADV-TK: Adenovirus mutant thymidine kinase.

| Study | Phase | Treatment Arms | Number of Patients | Reason for exclusion |
| --- | --- | --- | --- | --- |
| Michael D. Prados et al. (2003) Neuro-Oncol. (Prados et al., 2003) | II | Carboplatin+RMP-7 | 61 | Cannot be included in the evidence chain |
|  |  | Carboplatin+placebo | 60 |  |
| David A. Reardon et al. (2008) J Clin Oncol. (Reardon et al., 2008) | II | Cilengitide (500mg） | 41 |  |
|  |  | Cilengitide (2000mg） | 40 |  |
| Michael Brada et al. (2010) J Clin Oncol. (Brada et al., 2010) | Un | PCV | 224 |  |
|  |  | TMZ (200mg/m2×5/28) | 112 |  |
|  |  | TMZ (100mg/m2×21/28) | 111 |  |
| Sandeep Kunwar et al. (2010) Neuro-Oncol. (Kunwar et al., 2010) | III | CB | 183 |  |
|  |  | GW | 98 |  |
| Roger Stupp et al. (2012) Eur J Cancer.(Stupp et al., 2012) | II | TTF | 120 |  |
|  |  | Active chemotherapy | 117 |  |
| David A. Reardon et al. (2015) Neuro-Oncol. (Reardon et al., 2015) | II | Afatinib | 41 |  |
|  |  | TMZ+Afatinib | 39 |  |
|  |  | TMZ | 39 |  |
| David Schiff et al. (2015) Invest New Drugs.(Schiff et al., 2015) | II | CT_322 (1 mg/kg) | 14 |  |
|  |  | CT_322 (1 mg/kg)+ irinotecan | 7 |  |
|  |  | CT_322 (2mg/kg) | 24 |  |
|  |  | CT_322 (2mg/kg)+ irinotecan | 16 |  |
| H Ian Robins et al. (2016) J Neurooncol. (Robins et al., 2016) | II | ABT-888+TMZ, BEV native | 73 |  |
|  |  | ABT-888+TMZ, BEV failure | 73 |  |
|  |  | ABT-888+TMZ(150-200 mg/m2×5/28d) , BEV native | 32 |  |
|  |  | ABT-888+TMZ(150-200 mg/m2×5/28d) , BEV failure | 37 |  |
| Timothy F. Cloughesy et al. (2019) Nat Med.(Cloughesy et al., 2019) | Un | Neoadjuvant pembrolizumab | 16 |  |
|  |  | Adjuvant pembrolizumab | 16 |  |
| Martin J. van den Bent et al. (2009) J Clin Oncol. (van den Bent et al., 2009) | II | TMZ or BCNU | 56 | Ambiguous intervention |
|  |  | Erlotinib | 54 |  |
| U. Bogdahn et al. (2011) Neuro-Oncol. (Bogdahn et al., 2011) | II | Trabedersen (10 mmol/L) | 28 |  |
|  |  | Trabedersen (80 mmol/L) | 34 |  |
|  |  | TMZ or PCV | 33 |  |
| J. Duerinck et al. (2016) J Neurooncol. (Duerinck et al., 2016) | II | Axitinib | 22 |  |
|  |  | Physician's best alternative therapy | 22 |  |
| Nan Ji et al. (2016) Oncotarget.(Ji et al., 2016) | II | ADV-TK | 22 |  |
|  |  | Physician's best alternative therapy | 22 |  |
| J. Duerinck et al. (2018) J Neurooncol. (Duerinck et al., 2018) | II | CCNU +Axitinib | 29 |  |
|  |  | Axitinib | 50 |  |
|  |  | Physician's best alternative therapy | 22 |  |
| David A. Reardon et al. (2011) J Neurooncol. (Reardon et al., 2011) | II | BEV+TMZ | 10 | Little sample size |
|  |  | BEV+ Etoposide | 13 |  |
| Daniela A Bota et al. (2018) CNS Oncol. (Bota et al., 2018) | II | ERC1671 | 5 |  |
|  |  | BEV | 4 |  |
| Martin J van den Bent et al. (2018) Lancet oncol. (van den Bent et al., 2018) | II | BEV+TMZ | 78 | Patients included were diagnosed with low-grade glioma |
|  |  | TMZ | 77 |  |

Table S1 RCTs that were excluded.

**References**

Bogdahn, U., Hau, P., Stockhammer, G., Venkataramana, N.K., Mahapatra, A.K., Suri, A., et al. (2011). Targeted therapy for high-grade glioma with the TGF-β2 inhibitor trabedersen: results of a randomized and controlled phase IIb study. *Neuro Oncol* 13(1)**,** 132-142. doi: 10.1093/neuonc/noq142.

Bota, D.A., Chung, J., Dandekar, M., Carrillo, J.A., Kong, X.T., Fu, B.D., et al. (2018). Phase II study of ERC1671 plus bevacizumab versus bevacizumab plus placebo in recurrent glioblastoma: interim results and correlations with CD4(+) T-lymphocyte counts. *CNS Oncol* 7(3)**,** Cns22. doi: 10.2217/cns-2018-0009.

Brada, M., Stenning, S., Gabe, R., Thompson, L.C., Levy, D., Rampling, R., et al. (2010). Temozolomide versus procarbazine, lomustine, and vincristine in recurrent high-grade glioma. *J Clin Oncol* 28(30)**,** 4601-4608. doi: 10.1200/jco.2009.27.1932.

Cloughesy, T.F., Mochizuki, A.Y., Orpilla, J.R., Hugo, W., Lee, A.H., Davidson, T.B., et al. (2019). Neoadjuvant anti-PD-1 immunotherapy promotes a survival benefit with intratumoral and systemic immune responses in recurrent glioblastoma. *Nat Med* 25(3)**,** 477-486. doi: 10.1038/s41591-018-0337-7.

Duerinck, J., Du Four, S., Bouttens, F., Andre, C., Verschaeve, V., Van Fraeyenhove, F., et al. (2018). Randomized phase II trial comparing axitinib with the combination of axitinib and lomustine in patients with recurrent glioblastoma. *J Neurooncol* 136(1)**,** 115-125. doi: 10.1007/s11060-017-2629-z.

Duerinck, J., Du Four, S., Vandervorst, F., D'Haene, N., Le Mercier, M., Michotte, A., et al. (2016). Randomized phase II study of axitinib versus physicians best alternative choice of therapy in patients with recurrent glioblastoma. *J Neurooncol* 128(1)**,** 147-155. doi: 10.1007/s11060-016-2092-2.

Ji, N., Weng, D., Liu, C., Gu, Z., Chen, S., Guo, Y., et al. (2016). Adenovirus-mediated delivery of herpes simplex virus thymidine kinase administration improves outcome of recurrent high-grade glioma. *Oncotarget* 7(4)**,** 4369-4378. doi: 10.18632/oncotarget.6737.

Kunwar, S., Chang, S., Westphal, M., Vogelbaum, M., Sampson, J., Barnett, G., et al. (2010). Phase III randomized trial of CED of IL13-PE38QQR vs Gliadel wafers for recurrent glioblastoma. *Neuro Oncol* 12(8)**,** 871-881. doi: 10.1093/neuonc/nop054.

Prados, M.D., Schold, S.C., Jr., Fine, H.A., Jaeckle, K., Hochberg, F., Mechtler, L., et al. (2003). A randomized, double-blind, placebo-controlled, phase 2 study of RMP-7 in combination with carboplatin administered intravenously for the treatment of recurrent malignant glioma. *Neuro Oncol* 5(2)**,** 96-103. doi: 10.1093/neuonc/5.2.96.

Reardon, D.A., Desjardins, A., Peters, K., Gururangan, S., Sampson, J., Rich, J.N., et al. (2011). Phase II study of metronomic chemotherapy with bevacizumab for recurrent glioblastoma after progression on bevacizumab therapy. *J Neurooncol* 103(2)**,** 371-379. doi: 10.1007/s11060-010-0403-6.

Reardon, D.A., Fink, K.L., Mikkelsen, T., Cloughesy, T.F., O'Neill, A., Plotkin, S., et al. (2008). Randomized phase II study of cilengitide, an integrin-targeting arginine-glycine-aspartic acid peptide, in recurrent glioblastoma multiforme. *J Clin Oncol* 26(34)**,** 5610-5617. doi: 10.1200/jco.2008.16.7510.

Reardon, D.A., Nabors, L.B., Mason, W.P., Perry, J.R., Shapiro, W., Kavan, P., et al. (2015). Phase I/randomized phase II study of afatinib, an irreversible ErbB family blocker, with or without protracted temozolomide in adults with recurrent glioblastoma. *Neuro Oncol* 17(3)**,** 430-439. doi: 10.1093/neuonc/nou160.

Robins, H.I., Zhang, P., Gilbert, M.R., Chakravarti, A., de Groot, J.F., Grimm, S.A., et al. (2016). A randomized phase I/II study of ABT-888 in combination with temozolomide in recurrent temozolomide resistant glioblastoma: an NRG oncology RTOG group study. *J Neurooncol* 126(2)**,** 309-316. doi: 10.1007/s11060-015-1966-z.

Schiff, D., Kesari, S., de Groot, J., Mikkelsen, T., Drappatz, J., Coyle, T., et al. (2015). Phase 2 study of CT-322, a targeted biologic inhibitor of VEGFR-2 based on a domain of human fibronectin, in recurrent glioblastoma. *Invest New Drugs* 33(1)**,** 247-253. doi: 10.1007/s10637-014-0186-2.

Stupp, R., Wong, E.T., Kanner, A.A., Steinberg, D., Engelhard, H., Heidecke, V., et al. (2012). NovoTTF-100A versus physician's choice chemotherapy in recurrent glioblastoma: a randomised phase III trial of a novel treatment modality. *Eur J Cancer* 48(14)**,** 2192-2202. doi: 10.1016/j.ejca.2012.04.011.

van den Bent, M.J., Brandes, A.A., Rampling, R., Kouwenhoven, M.C., Kros, J.M., Carpentier, A.F., et al. (2009). Randomized phase II trial of erlotinib versus temozolomide or carmustine in recurrent glioblastoma: EORTC brain tumor group study 26034. *J Clin Oncol* 27(8)**,** 1268-1274. doi: 10.1200/jco.2008.17.5984.

van den Bent, M.J., Klein, M., Smits, M., Reijneveld, J.C., French, P.J., Clement, P., et al. (2018). Bevacizumab and temozolomide in patients with first recurrence of WHO grade II and III glioma, without 1p/19q co-deletion (TAVAREC): a randomised controlled phase 2 EORTC trial. *Lancet Oncol* 19(9)**,** 1170-1179. doi: 10.1016/s1470-2045(18)30362-0.

# 2. Assessment of model fitting, evidence certainty and heterogeneity and risk of bias

1) Model fit

| Outcome |  | Dbar | pD | DIC | I^2 |
| --- | --- | --- | --- | --- | --- |
| ORR | Random model | 26.29 | 25.12 | 51.42 | 5% |
|  | Fixed model | 26.38 | 25.18 | 51.56 | 5% |
| 6m-PFS rate | Random model | 31.11 | 30.04 | 61.15 | 4% |
|  | Fixed model | 31.95 | 29.08 | 61.03 | 6% |
| OS | Random model | 18.30 | 17.80 | 36.10 | 7% |
|  | Fixed model | 19.89 | 16.95 | 36.84 | 15% |

Table S2 Model fitting parameters of Bayesian network meta-analysis.

2) Between trial heterogeneity τ2

| Outcome | τ^2 | 95%CI |
| --- | --- | --- |
| ORR | 0.16 | 0-0.35 |
| 6m-PFS rate | 0.14 | 0-0.33 |
| OS | 0.11 | 0-0.27 |

Table S3 Estimates of between trial heterogeneity

τ2 estimates indicated that there was low heterogeneity between all trials in ORR, 6m-PFS rate and OS, and between trial heterogeneity was considered adequate for all outcomes.

Presented is the between-trial variance τ2 as a measure of the heterogeneity between trials in the network for each outcome and comparison. A τ2 estimate of 0.04 may be interpreted as a low, 0.14 as a moderate and 0.40 as a substantial degree of heterogeneity between trials. (Trelle et al., 2011)

3) Risk of bias table of included studies

| **Study** | **Sequence Generation** | **Allocation Concealed** | **Blinding Participant** | **Blinding Therapist** | **Blinding Assessor** | **Attrition Bias** | **Reporting Bias** |
| --- | --- | --- | --- | --- | --- | --- | --- |
| **Friedman HS, J Clin Oncol, 2009** | **Low risk of bias** | **Unclear risk of bias** | **High risk of bias** | **High risk of bias** | **High risk of bias** | **Low risk of bias** | **Low risk of bias** |
| **Wolfgang Wick, J Clin Oncol, 2010** | **Unclear risk of bias** | **Unclear risk of bias** | **High risk of bias** | **Unclear risk of bias** | **Low risk of bias** | **Low risk of bias** | **Low risk of bias** |
| **Tracy T. Batchelor, J Clin Oncol, 2013** | **Unclear risk of bias** | **Unclear risk of bias** | **High risk of bias** | **High risk of bias** | **Low risk of bias** | **Low risk of bias** | **Low risk of bias** |
| **Walter Taal, Lancet Oncol, 2014** | **Low risk of bias** | **Unclear risk of bias** | **High risk of bias** | **High risk of bias** | **Unclear risk of bias** | **Low risk of bias** | **Low risk of bias** |
| **Kathryn M. Field, Neuro-Oncology, 2015** | **Unclear risk of bias** | **Unclear risk of bias** | **High risk of bias** | **High risk of bias** | **Unclear risk of bias** | **Low risk of bias** | **Low risk of bias** |
| **Alba A. Brandes, Neuro-Oncology, 2016 (galunisertib plus lomustine v.s. galunisertib v.s. lomustine plus placebo)** | **Low risk of bias** | **Low risk of bias** | **Low risk of bias** | **Low risk of bias** | **Unclear risk of bias** | **Low risk of bias** | **Low risk of bias** |
| **Alba A. Brandes, Neuro-Oncology, 2016 (bevacizumab v.s. fotemustine)** | **Unclear risk of bias** | **Unclear risk of bias** | **Unclear risk of bias** | **Unclear risk of bias** | **Unclear risk of bias** | **Low risk of bias** | **Low risk of bias** |
| **Nicholas Brown, Plos One, 2016** | **Low risk of bias** | **Low risk of bias** | **Low risk of bias** | **Low risk of bias** | **Unclear risk of bias** | **Low risk of bias** | **Low risk of bias** |
| **Shiao-Pei Weathers, J Neuro-Oncol, 2016** | **Unclear risk of bias** | **Low risk of bias** | **High risk of bias** | **Unclear risk of bias** | **Unclear risk of bias** | **Low risk of bias** | **Low risk of bias** |
| **Timothy Cloughesy, J Clin Oncol, 2017** | **Unclear risk of bias** | **Unclear risk of bias** | **Low risk of bias** | **Unclear risk of bias** | **Unclear risk of bias** | **Unclear risk of bias** | **Low risk of bias** |
| **Mark R. Gilbert, J Neuro-Oncol,2017** | **Low risk of bias** | **Low risk of bias** | **Low risk of bias** | **Low risk of bias** | **Unclear risk of bias** | **Low risk of bias** | **Low risk of bias** |
| **Santosh Kesari, Clinical Trial Evaluation, 2017** | **Unclear risk of bias** | **Unclear risk of bias** | **Low risk of bias** | **Unclear risk of bias** | **Unclear risk of bias** | **Unclear risk of bias** | **Low risk of bias** |
| **Giuseppe Lombardi, Lancet Oncol, 2018** | **Low risk of bias** | **Low risk of bias** | **High risk of bias** | **High risk of bias** | **High risk of bias** | **Low risk of bias** | **Low risk of bias** |
| **Wolfgang Wick, N Engl J Med, 2017** | **Low risk of bias** | **Low risk of bias** | **Unclear risk of bias** | **Unclear risk of bias** | **Unclear risk of bias** | **Low risk of bias** | **Low risk of bias** |
| **David A. Reardon, Clin Cancer Res, 2020** | **Low risk of bias** | **Low risk of bias** | **Low risk of bias** | **Low risk of bias** | **Unclear risk of bias** | **Low risk of bias** | **Low risk of bias** |

Table S4 Risk of bias assessment of all RCTs included

4）GRADE of evidence

The qualitative evaluation of evidence was done followed GRADE guideline (Puhan et al., 2014), and each estimate was evaluated according to the following criteria, including limitation, inconsistency, indirectness and publication bias. (1) Limitations: we downgraded by one level if any comparisons had a high risk of bias. (2) Inconsistency: we estimated heterogeneity between trials before and found low-to-moderate level of heterogeneity, so we did not downgrade by heterogeneity. And for inconsistency, we downgraded by one level if significantly inconsistency was shown by side splitting. (3) Indirectness: we downgraded by one level for evidence chain that include rindopepimut plus bevacizumab, because it was researched in patients with EGFRvIII mutation. As for others, we did not downgrade for limited patients in recurrent glioblastoma and comparable baseline characteristics. (4) Publication bias: we downgraded all pairwise comparisons by one level, because almost half of trials included were sponsored commercially and with a relatively small sample size, and several single-arm clinical trials were not included. Also, a few clinical trials were not included for a various interaction in control group.

Considering recently published GRADE guideline, we did not include “Imprecision” as one of the criteria (Brignardello-Petersen et al., 2018).

a. ORR

|  | Direct evidence | | Indirect evidence | | Network meta-analysis | |
| --- | --- | --- | --- | --- | --- | --- |
| Comparison | Risk ratio (95% credible interval) | Quality of evidence | Risk ratio (95% credible interval) | Quality of evidence | Risk ratio (95% credible interval) | Quality of evidence |
| BEV vs BEV5_CCNU90 | 1.56 (-1.19, 4.47) | LOW^#&^ | - | - | 1.56 (-1.19, 4.47) | LOW |
| BEV vs BEV_CAR | -0.56 (-3.18, 2.03) | LOW^#&^ | - | - | -0.56 (-3.18, 2.03) | LOW |
| BEV vs BEV_CCNU | 0.17 (-2.23, 2.64) | LOW^#&^ | - | - | 0.17 (-2.23, 2.64) | LOW |
| BEV vs BEV_IRI | -0.26 (-2.71, 2.14) | LOW^#&^ | - | - | -0.26 (-2.71, 2.14) | LOW |
| BEV vs BEV_ONA | 0.2 (-2.26, 2.67) | MODERATE^&^ | - | - | 0.2 (-2.26, 2.67) | MODERATE |
| BEV vs BEV_TMZ | - | - | 0.22 (-3.29, 3.62) | LOW^#&^ | 0.22 (-3.29, 3.62) | LOW |
| BEV vs CCNU | 2.33 (-0.31, 5.28) | LOW^#&^ | - | - | 2.33 (-0.31, 5.28) | LOW |
| BEV vs CED | - | - | 1.89 (-1.79, 5.71) | LOW^#&^ | 1.89 (-1.79, 5.71) | LOW |
| BEV vs CED_CCNU | - | - | 1.73 (-1.9, 5.52) | LOW^#&^ | 1.73 (-1.9, 5.52) | LOW |
| BEV vs CED_GEF | - | - | 1.55 (-2.95, 6.13) | LOW^#&^ | 1.55 (-2.95, 6.13) | LOW |
| BEV vs ENZ | - | - | 2.98 (-0.77, 6.9) | LOW^#&^ | 2.98 (-0.77, 6.9) | LOW |
| BEV vs FOT | 1.26 (-1.3, 3.94) | MODERATE^&^ | - | - | 1.26 (-1.3, 3.94) | MODERATE |
| BEV vs REG | - | - | 2.35 (-1.54, 6.39) | LOW^#&^ | 2.35 (-1.54, 6.39) | LOW |
| BEV vs RIN_BEV | -0.41 (-2.94, 2.08) | LOW^%&^ | - | - | -0.41 (-2.94, 2.08) | LOW |
| BEV5_CCNU90 vs BEV_CAR | - | - | 2.13 (-1.65, 5.98) | LOW^#&^ | 2.13 (-1.65, 5.98) | LOW |
| BEV5_CCNU90 vs BEV_CCNU | - | - | 1.39 (-2.31, 5.15) | LOW^#&^ | 1.39 (-2.31, 5.15) | LOW |
| BEV5_CCNU90 vs BEV_IRI | - | - | 1.86 (-1.83, 5.67) | LOW^#&^ | 1.86 (-1.83, 5.67) | LOW |
| BEV5_CCNU90 vs BEV_ONA | - | - | 1.37 (-2.33, 5.16) | LOW^#&^ | 1.37 (-2.33, 5.16) | LOW |
| BEV5_CCNU90 vs BEV_TMZ | - | - | 1.37 (-3.03, 5.88) | LOW^#&^ | 1.37 (-3.03, 5.88) | LOW |
| BEV5_CCNU90 vs CCNU | - | - | 0.78 (-3.13, 4.83) | LOW^#&^ | 0.78 (-3.13, 4.83) | LOW |
| BEV5_CCNU90 vs CED | - | - | 0.3 (-4.34, 5.07) | LOW^#&^ | 0.3 (-4.34, 5.07) | LOW |
| BEV5_CCNU90 vs CED_CCNU | - | - | 0.15 (-4.46, 4.91) | LOW^#&^ | 0.15 (-4.46, 4.91) | LOW |
| BEV5_CCNU90 vs CED_GEF | - | - | -0.04 (-5.32, 5.31) | LOW^#&^ | -0.04 (-5.32, 5.31) | LOW |
| BEV5_CCNU90 vs ENZ | - | - | 1.41 (-3.37, 6.25) | LOW^#&^ | 1.41 (-3.37, 6.25) | LOW |
| BEV5_CCNU90 vs FOT | - | - | -0.29 (-4.19, 3.59) | LOW^#&^ | -0.29 (-4.19, 3.59) | LOW |
| BEV5_CCNU90 vs REG | - | - | 0.76 (-4, 5.7) | LOW^#&^ | 0.76 (-4, 5.7) | LOW |
| BEV5_CCNU90 vs RIN_BEV | - | - | -2 (-5.81, 1.74) | VERY LOW^#%&^ | -2 (-5.81, 1.74) | VERY LOW |
| BEV_CAR vs BEV_CCNU | - | - | 0.76 (-2.78, 4.32) | LOW^#&^ | 0.76 (-2.78, 4.32) | LOW |
| BEV_CAR vs BEV_IRI | - | - | 0.29 (-3.3, 3.82) | LOW^#&^ | 0.29 (-3.3, 3.82) | LOW |
| BEV_CAR vs BEV_ONA | - | - | 0.76 (-2.78, 4.34) | LOW^#&^ | 0.76 (-2.78, 4.34) | LOW |
| BEV_CAR vs BEV_TMZ | - | - | 0.77 (-3.59, 5.08) | LOW^#&^ | 0.77 (-3.59, 5.08) | LOW |
| BEV_CAR vs CCNU | - | - | 2.93 (-0.8, 6.81) | LOW^#&^ | 2.93 (-0.8, 6.81) | LOW |
| BEV_CAR vs CED | - | - | 2.47 (-2.05, 7.07) | LOW^#&^ | 2.47 (-2.05, 7.07) | LOW |
| BEV_CAR vs CED_CCNU | - | - | 2.29 (-2.19, 6.91) | LOW^#&^ | 2.29 (-2.19, 6.91) | LOW |
| BEV_CAR vs CED_GEF | - | - | 2.14 (-3.07, 7.32) | LOW^#&^ | 2.14 (-3.07, 7.32) | LOW |
| BEV_CAR vs ENZ | - | - | 3.58 (-1, 8.27) | LOW^#&^ | 3.58 (-1, 8.27) | LOW |
| BEV_CAR vs FOT | - | - | 1.85 (-1.81, 5.56) | LOW^#&^ | 1.85 (-1.81, 5.56) | LOW |
| BEV_CAR vs REG | - | - | 2.91 (-1.76, 7.74) | LOW^#&^ | 2.91 (-1.76, 7.74) | LOW |
| BEV_CAR vs RIN_BEV | - | - | 0.15 (-3.48, 3.79) | VERY LOW^#%&^ | 0.15 (-3.48, 3.79) | VERY LOW |
| BEV_CCNU vs BEV_IRI | - | - | -0.44 (-3.92, 3) | LOW^#&^ | -0.44 (-3.92, 3) | LOW |
| BEV_CCNU vs BEV_ONA | - | - | 0.02 (-3.44, 3.51) | LOW^#&^ | 0.02 (-3.44, 3.51) | LOW |
| BEV_CCNU vs BEV_TMZ | - | - | 0.04 (-4.27, 4.28) | LOW^#&^ | 0.04 (-4.27, 4.28) | LOW |
| BEV_CCNU vs CCNU | 2.15 (-0.52, 5.13) | MODERATE^&^ | - | - | 2.15 (-0.52, 5.13) | MODERATE |
| BEV_CCNU vs CED | - | - | 1.7 (-1.98, 5.62) | LOW^#&^ | 1.7 (-1.98, 5.62) | LOW |
| BEV_CCNU vs CED_CCNU | - | - | 1.54 (-2.1, 5.39) | LOW^#&^ | 1.54 (-2.1, 5.39) | LOW |
| BEV_CCNU vs CED_GEF | - | - | 1.36 (-3.07, 5.94) | LOW^#&^ | 1.36 (-3.07, 5.94) | LOW |
| BEV_CCNU vs ENZ | - | - | 2.8 (-1.02, 6.74) | LOW^#&^ | 2.8 (-1.02, 6.74) | LOW |
| BEV_CCNU vs FOT | - | - | 1.08 (-2.45, 4.69) | LOW^#&^ | 1.08 (-2.45, 4.69) | LOW |
| BEV_CCNU vs REG | - | - | 2.18 (-1.75, 6.23) | LOW^#&^ | 2.18 (-1.75, 6.23) | LOW |
| BEV_CCNU vs RIN_BEV | - | - | -0.6 (-4.12, 2.88) | VERY LOW^#%&^ | -0.6 (-4.12, 2.88) | VERY LOW |
| BEV_IRI vs BEV_ONA | - | - | 0.47 (-2.98, 3.97) | LOW^#&^ | 0.47 (-2.98, 3.97) | LOW |
| BEV_IRI vs BEV_TMZ | 0.49 (-1.97, 2.94) | MODERATE^&^ | - | - | 0.49 (-1.97, 2.94) | MODERATE |
| BEV_IRI vs CCNU | - | - | 2.62 (-0.93, 6.41) | LOW^#&^ | 2.62 (-0.93, 6.41) | LOW |
| BEV_IRI vs CED | - | - | 2.17 (-2.23, 6.67) | LOW^#&^ | 2.17 (-2.23, 6.67) | LOW |
| BEV_IRI vs CED_CCNU | - | - | 2.01 (-2.4, 6.54) | LOW^#&^ | 2.01 (-2.4, 6.54) | LOW |
| BEV_IRI vs CED_GEF | - | - | 1.82 (-3.23, 6.97) | LOW^#&^ | 1.82 (-3.23, 6.97) | LOW |
| BEV_IRI vs ENZ | - | - | 3.25 (-1.22, 7.88) | LOW^#&^ | 3.25 (-1.22, 7.88) | LOW |
| BEV_IRI vs FOT | - | - | 1.53 (-2.03, 5.14) | LOW^#&^ | 1.53 (-2.03, 5.14) | LOW |
| BEV_IRI vs REG | - | - | 2.64 (-1.96, 7.38) | LOW^#&^ | 2.64 (-1.96, 7.38) | LOW |
| BEV_IRI vs RIN_BEV | - | - | -0.14 (-3.67, 3.31) | VERY LOW^#%&^ | -0.14 (-3.67, 3.31) | VERY LOW |
| BEV_ONA vs BEV_TMZ | - | - | 0.01 (-4.29, 4.24) | LOW^#&^ | 0.01 (-4.29, 4.24) | LOW |
| BEV_ONA vs CCNU | - | - | 2.14 (-1.46, 5.95) | LOW^#&^ | 2.14 (-1.46, 5.95) | LOW |
| BEV_ONA vs CED | - | - | 1.68 (-2.73, 6.26) | LOW^#&^ | 1.68 (-2.73, 6.26) | LOW |
| BEV_ONA vs CED_CCNU | - | - | 1.52 (-2.81, 6.08) | LOW^#&^ | 1.52 (-2.81, 6.08) | LOW |
| BEV_ONA vs CED_GEF | - | - | 1.33 (-3.75, 6.48) | LOW^#&^ | 1.33 (-3.75, 6.48) | LOW |
| BEV_ONA vs ENZ | - | - | 2.79 (-1.72, 7.4) | LOW^#&^ | 2.79 (-1.72, 7.4) | LOW |
| BEV_ONA vs FOT | - | - | 1.07 (-2.5, 4.72) | MODERATE^&^ | 1.07 (-2.5, 4.72) | MODERATE |
| BEV_ONA vs REG_BEV | - | - | 2.14 (-2.43, 6.91) | LOW^%&^ | 2.14 (-2.43, 6.91) | LOW |
| BEV_ONA vs RIN_BEV | - | - | -0.62 (-4.19, 2.91) | LOW^%&^ | -0.62 (-4.19, 2.91) | LOW |
| BEV_TMZ vs CCNU | - | - | 2.14 (-2.18, 6.67) | LOW^#&^ | 2.14 (-2.18, 6.67) | LOW |
| BEV_TMZ vs CED | - | - | 1.69 (-3.34, 6.85) | LOW^#&^ | 1.69 (-3.34, 6.85) | LOW |
| BEV_TMZ vs CED_CCNU | - | - | 1.52 (-3.46, 6.69) | LOW^#&^ | 1.52 (-3.46, 6.69) | LOW |
| BEV_TMZ vs CED_GEF | - | - | 1.34 (-4.31, 7.04) | LOW^#&^ | 1.34 (-4.31, 7.04) | LOW |
| BEV_TMZ vs ENZ | - | - | 2.77 (-2.34, 8) | LOW^#&^ | 2.77 (-2.34, 8) | LOW |
| BEV_TMZ vs FOT | - | - | 1.05 (-3.25, 5.44) | LOW^#&^ | 1.05 (-3.25, 5.44) | LOW |
| BEV_TMZ vs REG | - | - | 2.17 (-3.03, 7.47) | LOW^#&^ | 2.17 (-3.03, 7.47) | LOW |
| BEV_TMZ vs RIN_BEV | - | - | -0.64 (-4.88, 3.68) | VERY LOW^#%&^ | -0.64 (-4.88, 3.68) | VERY LOW |
| CCNU vs CED | -0.45 (-2.99, 2.03) | LOW^#&^ | - | - | -0.45 (-2.99, 2.03) | LOW |
| CCNU vs CED_CCNU | -0.63 (-3.15, 1.84) | LOW^#&^ | - | - | -0.63 (-3.15, 1.84) | LOW |
| CCNU vs CED_GEF | - | - | -0.79 (-4.38, 2.71) | LOW^#&^ | -0.79 (-4.38, 2.71) | LOW |
| CCNU vs ENZ | 0.64 (-2.01, 3.28) | LOW^#&^ | - | - | 0.64 (-2.01, 3.28) | LOW |
| CCNU vs FOT | - | - | -1.08 (-4.97, 2.66) | LOW^#&^ | -1.08 (-4.97, 2.66) | LOW |
| CCNU vs REG | -0.01 (-2.87, 2.84) | MODERATE^&^ | - | - | -0.01 (-2.87, 2.84) | MODERATE |
| CCNU vs RIN_BEV | - | - | -2.78 (-6.64, 0.87) | VERY LOW^#%&^ | -2.78 (-6.64, 0.87) | VERY LOW |
| CED vs CED_CCNU | -0.17 (-2.6, 2.27) | LOW^#&^ | - | - | -0.17 (-2.6, 2.27) | LOW |
| CED vs CED_GEF | -0.35 (-2.85, 2.14) | LOW^#&^ | - | - | -0.35 (-2.85, 2.14) | LOW |
| CED vs ENZ | - | - | 1.1 (-2.55, 4.74) | LOW^#&^ | 1.1 (-2.55, 4.74) | LOW |
| CED vs FOT | - | - | -0.61 (-5.26, 3.86) | LOW^#&^ | -0.61 (-5.26, 3.86) | LOW |
| CED vs REG | - | - | 0.46 (-3.31, 4.26) | LOW^#&^ | 0.46 (-3.31, 4.26) | LOW |
| CED vs RIN_BEV | - | - | -2.33 (-6.92, 2.11) | VERY LOW^#%&^ | -2.33 (-6.92, 2.11) | VERY LOW |
| CED_CCNU vs CED_GEF | - | - | -0.17 (-3.68, 3.29) | LOW^#&^ | -0.17 (-3.68, 3.29) | LOW |
| CED_CCNU vs ENZ | - | - | 1.28 (-2.42, 4.88) | LOW^#&^ | 1.28 (-2.42, 4.88) | LOW |
| CED_CCNU vs FOT | - | - | -0.43 (-5.03, 4.04) | LOW^#&^ | -0.43 (-5.03, 4.04) | LOW |
| CED_CCNU vs REG | - | - | 0.64 (-3.19, 4.41) | LOW^#&^ | 0.64 (-3.19, 4.41) | LOW |
| CED_CCNU vs RIN_BEV | - | - | -2.16 (-6.76, 2.25) | VERY LOW^#%&^ | -2.16 (-6.76, 2.25) | VERY LOW |
| CED_GEF vs ENZ | - | - | 1.44 (-2.96, 5.89) | LOW^#&^ | 1.44 (-2.96, 5.89) | LOW |
| CED_GEF vs FOT | - | - | -0.27 (-5.52, 4.91) | LOW^#&^ | -0.27 (-5.52, 4.91) | LOW |
| CED_GEF vs REG | - | - | 0.82 (-3.71, 5.41) | LOW^#&^ | 0.82 (-3.71, 5.41) | LOW |
| CED_GEF vs RIN_BEV | - | - | -1.98 (-7.2, 3.13) | VERY LOW^#%&^ | -1.98 (-7.2, 3.13) | VERY LOW |
| ENZ vs FOT | - | - | -1.71 (-6.42, 2.87) | LOW^#&^ | -1.71 (-6.42, 2.87) | LOW |
| ENZ vs REG | - | - | -0.64 (-4.51, 3.3) | LOW^#&^ | -0.64 (-4.51, 3.3) | LOW |
| ENZ vs RIN_BEV | - | - | -3.45 (-8.07, 1.1) | VERY LOW^#%&^ | -3.45 (-8.07, 1.1) | VERY LOW |
| FOT vs REG | - | - | 1.07 (-3.65, 5.94) | LOW^#&^ | 1.07 (-3.65, 5.94) | LOW |
| FOT vs RIN_BEV | - | - | -1.71 (-5.4, 1.89) | LOW^%&^ | -1.71 (-5.4, 1.89) | LOW |
| REG vs RIN_BEV | - | - | -2.79 (-7.57, 1.85) | VERY LOW^#%&^ | -2.79 (-7.57, 1.85) | VERY LOW |
| # limitation, $ inconsistency, % indirectness, & publication bias | | | | | | |

Table S 5 GRADE valuation of evidence of ORR

b. 6m PFS rate

|  | Direct evidence | | Indirect evidence | | Network meta-analysis | |
| --- | --- | --- | --- | --- | --- | --- |
| Comparison | Risk ratio (95% credible interval) | Quality of evidence | Risk ratio (95% credible interval) | Quality of evidence | Risk ratio (95% credible interval) | Quality of evidence |
| BEV vs BEV5_CCNU90 | -0.39 (-1.81, 1.04) | LOW^#&^ | - | - | -0.39 (-1.81, 1.04) | LOW |
| BEV vs BEV_CAR | 0.08 (-1.41, 1.55) | LOW^#&^ | - | - | 0.08 (-1.41, 1.55) | LOW |
| BEV vs BEV_CCNU | -0.73 (-2.06, 0.6) | LOW^#&^ | - | - | -0.73 (-2.06, 0.6) | LOW |
| BEV vs BEV_IRI | -0.14 (-1.45, 1.16) | LOW^#&^ | - | - | -0.14 (-1.45, 1.16) | LOW |
| BEV vs BEV_ONA | -0.13 (-1.47, 1.19) | MODERATE^&^ | - | - | -0.13 (-1.47, 1.19) | MODERATE |
| BEV vs BEV_TMZ | - | - | -0.1 (-1.95, 1.73) | LOW^#&^ | -0.1 (-1.95, 1.73) | LOW |
| BEV vs CCNU | 0.04 (-1.26, 1.5) | LOW^#&^ | - | - | 0.04 (-1.26, 1.5) | LOW |
| BEV vs CED | - | - | 0.46 (-1.46, 2.51) | LOW^#&^ | 0.46 (-1.46, 2.51) | LOW |
| BEV vs CED_CCNU | - | - | -0.23 (-2.1, 1.79) | LOW^#&^ | -0.23 (-2.1, 1.79) | LOW |
| BEV vs CED_GEF | - | - | 0.72 (-1.94, 3.56) | LOW^#&^ | 0.72 (-1.94, 3.56) | LOW |
| BEV vs ENZ | - | - | 0.53 (-1.32, 2.56) | LOW^#&^ | 0.53 (-1.32, 2.56) | LOW |
| BEV vs FOT | 1.18 (-0.4, 3.02) | MODERATE^&^ | - | - | 1.18 (-0.4, 3.02) | MODERATE |
| BEV vs GAL | - | - | -0.8 (-3.15, 1.5) | LOW^#&^ | -0.8 (-3.15, 1.5) | LOW |
| BEV vs GAL_CCNU | - | - | 0.09 (-2.27, 2.44) | LOW^#&^ | 0.09 (-2.27, 2.44) | LOW |
| BEV vs REG | - | - | -0.54 (-2.56, 1.62) | LOW^#&^ | -0.54 (-2.56, 1.62) | LOW |
| BEV vs RIN_BEV | -0.41 (-1.9, 1.05) | LOW^%&^ | - | - | -0.41 (-1.9, 1.05) | LOW |
| BEV5_CCNU90 vs BEV_CAR | - | - | -0.46 (-2.52, 1.58) | LOW^#&^ | -0.46 (-2.52, 1.58) | LOW |
| BEV5_CCNU90 vs BEV_CCNU | - | - | 0.36 (-1.61, 2.28) | LOW^#&^ | 0.36 (-1.61, 2.28) | LOW |
| BEV5_CCNU90 vs BEV_IRI | - | - | -0.25 (-2.16, 1.67) | LOW^#&^ | -0.25 (-2.16, 1.67) | LOW |
| BEV5_CCNU90 vs BEV_ONA | - | - | -0.26 (-2.18, 1.69) | LOW^#&^ | -0.26 (-2.18, 1.69) | LOW |
| BEV5_CCNU90 vs BEV_TMZ | - | - | -0.29 (-2.56, 2.05) | LOW^#&^ | -0.29 (-2.56, 2.05) | LOW |
| BEV5_CCNU90 vs CCNU | - | - | 0.41 (-1.48, 2.49) | LOW^#&^ | 0.41 (-1.48, 2.49) | LOW |
| BEV5_CCNU90 vs CED | - | - | 0.83 (-1.5, 3.36) | LOW^#&^ | 0.83 (-1.5, 3.36) | LOW |
| BEV5_CCNU90 vs CED_CCNU | - | - | 0.15 (-2.17, 2.65) | LOW^#&^ | 0.15 (-2.17, 2.65) | LOW |
| BEV5_CCNU90 vs CED_GEF | - | - | 1.09 (-1.91, 4.29) | LOW^#&^ | 1.09 (-1.91, 4.29) | LOW |
| BEV5_CCNU90 vs ENZ | - | - | 0.91 (-1.41, 3.42) | LOW^#&^ | 0.91 (-1.41, 3.42) | LOW |
| BEV5_CCNU90 vs FOT | - | - | 1.57 (-0.52, 3.88) | LOW^#&^ | 1.57 (-0.52, 3.88) | LOW |
| BEV5_CCNU90 vs GAL | - | - | -0.41 (-3.14, 2.29) | LOW^#&^ | -0.41 (-3.14, 2.29) | LOW |
| BEV5_CCNU90 vs GAL_CCNU | - | - | 0.48 (-2.27, 3.25) | LOW^#&^ | 0.48 (-2.27, 3.25) | LOW |
| BEV5_CCNU90 vs REG | - | - | -0.15 (-2.61, 2.42) | LOW^#&^ | -0.15 (-2.61, 2.42) | LOW |
| BEV5_CCNU90 vs RIN_BEV | - | - | -0.01 (-2.09, 2.02) | VERY LOW^#%&^ | -0.01 (-2.09, 2.02) | VERY LOW |
| BEV_CAR vs BEV_CCNU | - | - | -0.83 (-2.78, 1.2) | LOW^#&^ | -0.83 (-2.78, 1.2) | LOW |
| BEV_CAR vs BEV_IRI | - | - | -0.23 (-2.18, 1.74) | LOW^#&^ | -0.23 (-2.18, 1.74) | LOW |
| BEV_CAR vs BEV_ONA | - | - | -0.21 (-2.19, 1.79) | LOW^#&^ | -0.21 (-2.19, 1.79) | LOW |
| BEV_CAR vs BEV_TMZ | - | - | -0.19 (-2.55, 2.16) | LOW^#&^ | -0.19 (-2.55, 2.16) | LOW |
| BEV_CAR vs CCNU | - | - | -0.05 (-1.98, 2.07) | LOW^#&^ | -0.05 (-1.98, 2.07) | LOW |
| BEV_CAR vs CED | - | - | 0.37 (-2, 2.94) | LOW^#&^ | 0.37 (-2, 2.94) | LOW |
| BEV_CAR vs CED_CCNU | - | - | -0.31 (-2.65, 2.22) | LOW^#&^ | -0.31 (-2.65, 2.22) | LOW |
| BEV_CAR vs CED_GEF | - | - | 0.63 (-2.41, 3.87) | LOW^#&^ | 0.63 (-2.41, 3.87) | LOW |
| BEV_CAR vs ENZ | - | - | 0.45 (-1.88, 3) | LOW^#&^ | 0.45 (-1.88, 3) | LOW |
| BEV_CAR vs FOT | - | - | 1.11 (-1.05, 3.46) | LOW^#&^ | 1.11 (-1.05, 3.46) | LOW |
| BEV_CAR vs GAL | - | - | -0.88 (-3.64, 1.87) | LOW^#&^ | -0.88 (-3.64, 1.87) | LOW |
| BEV_CAR vs GAL_CCNU | - | - | 0.01 (-2.75, 2.8) | LOW^#&^ | 0.01 (-2.75, 2.8) | LOW |
| BEV_CAR vs REG | - | - | -0.62 (-3.13, 2.01) | LOW^#&^ | -0.62 (-3.13, 2.01) | LOW |
| BEV_CAR vs RIN_BEV | - | - | -0.5 (-2.59, 1.6) | VERY LOW^#%&^ | -0.5 (-2.59, 1.6) | VERY LOW |
| BEV_CCNU vs BEV_IRI | - | - | 0.59 (-1.3, 2.43) | LOW^#&^ | 0.59 (-1.3, 2.43) | LOW |
| BEV_CCNU vs BEV_ONA | - | - | 0.61 (-1.3, 2.46) | LOW^#&^ | 0.61 (-1.3, 2.46) | LOW |
| BEV_CCNU vs BEV_TMZ | - | - | 0.63 (-1.67, 2.88) | LOW^#&^ | 0.63 (-1.67, 2.88) | LOW |
| BEV_CCNU vs CCNU | 0.76 (-0.13, 1.81) | MODERATE^&^ | - | - | 0.76 (-0.13, 1.81) | MODERATE |
| BEV_CCNU vs CED | - | - | 1.19 (-0.46, 2.98) | LOW^#&^ | 1.19 (-0.46, 2.98) | LOW |
| BEV_CCNU vs CED_CCNU | - | - | 0.5 (-1.11, 2.27) | LOW^#&^ | 0.5 (-1.11, 2.27) | LOW |
| BEV_CCNU vs CED_GEF | - | - | 1.44 (-1.01, 4.09) | LOW^#&^ | 1.44 (-1.01, 4.09) | LOW |
| BEV_CCNU vs ENZ | - | - | 1.27 (-0.35, 3.02) | LOW^#&^ | 1.27 (-0.35, 3.02) | LOW |
| BEV_CCNU vs FOT | - | - | 1.93 (-0.17, 4.16) | LOW^#&^ | 1.93 (-0.17, 4.16) | LOW |
| BEV_CCNU vs GAL | - | - | -0.06 (-2.2, 2.02) | LOW^#&^ | -0.06 (-2.2, 2.02) | LOW |
| BEV_CCNU vs GAL_CCNU | - | - | 0.84 (-1.37, 2.94) | LOW^#&^ | 0.84 (-1.37, 2.94) | LOW |
| BEV_CCNU vs REG | - | - | 0.21 (-1.6, 2.07) | LOW^#&^ | 0.21 (-1.6, 2.07) | LOW |
| BEV_CCNU vs RIN_BEV | - | - | 0.33 (-1.68, 2.28) | VERY LOW^#%&^ | 0.33 (-1.68, 2.28) | VERY LOW |
| BEV_IRI vs BEV_ONA | - | - | 0.01 (-1.85, 1.87) | LOW^#&^ | 0.01 (-1.85, 1.87) | LOW |
| BEV_IRI vs BEV_TMZ | 0.04 (-1.28, 1.36) | MODERATE^&^ | - | - | 0.04 (-1.28, 1.36) | MODERATE |
| BEV_IRI vs CCNU | - | - | 0.18 (-1.63, 2.16) | LOW^#&^ | 0.18 (-1.63, 2.16) | LOW |
| BEV_IRI vs CED | - | - | 0.6 (-1.69, 3.05) | LOW^#&^ | 0.6 (-1.69, 3.05) | LOW |
| BEV_IRI vs CED_CCNU | - | - | -0.08 (-2.36, 2.35) | LOW^#&^ | -0.08 (-2.36, 2.35) | LOW |
| BEV_IRI vs CED_GEF | - | - | 0.86 (-2.08, 4.01) | LOW^#&^ | 0.86 (-2.08, 4.01) | LOW |
| BEV_IRI vs ENZ | - | - | 0.68 (-1.57, 3.12) | LOW^#&^ | 0.68 (-1.57, 3.12) | LOW |
| BEV_IRI vs FOT | - | - | 1.32 (-0.71, 3.59) | LOW^#&^ | 1.32 (-0.71, 3.59) | LOW |
| BEV_IRI vs GAL | - | - | -0.66 (-3.32, 2) | LOW^#&^ | -0.66 (-3.32, 2) | LOW |
| BEV_IRI vs GAL_CCNU | - | - | 0.23 (-2.43, 2.95) | LOW^#&^ | 0.23 (-2.43, 2.95) | LOW |
| BEV_IRI vs REG | - | - | -0.39 (-2.78, 2.12) | LOW^#&^ | -0.39 (-2.78, 2.12) | LOW |
| BEV_IRI vs RIN_BEV | - | - | -0.27 (-2.26, 1.68) | VERY LOW^#%&^ | -0.27 (-2.26, 1.68) | VERY LOW |
| BEV_ONA vs BEV_TMZ | - | - | 0.03 (-2.24, 2.3) | LOW^#&^ | 0.03 (-2.24, 2.3) | LOW |
| BEV_ONA vs CCNU | - | - | 0.16 (-1.64, 2.18) | LOW^#&^ | 0.16 (-1.64, 2.18) | LOW |
| BEV_ONA vs CED | - | - | 0.58 (-1.71, 3.06) | LOW^#&^ | 0.58 (-1.71, 3.06) | LOW |
| BEV_ONA vs CED_CCNU | - | - | -0.09 (-2.35, 2.37) | LOW^#&^ | -0.09 (-2.35, 2.37) | LOW |
| BEV_ONA vs CED_GEF | - | - | 0.83 (-2.1, 4.01) | LOW^#&^ | 0.83 (-2.1, 4.01) | LOW |
| BEV_ONA vs ENZ | - | - | 0.67 (-1.57, 3.13) | LOW^#&^ | 0.67 (-1.57, 3.13) | LOW |
| BEV_ONA vs FOT | - | - | 1.32 (-0.74, 3.59) | MODERATE^&^ | 1.32 (-0.74, 3.59) | MODERATE |
| BEV_ONA vs GAL | - | - | -0.67 (-3.32, 2.02) | LOW^#&^ | -0.67 (-3.32, 2.02) | LOW |
| BEV_ONA vs GAL_CCNU | - | - | 0.2 (-2.43, 2.96) | LOW^#&^ | 0.2 (-2.43, 2.96) | LOW |
| BEV_ONA vs REG | - | - | -0.41 (-2.81, 2.16) | LOW^#&^ | -0.41 (-2.81, 2.16) | LOW |
| BEV_ONA vs RIN_BEV | - | - | -0.29 (-2.26, 1.71) | LOW^%&^ | -0.29 (-2.26, 1.71) | LOW |
| BEV_TMZ vs CCNU | - | - | 0.14 (-2.06, 2.52) | LOW^#&^ | 0.14 (-2.06, 2.52) | LOW |
| BEV_TMZ vs CED | - | - | 0.55 (-2.04, 3.37) | LOW^#&^ | 0.55 (-2.04, 3.37) | LOW |
| BEV_TMZ vs CED_CCNU | - | - | -0.13 (-2.73, 2.64) | LOW^#&^ | -0.13 (-2.73, 2.64) | LOW |
| BEV_TMZ vs CED_GEF | - | - | 0.81 (-2.39, 4.24) | LOW^#&^ | 0.81 (-2.39, 4.24) | LOW |
| BEV_TMZ vs ENZ | - | - | 0.64 (-1.95, 3.44) | LOW^#&^ | 0.64 (-1.95, 3.44) | LOW |
| BEV_TMZ vs FOT | - | - | 1.29 (-1.13, 3.9) | LOW^#&^ | 1.29 (-1.13, 3.9) | LOW |
| BEV_TMZ vs GAL | - | - | -0.71 (-3.63, 2.29) | LOW^#&^ | -0.71 (-3.63, 2.29) | LOW |
| BEV_TMZ vs GAL_CCNU | - | - | 0.19 (-2.74, 3.19) | LOW^#&^ | 0.19 (-2.74, 3.19) | LOW |
| BEV_TMZ vs REG | - | - | -0.44 (-3.11, 2.41) | LOW^#&^ | -0.44 (-3.11, 2.41) | LOW |
| BEV_TMZ vs RIN_BEV | - | - | -0.3 (-2.67, 2.05) | VERY LOW^#%&^ | -0.3 (-2.67, 2.05) | VERY LOW |
| CCNU vs CED | 0.42 (-0.99, 1.83) | LOW^#&^ | - | - | 0.42 (-0.99, 1.83) | LOW |
| CCNU vs CED_CCNU | -0.26 (-1.65, 1.12) | LOW^#&^ | - | - | -0.26 (-1.65, 1.12) | LOW |
| CCNU vs CED_GEF | - | - | 0.67 (-1.64, 3.07) | LOW^#&^ | 0.67 (-1.64, 3.07) | LOW |
| CCNU vs ENZ | 0.5 (-0.89, 1.9) | LOW^#&^ | - | - | 0.5 (-0.89, 1.9) | LOW |
| CCNU vs FOT | - | - | 1.15 (-1.02, 3.37) | LOW^#&^ | 1.15 (-1.02, 3.37) | LOW |
| CCNU vs GAL | -0.83 (-2.82, 0.97) | MODERATE^&^ | - | - | -0.83 (-2.82, 0.97) | MODERATE |
| CCNU vs GAL_CCNU | 0.07 (-1.97, 1.88) | MODERATE^&^ | - | - | 0.07 (-1.97, 1.88) | MODERATE |
| CCNU vs REG | -0.56 (-2.16, 0.96) | MODERATE^&^ | - | - | -0.56 (-2.16, 0.96) | MODERATE |
| CCNU vs RIN_BEV | - | - | -0.45 (-2.56, 1.5) | VERY LOW^#%&^ | -0.45 (-2.56, 1.5) | VERY LOW |
| CED vs CED_CCNU | -0.68 (-2.08, 0.69) | LOW^#&^ | - | - | -0.68 (-2.08, 0.69) | LOW |
| CED vs CED_GEF | 0.24 (-1.6, 2.2) | LOW^#&^ | - | - | 0.24 (-1.6, 2.2) | LOW |
| CED vs ENZ | - | - | 0.07 (-1.89, 2.08) | LOW^#&^ | 0.07 (-1.89, 2.08) | LOW |
| CED vs FOT | - | - | 0.73 (-1.87, 3.38) | LOW^#&^ | 0.73 (-1.87, 3.38) | LOW |
| CED vs GAL | - | - | -1.27 (-3.68, 1.02) | LOW^#&^ | -1.27 (-3.68, 1.02) | LOW |
| CED vs GAL_CCNU | - | - | -0.38 (-2.81, 1.94) | LOW^#&^ | -0.38 (-2.81, 1.94) | LOW |
| CED vs REG | - | - | -0.99 (-3.1, 1.08) | LOW^#&^ | -0.99 (-3.1, 1.08) | LOW |
| CED vs RIN_BEV | - | - | -0.87 (-3.45, 1.52) | VERY LOW^#%&^ | -0.87 (-3.45, 1.52) | VERY LOW |
| CED_CCNU vs CED_GEF | - | - | 0.93 (-1.35, 3.33) | LOW^#&^ | 0.93 (-1.35, 3.33) | LOW |
| CED_CCNU vs ENZ | - | - | 0.76 (-1.2, 2.71) | LOW^#&^ | 0.76 (-1.2, 2.71) | LOW |
| CED_CCNU vs FOT | - | - | 1.42 (-1.18, 4.04) | LOW^#&^ | 1.42 (-1.18, 4.04) | LOW |
| CED_CCNU vs GAL | - | - | -0.59 (-2.97, 1.68) | LOW^#&^ | -0.59 (-2.97, 1.68) | LOW |
| CED_CCNU vs GAL_CCNU | - | - | 0.31 (-2.12, 2.6) | LOW^#&^ | 0.31 (-2.12, 2.6) | LOW |
| CED_CCNU vs REG | - | - | -0.31 (-2.4, 1.74) | LOW^#&^ | -0.31 (-2.4, 1.74) | LOW |
| CED_CCNU vs RIN_BEV | - | - | -0.18 (-2.75, 2.2) | VERY LOW^#%&^ | -0.18 (-2.75, 2.2) | VERY LOW |
| CED_GEF vs ENZ | - | - | -0.16 (-2.95, 2.51) | LOW^#&^ | -0.16 (-2.95, 2.51) | LOW |
| CED_GEF vs FOT | - | - | 0.49 (-2.8, 3.71) | LOW^#&^ | 0.49 (-2.8, 3.71) | LOW |
| CED_GEF vs GAL | - | - | -1.55 (-4.62, 1.45) | LOW^#&^ | -1.55 (-4.62, 1.45) | LOW |
| CED_GEF vs GAL_CCNU | - | - | -0.65 (-3.77, 2.38) | LOW^#&^ | -0.65 (-3.77, 2.38) | LOW |
| CED_GEF vs REG | - | - | -1.24 (-4.12, 1.5) | LOW^#&^ | -1.24 (-4.12, 1.5) | LOW |
| CED_GEF vs RIN_BEV | - | - | -1.13 (-4.36, 1.9) | VERY LOW^#%&^ | -1.13 (-4.36, 1.9) | VERY LOW |
| ENZ vs FOT | - | - | 0.66 (-1.9, 3.26) | LOW^#&^ | 0.66 (-1.9, 3.26) | LOW |
| ENZ vs GAL | - | - | -1.34 (-3.76, 0.91) | LOW^#&^ | -1.34 (-3.76, 0.91) | LOW |
| ENZ vs GAL_CCNU | - | - | -0.44 (-2.88, 1.84) | LOW^#&^ | -0.44 (-2.88, 1.84) | LOW |
| ENZ vs REG | - | - | -1.08 (-3.18, 0.97) | LOW^#&^ | -1.08 (-3.18, 0.97) | LOW |
| ENZ vs RIN_BEV | - | - | -0.95 (-3.48, 1.4) | VERY LOW^#%&^ | -0.95 (-3.48, 1.4) | VERY LOW |
| FOT vs GAL | - | - | -2.02 (-4.92, 0.83) | LOW^#&^ | -2.02 (-4.92, 0.83) | LOW |
| FOT vs GAL_CCNU | - | - | -1.14 (-4.04, 1.75) | LOW^#&^ | -1.14 (-4.04, 1.75) | LOW |
| FOT vs REG | - | - | -1.74 (-4.45, 0.93) | LOW^#&^ | -1.74 (-4.45, 0.93) | LOW |
| FOT vs RIN_BEV | - | - | -1.6 (-3.96, 0.54) | LOW^%&^ | -1.6 (-3.96, 0.54) | LOW |
| GAL vs GAL_CCNU | - | - | 0.9 (-0.76, 2.57) | LOW^#&^ | 0.9 (-0.76, 2.57) | LOW |
| GAL vs REG | - | - | 0.25 (-2.1, 2.76) | LOW^#&^ | 0.25 (-2.1, 2.76) | LOW |
| GAL vs RIN_BEV | - | - | 0.4 (-2.38, 3.13) | VERY LOW^#%&^ | 0.4 (-2.38, 3.13) | VERY LOW |
| GAL_CCNU vs REG | - | - | -0.62 (-3.04, 1.89) | MODERATE^&^ | -0.62 (-3.04, 1.89) | MODERATE |
| GAL_CCNU vs RIN_BEV | - | - | -0.49 (-3.3, 2.25) | VERY LOW^#%&^ | -0.49 (-3.3, 2.25) | VERY LOW |
| REG vs RIN | - | - | 0.13 (-2.51, 2.59) | VERY LOW^#%&^ | 0.13 (-2.51, 2.59) | VERY LOW |
| # limitation, $ inconsistency, % indirectness, & publication bias | | | | | | |

Table S 6 GRADE valuation of evidence of 6m PFS rate

c. OS

|  | Direct evidence | | Indirect evidence | | Network meta-analysis | |
| --- | --- | --- | --- | --- | --- | --- |
| Comparison | Risk ratio (95% credible interval) | Quality of evidence | Risk ratio (95% credible interval) | Quality of evidence | Risk ratio (95% credible interval) | Quality of evidence |
| BEV vs BEV5_CCNU90 | 0.16 (-0.85, 1.19) | LOW^#&^ | - | - | 0.16 (-0.85, 1.19) | LOW |
| BEV vs BEV_CAR | -0.16 (-1.08, 0.74) | LOW^#&^ | - | - | -0.16 (-1.08, 0.74) | LOW |
| BEV vs BEV_CCNU | 0.43 (-0.43, 1.44) | LOW^#&^ | - | - | 0.43 (-0.43, 1.44) | LOW |
| BEV vs BEV_IRI | -0.12 (-1.05, 0.81) | LOW^#&^ | - | - | -0.12 (-1.05, 0.81) | LOW |
| BEV vs BEV_ONA | -0.37 (-1.33, 0.59) | MODERATE^&^ | - | - | -0.37 (-1.33, 0.59) | MODERATE |
| BEV vs BEV_TMZ | - | - | -0.03 (-1.37, 1.3) | LOW^#&^ | -0.03 (-1.37, 1.3) | LOW |
| BEV vs CCNU | 0.2 (-0.68, 1.11) | LOW^#&^ | - | - | 0.2 (-0.68, 1.11) | LOW |
| BEV vs CED | - | - | -0.16 (-1.43, 1.13) | LOW^#&^ | -0.16 (-1.43, 1.13) | LOW |
| BEV vs CED_CCNU | - | - | 0.06 (-1.22, 1.34) | LOW^#&^ | 0.06 (-1.22, 1.34) | LOW |
| BEV vs CED_GEF | - | - | 0.22 (-1.36, 1.85) | LOW^#&^ | 0.22 (-1.36, 1.85) | LOW |
| BEV vs ENZ | - | - | 0.03 (-1.24, 1.3) | LOW^#&^ | 0.03 (-1.24, 1.3) | LOW |
| BEV vs FOT | 0.46 (-0.5, 1.4) | MODERATE^&^ | - | - | 0.46 (-0.5, 1.4) | MODERATE |
| BEV vs GAL | - | - | 0.27 (-1.03, 1.59) | LOW^#&^ | 0.27 (-1.03, 1.59) | LOW |
| BEV vs GAL_CCNU | - | - | 0.08 (-1.18, 1.37) | LOW^#&^ | 0.08 (-1.18, 1.37) | LOW |
| BEV vs REG | - | - | 0.69 (-0.24, 1.61) | LOW^#&^ | 0.69 (-0.24, 1.61) | LOW |
| BEV vs RIN_BEV | 0.84 (-0.48, 2.17) | LOW^%&^ | - | - | 0.84 (-0.48, 2.17) | LOW |
| BEV vs TTF_BEV | 0.49 (-0.47, 1.44) | MODERATE^&^ | - | - | 0.49 (-0.47, 1.44) | MODERATE |
| BEV5_CCNU90 vs BEV_CAR | - | - | 0.33 (-1.01, 1.7) | LOW^#&^ | 0.33 (-1.01, 1.7) | LOW |
| BEV5_CCNU90 vs BEV_CCNU | - | - | -0.27 (-1.72, 1.06) | LOW^#&^ | -0.27 (-1.72, 1.06) | LOW |
| BEV5_CCNU90 vs BEV_IRI | - | - | 0.29 (-1.08, 1.66) | LOW^#&^ | 0.29 (-1.08, 1.66) | LOW |
| BEV5_CCNU90 vs BEV_ONA | - | - | 0.53 (-0.85, 1.93) | LOW^#&^ | 0.53 (-0.85, 1.93) | LOW |
| BEV5_CCNU90 vs BEV_TMZ | - | - | 0.2 (-1.46, 1.88) | LOW^#&^ | 0.2 (-1.46, 1.88) | LOW |
| BEV5_CCNU90 vs CCNU | - | - | 0.04 (-1.32, 1.43) | LOW^#&^ | 0.04 (-1.32, 1.43) | LOW |
| BEV5_CCNU90 vs CED | - | - | -0.33 (-1.97, 1.33) | LOW^#&^ | -0.33 (-1.97, 1.33) | LOW |
| BEV5_CCNU90 vs CED_CCNU | - | - | -0.11 (-1.76, 1.56) | LOW^#&^ | -0.11 (-1.76, 1.56) | LOW |
| BEV5_CCNU90 vs CED_GEF | - | - | 0.06 (-1.84, 2) | LOW^#&^ | 0.06 (-1.84, 2) | LOW |
| BEV5_CCNU90 vs ENZ | - | - | -0.14 (-1.76, 1.51) | LOW^#&^ | -0.14 (-1.76, 1.51) | LOW |
| BEV5_CCNU90 vs FOT | - | - | 0.29 (-1.1, 1.68) | LOW^#&^ | 0.29 (-1.1, 1.68) | LOW |
| BEV5_CCNU90 vs GAL | - | - | 0.1 (-1.54, 1.78) | LOW^#&^ | 0.1 (-1.54, 1.78) | LOW |
| BEV5_CCNU90 vs GAL_CCNU | - | - | -0.09 (-1.71, 1.56) | LOW^#&^ | -0.09 (-1.71, 1.56) | LOW |
| BEV5_CCNU90 vs REG | - | - | 0.52 (-0.87, 1.9) | LOW^#&^ | 0.52 (-0.87, 1.9) | LOW |
| BEV5_CCNU90 vs RIN_BEV | - | - | 0.67 (-1, 2.36) | VERY LOW^#%&^ | 0.67 (-1, 2.36) | VERY LOW |
| BEV5_CCNU90 vs TTF_BEV | - | - | 0.33 (-1.09, 1.73) | LOW^#&^ | 0.33 (-1.09, 1.73) | LOW |
| BEV_CAR vs BEV_CCNU | - | - | 0.6 (-0.63, 1.98) | LOW^#&^ | 0.6 (-0.63, 1.98) | LOW |
| BEV_CAR vs BEV_IRI | - | - | 0.05 (-1.25, 1.33) | LOW^#&^ | 0.05 (-1.25, 1.33) | LOW |
| BEV_CAR vs BEV_ONA | - | - | -0.2 (-1.52, 1.11) | LOW^#&^ | -0.2 (-1.52, 1.11) | LOW |
| BEV_CAR vs BEV_TMZ | - | - | 0.14 (-1.48, 1.73) | LOW^#&^ | 0.14 (-1.48, 1.73) | LOW |
| BEV_CAR vs CCNU | - | - | 0.37 (-0.89, 1.66) | LOW^#&^ | 0.37 (-0.89, 1.66) | LOW |
| BEV_CAR vs CED | - | - | 0.01 (-1.56, 1.59) | LOW^#&^ | 0.01 (-1.56, 1.59) | LOW |
| BEV_CAR vs CED_CCNU | - | - | 0.23 (-1.35, 1.81) | LOW^#&^ | 0.23 (-1.35, 1.81) | LOW |
| BEV_CAR vs CED_GEF | - | - | 0.39 (-1.42, 2.27) | LOW^#&^ | 0.39 (-1.42, 2.27) | LOW |
| BEV_CAR vs ENZ | - | - | 0.19 (-1.36, 1.77) | LOW^#&^ | 0.19 (-1.36, 1.77) | LOW |
| BEV_CAR vs FOT | - | - | 0.63 (-0.68, 1.93) | LOW^#&^ | 0.63 (-0.68, 1.93) | LOW |
| BEV_CAR vs GAL | - | - | 0.43 (-1.13, 2.03) | LOW^#&^ | 0.43 (-1.13, 2.03) | LOW |
| BEV_CAR vs GAL_CCNU | - | - | 0.25 (-1.29, 1.84) | LOW^#&^ | 0.25 (-1.29, 1.84) | LOW |
| BEV_CAR vs REG | - | - | 0.86 (-0.45, 2.15) | LOW^#&^ | 0.86 (-0.45, 2.15) | LOW |
| BEV_CAR vs RIN_BEV | - | - | 1.01 (-0.6, 2.62) | VERY LOW^#%&^ | 1.01 (-0.6, 2.62) | VERY LOW |
| BEV_CAR vs TTF_BEV | - | - | 0.65 (-0.67, 1.97) | LOW^#&^ | 0.65 (-0.67, 1.97) | LOW |
| BEV_CCNU vs BEV_IRI | - | - | -0.55 (-1.94, 0.69) | LOW^#&^ | -0.55 (-1.94, 0.69) | LOW |
| BEV_CCNU vs BEV_ONA | - | - | -0.8 (-2.21, 0.47) | LOW^#&^ | -0.8 (-2.21, 0.47) | LOW |
| BEV_CCNU vs BEV_TMZ | - | - | -0.46 (-2.18, 1.09) | LOW^#&^ | -0.46 (-2.18, 1.09) | LOW |
| BEV_CCNU vs CCNU | -0.22 (-0.95, 0.37) | MODERATE^&^ | - | - | -0.22 (-0.95, 0.37) | MODERATE |
| BEV_CCNU vs CED | - | - | -0.59 (-1.78, 0.46) | LOW^#&^ | -0.59 (-1.78, 0.46) | LOW |
| BEV_CCNU vs CED_CCNU | - | - | -0.38 (-1.58, 0.69) | LOW^#&^ | -0.38 (-1.58, 0.69) | LOW |
| BEV_CCNU vs CED_GEF | - | - | -0.2 (-1.76, 1.22) | LOW^#&^ | -0.2 (-1.76, 1.22) | LOW |
| BEV_CCNU vs ENZ | - | - | -0.4 (-1.58, 0.63) | LOW^#&^ | -0.4 (-1.58, 0.63) | LOW |
| BEV_CCNU vs FOT | - | - | 0.03 (-1.39, 1.29) | LOW^#&^ | 0.03 (-1.39, 1.29) | LOW |
| BEV_CCNU vs GAL | - | - | -0.17 (-1.38, 0.93) | LOW^#&^ | -0.17 (-1.38, 0.93) | LOW |
| BEV_CCNU vs GAL_CCNU | - | - | -0.35 (-1.53, 0.71) | LOW^#&^ | -0.35 (-1.53, 0.71) | LOW |
| BEV_CCNU vs REG | - | - | 0.26 (-1.14, 1.49) | LOW^#&^ | 0.26 (-1.14, 1.49) | LOW |
| BEV_CCNU vs RIN_BEV | - | - | 0.4 (-0.83, 1.5) | VERY LOW^#%&^ | 0.4 (-0.83, 1.5) | VERY LOW |
| BEV_CCNU vs TTF_BEV | - | - | 0.04 (-1.37, 1.33) | LOW^#&^ | 0.04 (-1.37, 1.33) | LOW |
| BEV_IRI vs BEV_ONA | - | - | -0.25 (-1.57, 1.07) | LOW^#&^ | -0.25 (-1.57, 1.07) | LOW |
| BEV_IRI vs BEV_TMZ | 0.09 (-0.87, 1.04) | MODERATE^&^ | - | - | 0.09 (-0.87, 1.04) | MODERATE |
| BEV_IRI vs CCNU | - | - | 0.32 (-0.95, 1.63) | LOW^#&^ | 0.32 (-0.95, 1.63) | LOW |
| BEV_IRI vs CED | - | - | -0.04 (-1.61, 1.54) | LOW^#&^ | -0.04 (-1.61, 1.54) | LOW |
| BEV_IRI vs CED_CCNU | - | - | 0.18 (-1.39, 1.77) | LOW^#&^ | 0.18 (-1.39, 1.77) | LOW |
| BEV_IRI vs CED_GEF | - | - | 0.34 (-1.47, 2.23) | LOW^#&^ | 0.34 (-1.47, 2.23) | LOW |
| BEV_IRI vs ENZ | - | - | 0.15 (-1.42, 1.71) | LOW^#&^ | 0.15 (-1.42, 1.71) | LOW |
| BEV_IRI vs FOT | - | - | 0.58 (-0.75, 1.89) | LOW^#&^ | 0.58 (-0.75, 1.89) | LOW |
| BEV_IRI vs GAL | - | - | 0.38 (-1.2, 2.01) | LOW^#&^ | 0.38 (-1.2, 2.01) | LOW |
| BEV_IRI vs GAL_CCNU | - | - | 0.2 (-1.35, 1.79) | LOW^#&^ | 0.2 (-1.35, 1.79) | LOW |
| BEV_IRI vs REG | - | - | 0.8 (-0.49, 2.11) | LOW^#&^ | 0.8 (-0.49, 2.11) | LOW |
| BEV_IRI vs RIN_BEV | - | - | 0.95 (-0.65, 2.58) | VERY LOW^#%&^ | 0.95 (-0.65, 2.58) | VERY LOW |
| BEV_IRI vs TTF_BEV | - | - | 0.6 (-0.71, 1.93) | LOW^#&^ | 0.6 (-0.71, 1.93) | LOW |
| BEV_ONA vs BEV_TMZ | - | - | 0.34 (-1.29, 1.95) | LOW^#&^ | 0.34 (-1.29, 1.95) | LOW |
| BEV_ONA vs CCNU | - | - | 0.57 (-0.72, 1.9) | LOW^#&^ | 0.57 (-0.72, 1.9) | LOW |
| BEV_ONA vs CED | - | - | 0.21 (-1.38, 1.81) | LOW^#&^ | 0.21 (-1.38, 1.81) | LOW |
| BEV_ONA vs CED_CCNU | - | - | 0.43 (-1.17, 2.05) | LOW^#&^ | 0.43 (-1.17, 2.05) | LOW |
| BEV_ONA vs CED_GEF | - | - | 0.6 (-1.26, 2.48) | LOW^#&^ | 0.6 (-1.26, 2.48) | LOW |
| BEV_ONA vs ENZ | - | - | 0.4 (-1.21, 1.99) | LOW^#&^ | 0.4 (-1.21, 1.99) | LOW |
| BEV_ONA vs FOT | - | - | 0.84 (-0.53, 2.17) | MODERATE^&^ | 0.84 (-0.53, 2.17) | MODERATE |
| BEV_ONA vs GAL | - | - | 0.64 (-0.98, 2.29) | LOW^#&^ | 0.64 (-0.98, 2.29) | LOW |
| BEV_ONA vs GAL_CCNU | - | - | 0.45 (-1.13, 2.06) | LOW^#&^ | 0.45 (-1.13, 2.06) | LOW |
| BEV_ONA vs REG | - | - | 1.05 (-0.27, 2.38) | LOW^#&^ | 1.05 (-0.27, 2.38) | LOW |
| BEV_ONA vs RIN_BEV | - | - | 1.2 (-0.41, 2.84) | LOW^%&^ | 1.2 (-0.41, 2.84) | LOW |
| BEV_ONA vs TTF_BEV | - | - | 0.86 (-0.49, 2.21) | MODERATE^&^ | 0.86 (-0.49, 2.21) | MODERATE |
| BEV_TMZ vs CCNU | - | - | 0.23 (-1.37, 1.86) | LOW^#&^ | 0.23 (-1.37, 1.86) | LOW |
| BEV_TMZ vs CED | - | - | -0.13 (-1.96, 1.72) | LOW^#&^ | -0.13 (-1.96, 1.72) | LOW |
| BEV_TMZ vs CED_CCNU | - | - | 0.09 (-1.74, 1.95) | LOW^#&^ | 0.09 (-1.74, 1.95) | LOW |
| BEV_TMZ vs CED_GEF | - | - | 0.25 (-1.8, 2.36) | LOW^#&^ | 0.25 (-1.8, 2.36) | LOW |
| BEV_TMZ vs ENZ | - | - | 0.05 (-1.78, 1.91) | LOW^#&^ | 0.05 (-1.78, 1.91) | LOW |
| BEV_TMZ vs FOT | - | - | 0.49 (-1.14, 2.12) | LOW^#&^ | 0.49 (-1.14, 2.12) | LOW |
| BEV_TMZ vs GAL | - | - | 0.3 (-1.54, 2.2) | LOW^#&^ | 0.3 (-1.54, 2.2) | LOW |
| BEV_TMZ vs GAL_CCNU | - | - | 0.11 (-1.71, 1.97) | LOW^#&^ | 0.11 (-1.71, 1.97) | LOW |
| BEV_TMZ vs REG | - | - | 0.71 (-0.88, 2.35) | LOW^#&^ | 0.71 (-0.88, 2.35) | LOW |
| BEV_TMZ vs RIN_BEV | - | - | 0.87 (-0.99, 2.75) | VERY LOW^#%&^ | 0.87 (-0.99, 2.75) | VERY LOW |
| BEV_TMZ vs TTF_BEV | - | - | 0.52 (-1.11, 2.16) | LOW^#&^ | 0.52 (-1.11, 2.16) | LOW |
| CCNU vs CED | -0.36 (-1.28, 0.54) | LOW^#&^ | - | - | -0.36 (-1.28, 0.54) | LOW |
| CCNU vs CED_CCNU | -0.15 (-1.06, 0.78) | LOW^#&^ | - | - | -0.15 (-1.06, 0.78) | LOW |
| CCNU vs CED_GEF | - | - | 0.03 (-1.3, 1.36) | LOW^#&^ | 0.03 (-1.3, 1.36) | LOW |
| CCNU vs ENZ | -0.18 (-1.07, 0.72) | LOW^#&^ | - | - | -0.18 (-1.07, 0.72) | LOW |
| CCNU vs FOT | - | - | 0.26 (-1.07, 1.55) | LOW^#&^ | 0.26 (-1.07, 1.55) | LOW |
| CCNU vs GAL | 0.07 (-0.87, 1.02) | MODERATE^&^ | - | - | 0.07 (-0.87, 1.02) | MODERATE |
| CCNU vs GAL_CCNU | -0.12 (-1.02, 0.79) | MODERATE^&^ | - | - | -0.12 (-1.02, 0.79) | MODERATE |
| CCNU vs REG | 0.49 (-0.82, 1.76) | MODERATE^&^ | - | - | 0.49 (-0.82, 1.76) | MODERATE |
| CCNU vs RIN_BEV | - | - | 0.63 (-0.33, 1.6) | VERY LOW^#%&^ | 0.63 (-0.33, 1.6) | VERY LOW |
| CCNU vs TTF_BEV | - | - | 0.28 (-1.04, 1.57) | LOW^#&^ | 0.28 (-1.04, 1.57) | LOW |
| CED vs CED_CCNU | 0.21 (-0.68, 1.11) | LOW^#&^ | - | - | 0.21 (-0.68, 1.11) | LOW |
| CED vs CED_GEF | 0.39 (-0.59, 1.37) | LOW^#&^ | - | - | 0.39 (-0.59, 1.37) | LOW |
| CED vs ENZ | - | - | 0.18 (-1.09, 1.47) | LOW^#&^ | 0.18 (-1.09, 1.47) | LOW |
| CED vs FOT | - | - | 0.62 (-0.99, 2.2) | LOW^#&^ | 0.62 (-0.99, 2.2) | LOW |
| CED vs GAL | - | - | 0.43 (-0.89, 1.76) | LOW^#&^ | 0.43 (-0.89, 1.76) | LOW |
| CED vs GAL_CCNU | - | - | 0.24 (-1.04, 1.53) | LOW^#&^ | 0.24 (-1.04, 1.53) | LOW |
| CED vs REG | - | - | 0.85 (-0.74, 2.4) | LOW^#&^ | 0.85 (-0.74, 2.4) | LOW |
| CED vs RIN_BEV | - | - | 1 (-0.33, 2.32) | VERY LOW^#%&^ | 1 (-0.33, 2.32) | VERY LOW |
| CED vs TTF_BEV | - | - | 0.64 (-0.95, 2.22) | LOW^#&^ | 0.64 (-0.95, 2.22) | LOW |
| CED_CCNU vs CED_GEF | - | - | 0.18 (-1.15, 1.51) | LOW^#&^ | 0.18 (-1.15, 1.51) | LOW |
| CED_CCNU vs ENZ | - | - | -0.03 (-1.32, 1.24) | LOW^#&^ | -0.03 (-1.32, 1.24) | LOW |
| CED_CCNU vs FOT | - | - | 0.4 (-1.22, 1.99) | LOW^#&^ | 0.4 (-1.22, 1.99) | LOW |
| CED_CCNU vs GAL | - | - | 0.21 (-1.1, 1.54) | LOW^#&^ | 0.21 (-1.1, 1.54) | LOW |
| CED_CCNU vs GAL_CCNU | - | - | 0.03 (-1.27, 1.31) | LOW^#&^ | 0.03 (-1.27, 1.31) | LOW |
| CED_CCNU vs REG | - | - | 0.64 (-0.95, 2.18) | LOW^#&^ | 0.64 (-0.95, 2.18) | LOW |
| CED_CCNU vs RIN_BEV | - | - | 0.78 (-0.56, 2.1) | VERY LOW^#%&^ | 0.78 (-0.56, 2.1) | VERY LOW |
| CED_CCNU vs TTF_BEV | - | - | 0.43 (-1.17, 2.03) | LOW^#&^ | 0.43 (-1.17, 2.03) | LOW |
| CED_GEF vs ENZ | - | - | -0.21 (-1.8, 1.41) | LOW^#&^ | -0.21 (-1.8, 1.41) | LOW |
| CED_GEF vs FOT | - | - | 0.24 (-1.67, 2.08) | LOW^#&^ | 0.24 (-1.67, 2.08) | LOW |
| CED_GEF vs GAL | - | - | 0.04 (-1.6, 1.69) | LOW^#&^ | 0.04 (-1.6, 1.69) | LOW |
| CED_GEF vs GAL_CCNU | - | - | -0.14 (-1.76, 1.47) | LOW^#&^ | -0.14 (-1.76, 1.47) | LOW |
| CED_GEF vs REG | - | - | 0.47 (-1.41, 2.29) | LOW^#&^ | 0.47 (-1.41, 2.29) | LOW |
| CED_GEF vs RIN_BEV | - | - | 0.6 (-1.04, 2.25) | LOW^%&^ | 0.6 (-1.04, 2.25) | LOW |
| CED_GEF vs TTF_BEV | - | - | 0.26 (-1.61, 2.1) | LOW^#&^ | 0.26 (-1.61, 2.1) | LOW |
| ENZ vs FOT | - | - | 0.44 (-1.19, 2.01) | LOW^#&^ | 0.44 (-1.19, 2.01) | LOW |
| ENZ vs GAL | - | - | 0.24 (-1.06, 1.55) | LOW^#&^ | 0.24 (-1.06, 1.55) | LOW |
| ENZ vs GAL_CCNU | - | - | 0.06 (-1.22, 1.34) | LOW^#&^ | 0.06 (-1.22, 1.34) | LOW |
| ENZ vs REG | - | - | 0.67 (-0.92, 2.23) | LOW^#&^ | 0.67 (-0.92, 2.23) | LOW |
| ENZ vs RIN_BEV | - | - | 0.81 (-0.51, 2.11) | VERY LOW^#%&^ | 0.81 (-0.51, 2.11) | VERY LOW |
| ENZ vs TTF_BEV | - | - | 0.46 (-1.14, 2.04) | LOW^#&^ | 0.46 (-1.14, 2.04) | LOW |
| FOT vs GAL | - | - | -0.2 (-1.81, 1.45) | LOW^#&^ | -0.2 (-1.81, 1.45) | LOW |
| FOT vs GAL_CCNU | - | - | -0.38 (-1.97, 1.22) | LOW^#&^ | -0.38 (-1.97, 1.22) | LOW |
| FOT vs REG | - | - | 0.23 (-1.08, 1.55) | LOW^#&^ | 0.23 (-1.08, 1.55) | LOW |
| FOT vs RIN_BEV | - | - | 0.37 (-1.24, 2.05) | LOW^%&^ | 0.37 (-1.24, 2.05) | LOW |
| FOT vs TTF_BEV | - | - | 0.03 (-1.32, 1.38) | LOW^#&^ | 0.03 (-1.32, 1.38) | LOW |
| GAL vs GAL_CCNU | - | - | -0.19 (-1.13, 0.75) | LOW^#&^ | -0.19 (-1.13, 0.75) | LOW |
| GAL vs REG | - | - | 0.42 (-1.2, 2) | LOW^#&^ | 0.42 (-1.2, 2) | LOW |
| GAL vs RIN_BEV | - | - | 0.57 (-0.78, 1.9) | VERY LOW^#%&^ | 0.57 (-0.78, 1.9) | VERY LOW |
| GAL vs TTF_BEV | - | - | 0.21 (-1.42, 1.82) | MODERATE^&^ | 0.21 (-1.42, 1.82) | MODERATE |
| GAL_CCNU vs REG | - | - | 0.61 (-0.98, 2.18) | MODERATE^&^ | 0.61 (-0.98, 2.18) | MODERATE |
| GAL_CCNU vs RIN_BEV | - | - | 0.75 (-0.57, 2.06) | VERY LOW^#%&^ | 0.75 (-0.57, 2.06) | VERY LOW |
| GAL_CCNU vs TTF_BEV | - | - | 0.4 (-1.2, 1.99) | LOW^#&^ | 0.4 (-1.2, 1.99) | LOW |
| REG vs RIN_BEV | - | - | 0.14 (-1.46, 1.78) | VERY LOW^#%&^ | 0.14 (-1.46, 1.78) | VERY LOW |
| REG vs TTF_BEV | - | - | -0.2 (-1.54, 1.13) | LOW^#&^ | -0.2 (-1.54, 1.13) | LOW |
| RIN_BEV vs TTF_BEV | - | - | -0.35 (-1.98, 1.27) | LOW^#&^ | -0.35 (-1.98, 1.27) | LOW |
| # limitation, $ inconsistency, % indirectness, & publication bias | | | | | | |

Table S 7 GRADE valuation of evidence of OS

**References**

Brignardello-Petersen, R., Bonner, A., Alexander, P.E., Siemieniuk, R.A., Furukawa, T.A., Rochwerg, B., et al. (2018). Advances in the GRADE approach to rate the certainty in estimates from a network meta-analysis. *J Clin Epidemiol* 93**,** 36-44. doi: 10.1016/j.jclinepi.2017.10.005.

Puhan, M.A., Schünemann, H.J., Murad, M.H., Li, T., Brignardello-Petersen, R., Singh, J.A., et al. (2014). A GRADE Working Group approach for rating the quality of treatment effect estimates from network meta-analysis. *Bmj* 349**,** g5630. doi: 10.1136/bmj.g5630.

Trelle, S., Reichenbach, S., Wandel, S., Hildebrand, P., Tschannen, B., Villiger, P.M., et al. (2011). Cardiovascular safety of non-steroidal anti-inflammatory drugs: network meta-analysis. *BMJ (Clinical research ed.)* 342**,** c7086-c7086. doi: 10.1136/bmj.c7086.

# 3. Supplementary results for objective response rate

1) Network plot


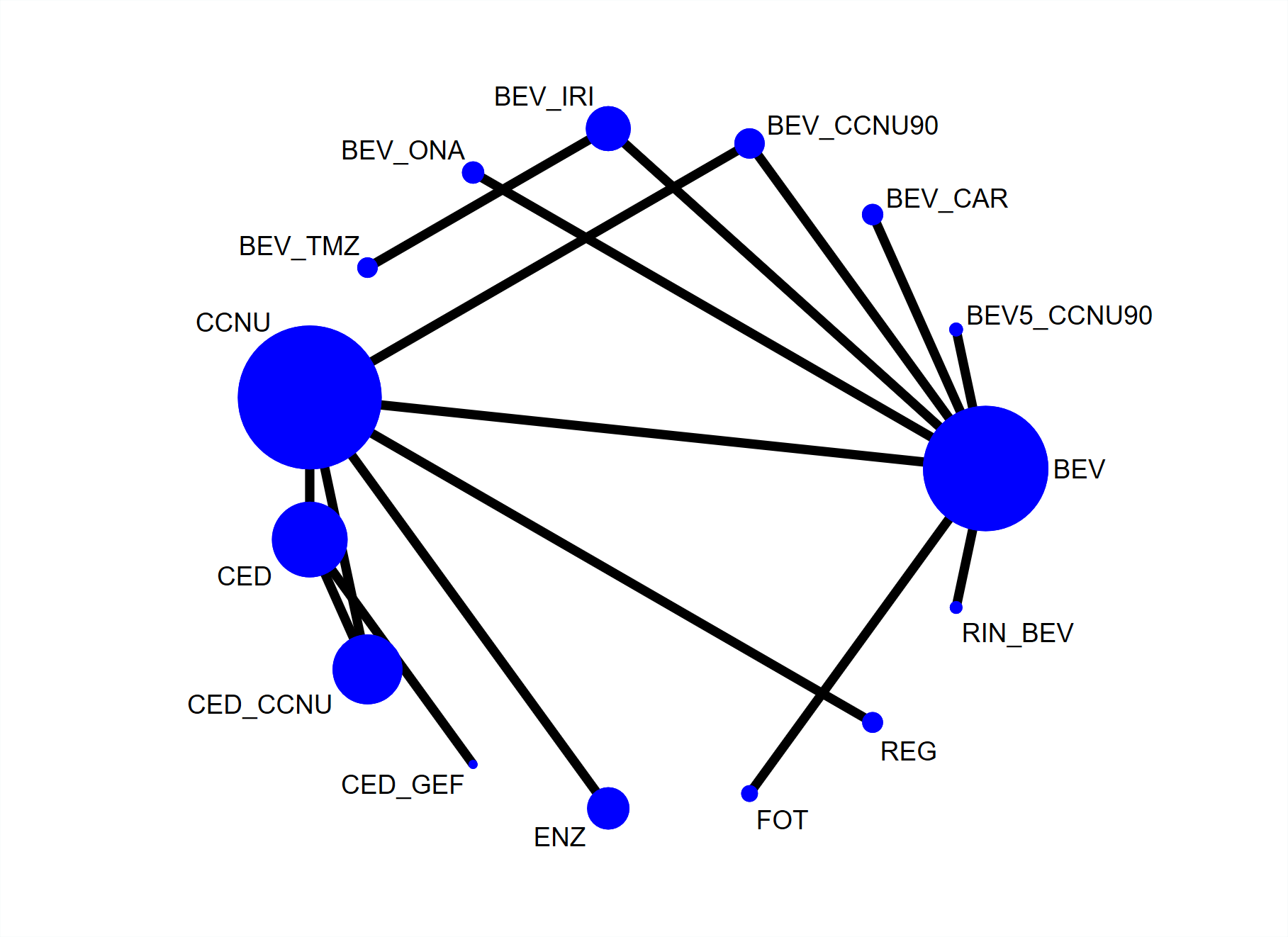


Figure S 1 Network plot of all comparisons involved in model of ORR

Size of every solid circle is proportional to the number of total sample size. Galunisertib monotherapy, Galunisertib plus CCNU and TTF combined with BEV were omitted due to missing data.

2) Publication bias


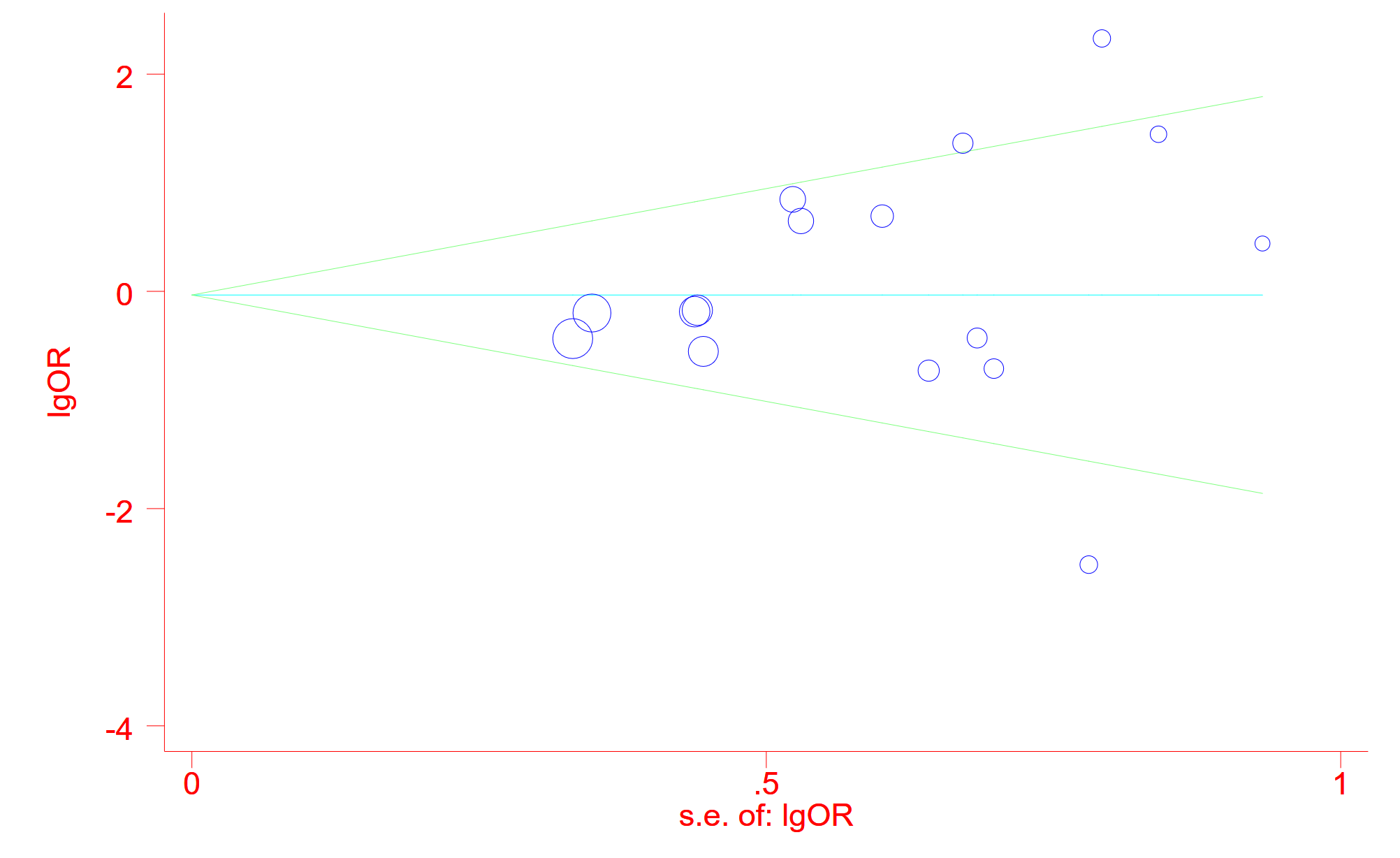


Begg's Test: Pr > |z| = 0.661

Egger's test: P > |t| = 0.579

3）Forest plot of relative effect compared with CCNU


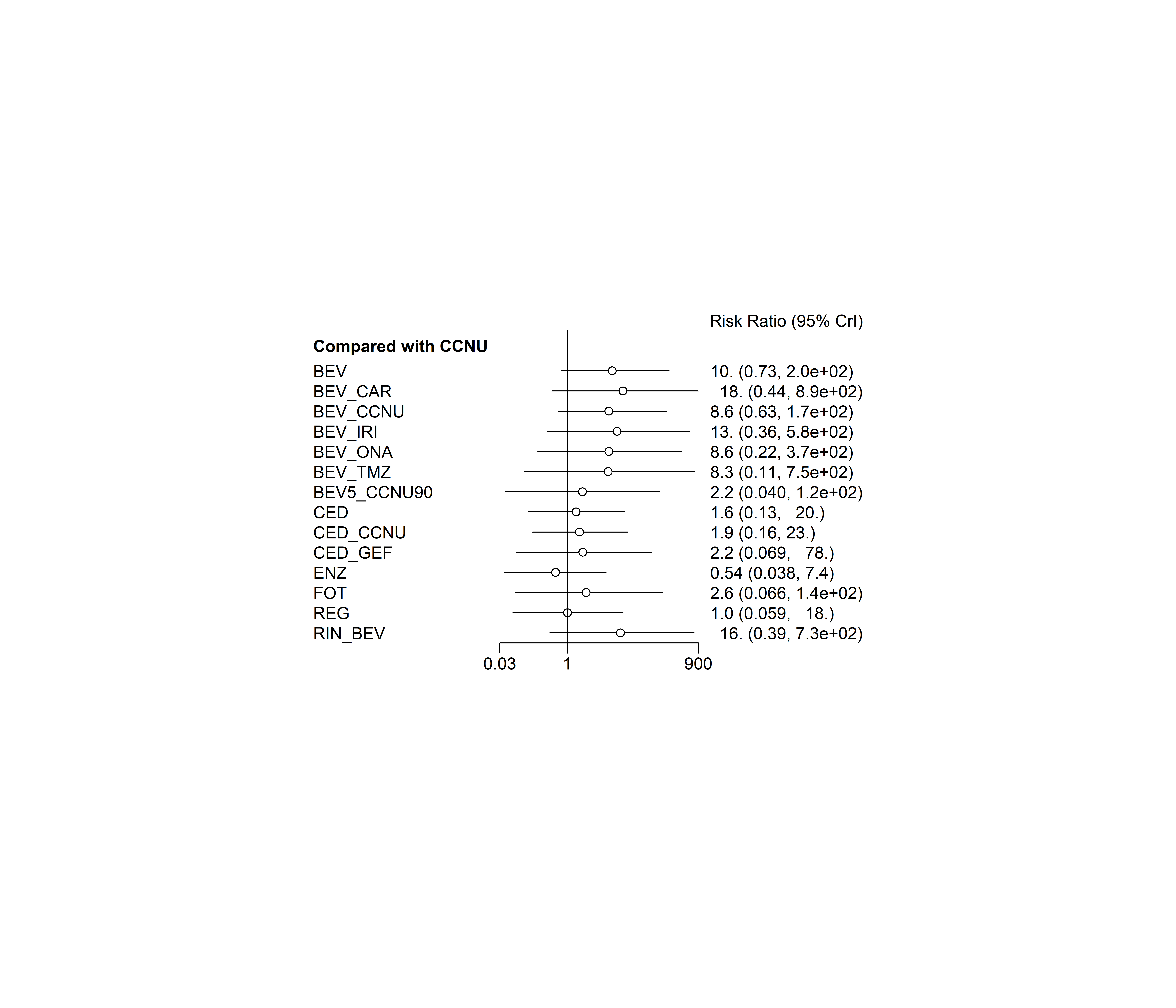


Figure S 2 Forest plot of relative effects compared with CCNU

All therapies were compared with CCNU about efficacy on ORR.

3) Head-to-head comparisons of all treatments

| **BEV** |  |  |  |  |  |  |  |  |  |  |  |  |  |  |
| --- | --- | --- | --- | --- | --- | --- | --- | --- | --- | --- | --- | --- | --- | --- |
| **-0.56**  **(-3.18, 2.03)** | **BEV_CAR** |  |  |  |  |  |  |  |  |  |  |  |  |  |
| **0.17**  **(-2.23, 2.64)** | **0.76**  **(-2.78, 4.32)** | **BEV_CCNU** |  |  |  |  |  |  |  |  |  |  |  |  |
| **-0.26**  **(-2.71, 2.14)** | **0.29**  **(-3.30, 3.82)** | **-0.44**  **(-3.92, 3)** | **BEV_IRI** |  |  |  |  |  |  |  |  |  |  |  |
| **0.2**  **(-2.26, 2.67)** | **0.76**  **(-2.78, 4.34)** | **0.02**  **(-3.44, 3.51)** | **0.47**  **(-2.98, 3.97)** | **BEV_ONA** |  |  |  |  |  |  |  |  |  |  |
| **0.22**  **(-3.29, 3.62)** | **0.77**  **(-3.59, 5.08)** | **0.04**  **(-4.27, 4.28)** | **0.49**  **(-1.97, 2.94)** | **0.01**  **(-4.29, 4.24)** | **BEV_TMZ** |  |  |  |  |  |  |  |  |  |
| **1.56**  **(-1.19, 4.47)** | **2.13**  **(-1.65, 5.98)** | **1.39**  **(-2.31, 5.15)** | **1.86**  **(-1.83, 5.67)** | **1.37**  **(-2.33, 5.16)** | **1.37**  **(-3.03, 5.88)** | **BEV5_CCNU90** |  |  |  |  |  |  |  |  |
| **2.33**  **(-0.31, 5.28)** | **2.93**  **(-0.8, 6.81)** | **2.15**  **(-0.52, 5.13)** | **2.62**  **(-0.93, 6.41)** | **2.14**  **(-1.46, 5.95)** | **2.14**  **(-2.18, 6.67)** | **0.78**  **(-3.13, 4.83)** | **CCNU** |  |  |  |  |  |  |  |
| **1.89**  **(-1.79, 5.71)** | **2.47**  **(-2.05, 7.07)** | **1.7**  **(-1.98, 5.62)** | **2.17**  **(-2.23, 6.67)** | **1.68**  **(-2.73, 6.26)** | **1.69**  **(-3.34, 6.85)** | **0.3**  **(-4.34, 5.07)** | **-0.45**  **(-2.99, 2.03)** | **CED** |  |  |  |  |  |  |
| **1.73**  **(-1.9, 5.52)** | **2.29**  **(-2.19, 6.91)** | **1.54**  **(-2.1, 5.39)** | **2.01**  **(-2.4, 6.54)** | **1.52**  **(-2.81, 6.08)** | **1.52**  **(-3.46, 6.69)** | **0.15**  **(-4.46, 4.91)** | **-0.63**  **(-3.15, 1.84)** | **-0.17**  **(-2.6, 2.27)** | **CED_CCNU** |  |  |  |  |  |
| **1.55**  **(-2.95, 6.13)** | **2.14**  **(-3.07, 7.32)** | **1.36**  **(-3.07, 5.94)** | **1.82**  **(-3.23, 6.97)** | **1.33**  **(-3.75, 6.48)** | **1.34**  **(-4.31, 7.04)** | **-0.04**  **(-5.32, 5.31)** | **-0.79**  **(-4.38, 2.71)** | **-0.35**  **(-2.85, 2.14)** | **-0.17**  **(-3.68, 3.29)** | **CED_GEF** |  |  |  |  |
| **2.98**  **(-0.77, 6.9)** | **3.58**  **(-1.00, 8.27)** | **2.8**  **(-1.02, 6.74)** | **3.25**  **(-1.22, 7.88)** | **2.79**  **(-1.72, 7.4)** | **2.77**  **(-2.34, 8)** | **1.41**  **(-3.37, 6.25)** | **0.64**  **(-2.01, 3.28)** | **1.1**  **(-2.55, 4.74)** | **1.28**  **(-2.42, 4.88)** | **1.44**  **(-2.96, 5.89)** | **ENZ** |  |  |  |
| **1.26**  **(-1.3, 3.94)** | **1.85**  **(-1.81, 5.56)** | **1.08**  **(-2.45, 4.69)** | **1.53**  **(-2.03, 5.14)** | **1.07**  **(-2.5, 4.72)** | **1.05**  **(-3.25, 5.44)** | **-0.29**  **(-4.19, 3.59)** | **-1.08**  **(-4.97, 2.66)** | **-0.61**  **(-5.26, 3.86)** | **-0.43**  **(-5.03, 4.04)** | **-0.27**  **(-5.52, 4.91)** | **-1.71**  **(-6.42, 2.87)** | **FOT** |  |  |
| **2.35**  **(-1.54, 6.39)** | **2.91**  **(-1.76, 7.74)** | **2.18**  **(-1.75, 6.23)** | **2.64**  **(-1.96, 7.38)** | **2.14**  **(-2.43, 6.91)** | **2.17**  **(-3.03, 7.47)** | **0.76**  **(-4, 5.7)** | **-0.01**  **(-2.87, 2.84)** | **0.46**  **(-3.31, 4.26)** | **0.64**  **(-3.19, 4.41)** | **0.82**  **(-3.71, 5.41)** | **-0.64**  **(-4.51, 3.3)** | **1.07**  **(-3.65, 5.94)** | **REG** |  |
| **-0.41**  **(-2.94, 2.08)** | **0.15**  **(-3.48, 3.79)** | **-0.6**  **(-4.12, 2.88)** | **-0.14**  **(-3.67, 3.31)** | **-0.62**  **(-4.19, 2.91)** | **-0.64**  **(-4.88, 3.68)** | **-2**  **(-5.81, 1.74)** | **-2.78**  **(-6.64, 0.87)** | **-2.33**  **(-6.92, 2.11)** | **-2.16**  **(-6.76, 2.25)** | **-1.98**  **(-7.2, 3.13)** | **-3.45**  **(-8.07, 1.1)** | **-1.71**  **(-5.4, 1.89)** | **-2.79**  **(-7.57, 1.85)** | **RIN_BEV** |

Table S 8 League table of head-to-head comparisons of all treatments on ORR

Data are RRs (95% CI) in the column-defining treatment compared with the row-defining treatment, and RRs higher than 1 favour the column-defining treatment.

4) SUCRA and cumulative probability plots


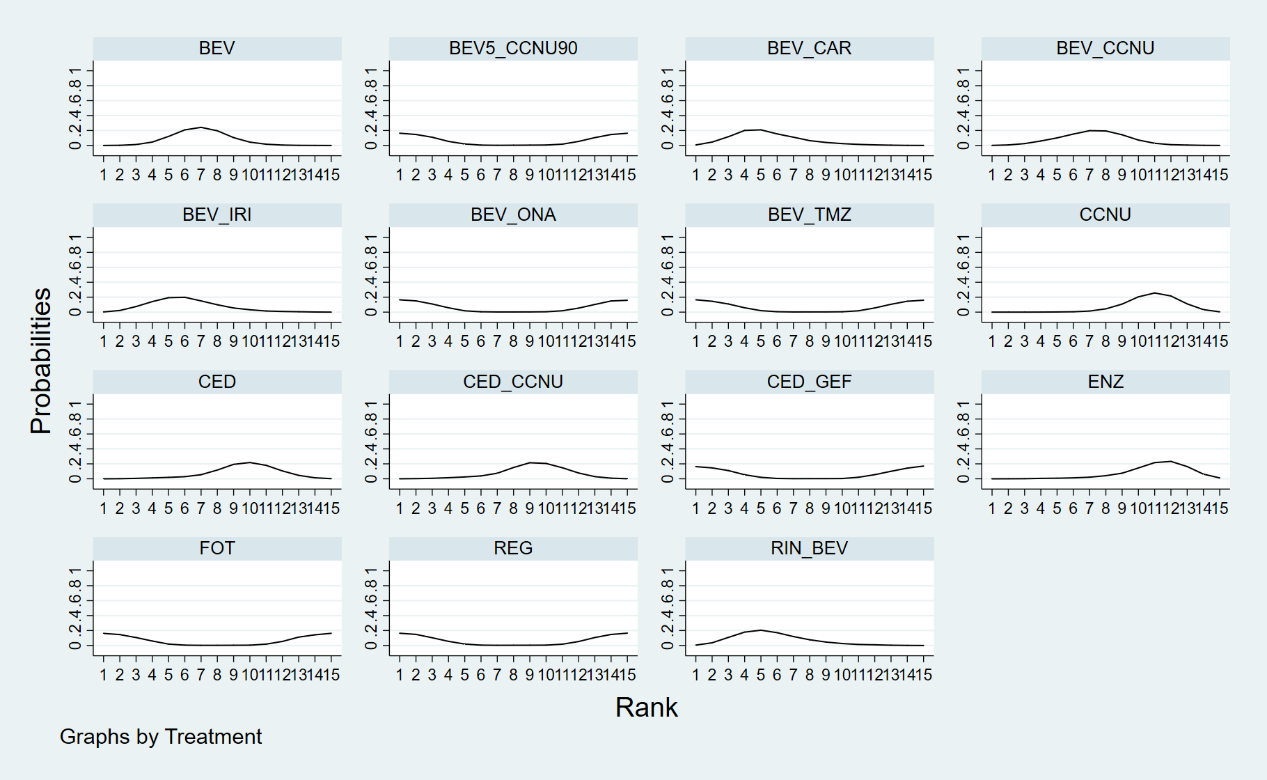


Figure S 3 Possibility of ranks for therapies involved in the model of ORR


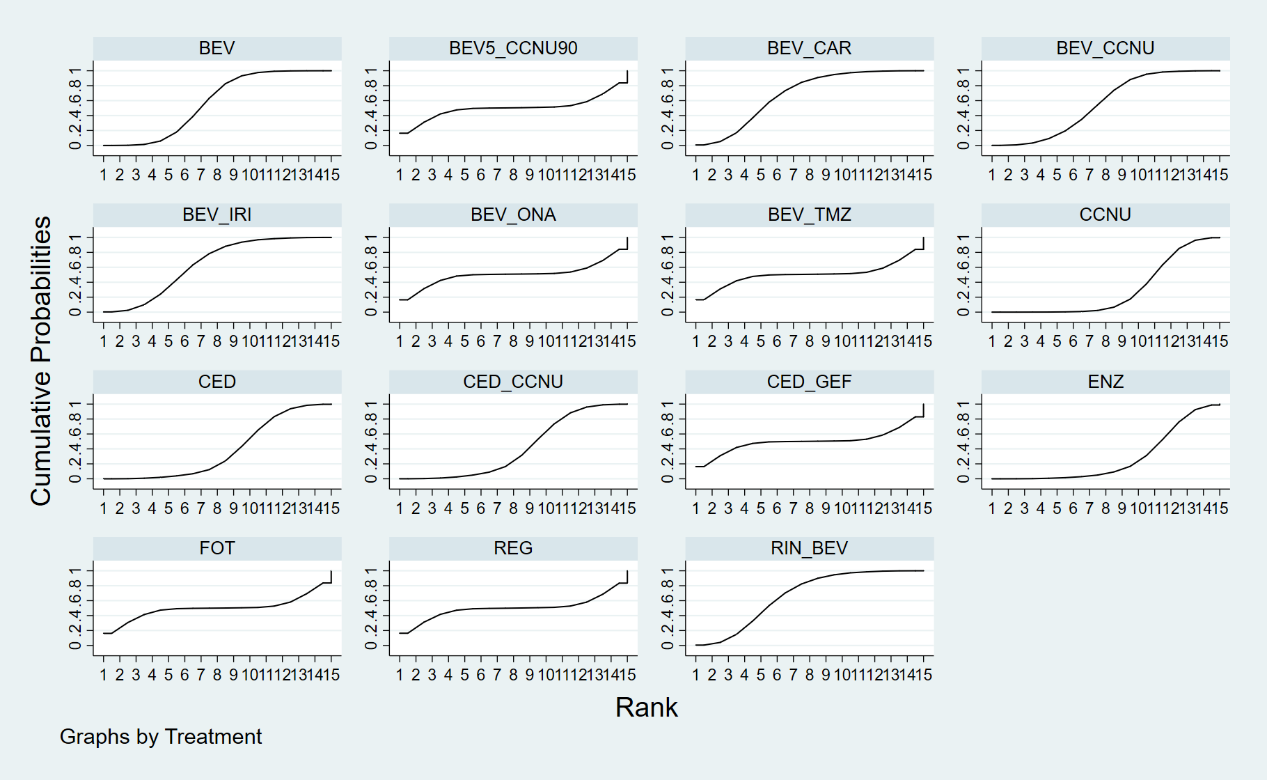


Figure S 4 Cumulative possibility of ranks for therapies involved in the model of ORR

| Treatm~t | \| | SUCRA | \| | PrBest | \| | MeanRank |
| --- | --- | --- | --- | --- | --- | --- |
| BV_CAR | \| | 85.7 | \| | 46.3 | \| | 2.7 |
| BV_IRI | \| | 81.4 | \| | 18.5 | \| | 3.2 |
| BV | \| | 71.5 | \| | 1.1 | \| | 4.4 |
| BV_TMZ | \| | 66.6 | \| | 9.3 | \| | 5 |
| BV_LOM90 | \| | 66.4 | \| | 6.9 | \| | 5 |
| BV_ONA | \| | 65.2 | \| | 6.7 | \| | 5.2 |
| CED_GEF | \| | 47.2 | \| | 7.1 | \| | 7.3 |
| FOT | \| | 38.5 | \| | 1.1 | \| | 8.4 |
| BV_L_LOM | \| | 35.7 | \| | 1.3 | \| | 8.7 |
| CED_LOM | \| | 35 | \| | 1.1 | \| | 8.8 |
| CED | \| | 29.2 | \| | 0.3 | \| | 9.5 |
| LOM | \| | 15.1 | \| | 0 | \| | 11.2 |
| ENZ | \| | 12.6 | \| | 0.3 | \| | 11.5 |

Table S 9 SUCRA of therapies involved in the model of ORR

Abbrevations: SUCRA, surface under the cumulative ranking curve; PrBest, possibility to be the

# 4. Supplementary results for 6m-progression free survival rate

1) Network plot


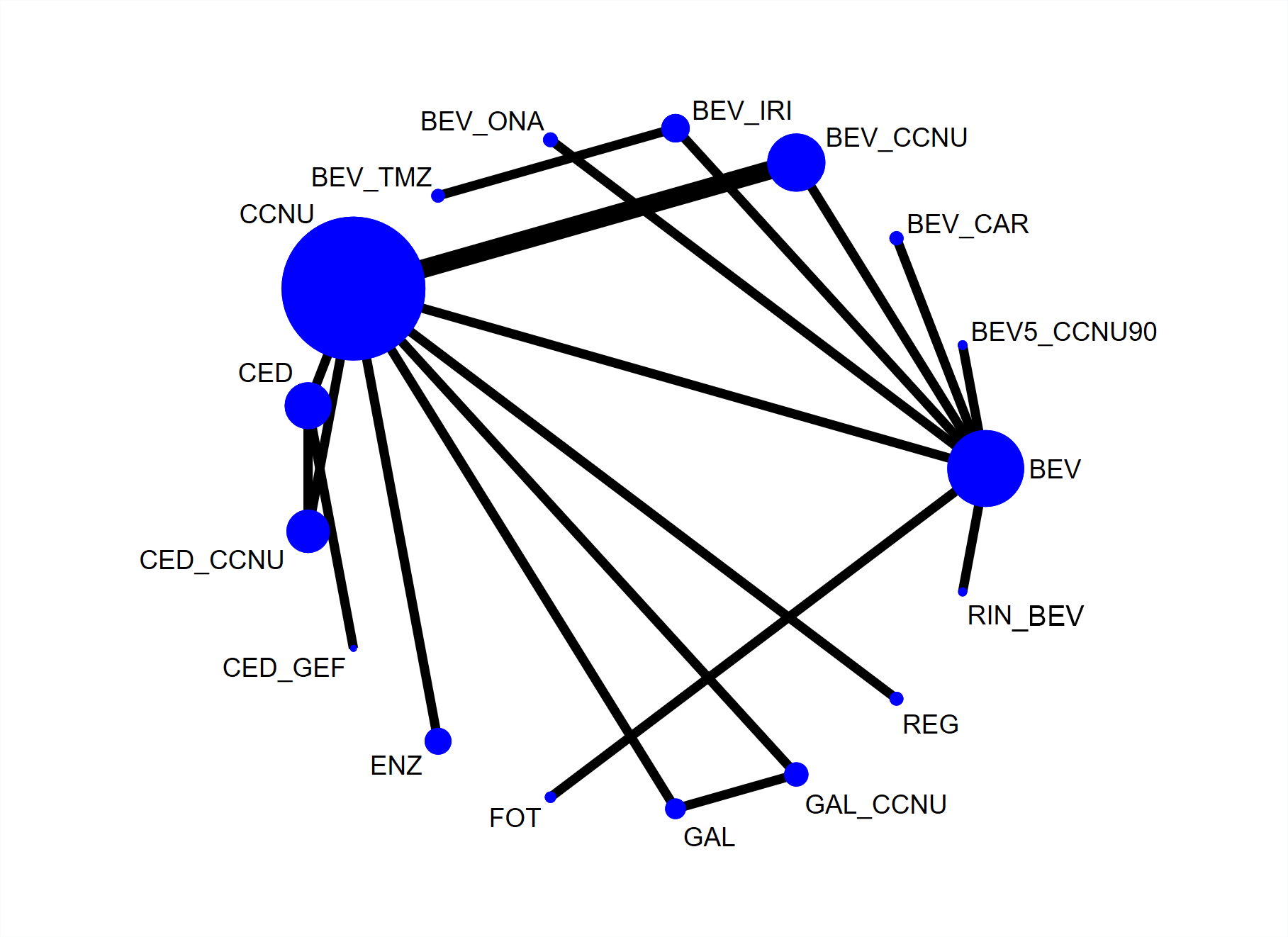


Figure S 5 Network plot of all comparisons involved in model of 6m-PFS rate

Size of every solid circle is proportional to the number of total sample size. TTF combined with BEV were omitted due to missing data.

2) Publication bias


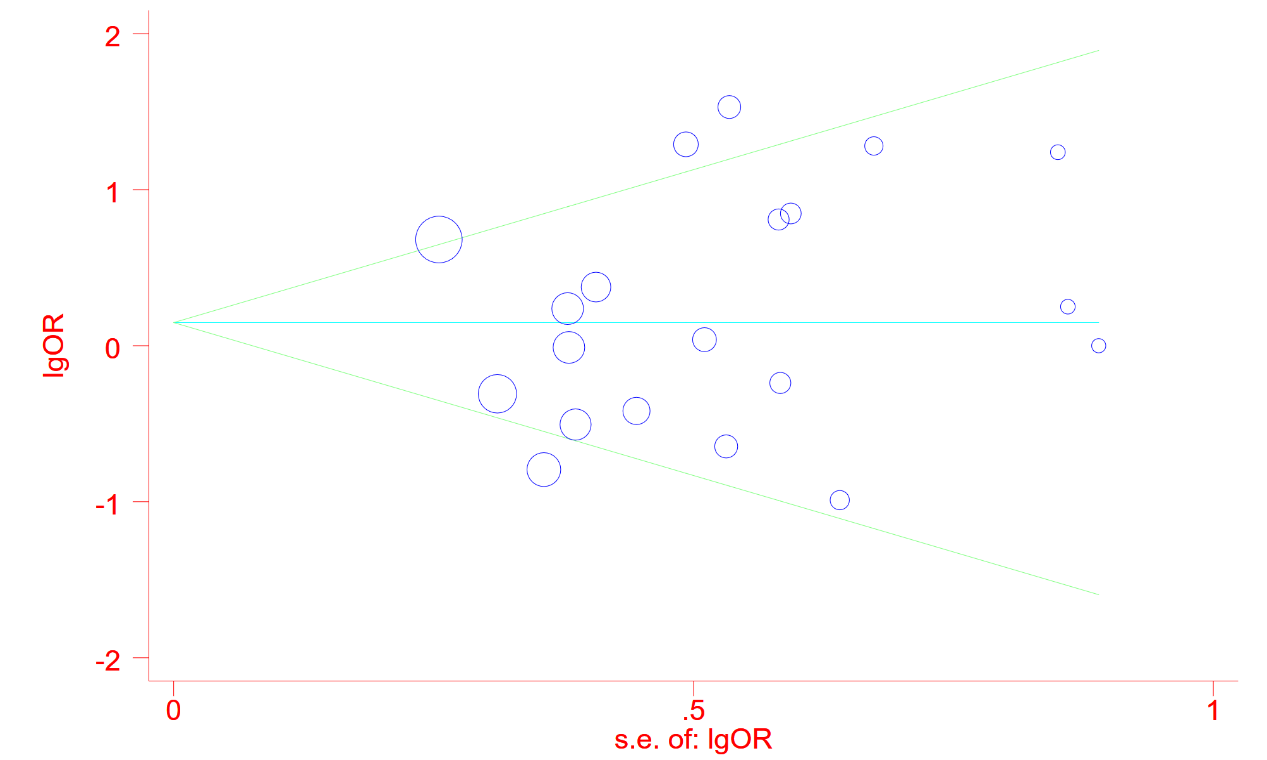


Begg's Test: Pr > |z| = 0.347

Egger's test: P > |t| = 0.540

3) Forest plot of relative effect compared with CCNU


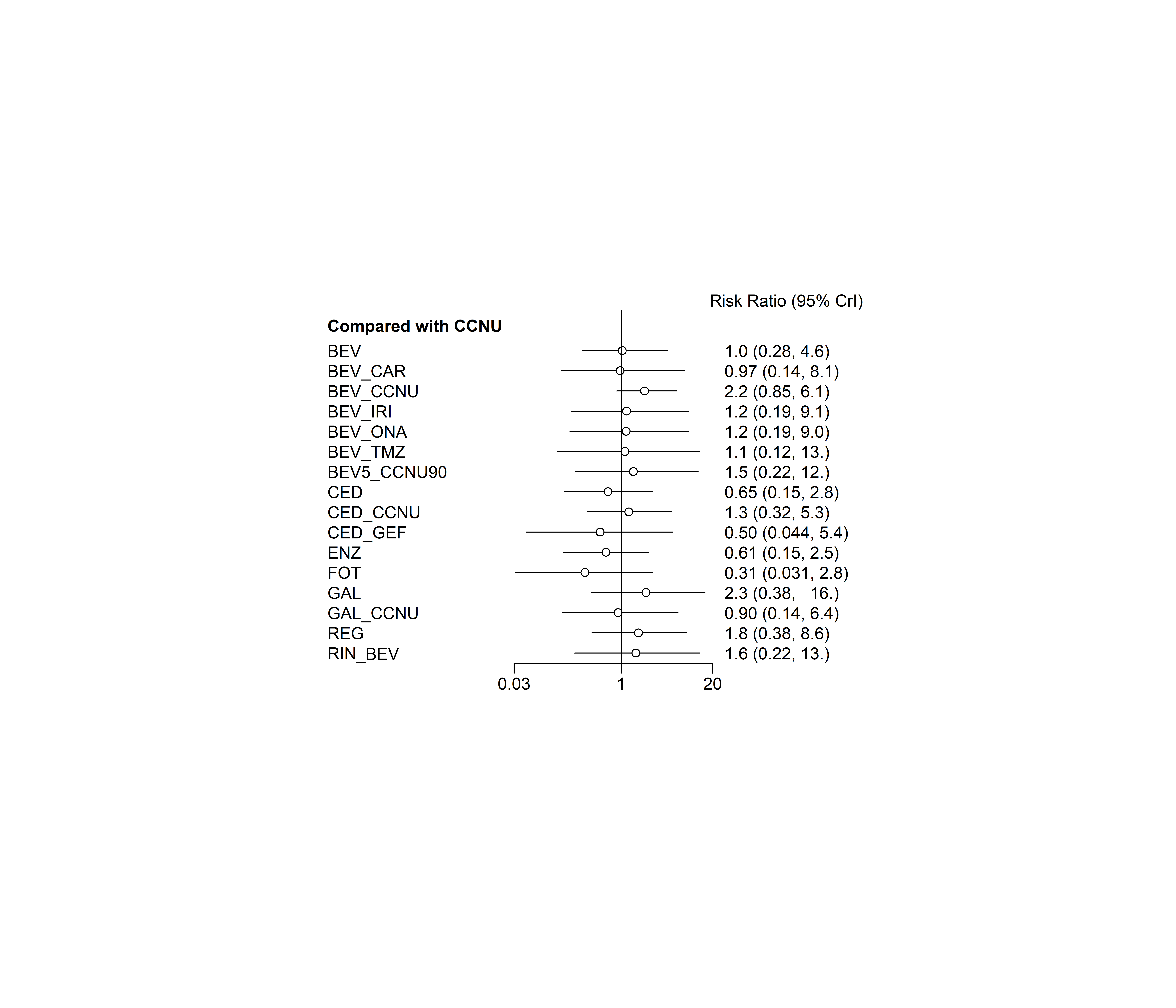


Figure S 6 Forest plot of relative effects compared with CCNU

All therapies were compared with CCNU about efficacy on 6m-PFS rate.

3) Head-to-head comparisons of all treatments

| **BEV** |  |  |  |  |  |  |  |  |  |  |  |  |  |  |  |  |
| --- | --- | --- | --- | --- | --- | --- | --- | --- | --- | --- | --- | --- | --- | --- | --- | --- |
| **0.08(-1.41, 1.55)** | **BEV_CAR** |  |  |  |  |  |  |  |  |  |  |  |  |  |  |  |
| **-0.73(-2.06, 0.6)** | **-0.83 (-2.78, 1.2)** | **BEV_CCNU** |  |  |  |  |  |  |  |  |  |  |  |  |  |  |
| **-0.14(-1.45, 1.16)** | **-0.23 (-2.18, 1.74)** | **0.59 (-1.3, 2.43)** | **BEV_IRI** |  |  |  |  |  |  |  |  |  |  |  |  |  |
| **-0.13(-1.47, 1.19)** | **-0.21 (-2.19, 1.79)** | **0.61 (-1.3, 2.46)** | **0.01 (-1.85, 1.87)** | **BEV_ONA** |  |  |  |  |  |  |  |  |  |  |  |  |
| **-0.1(-1.95, 1.73)** | **-0.19 (-2.55, 2.16)** | **0.63 (-1.67, 2.88)** | **0.04 (-1.28, 1.36)** | **0.03 (-2.24, 2.3)** | **BEV_TMZ** |  |  |  |  |  |  |  |  |  |  |  |
| **-0.39(-1.81, 1.04)** | **-0.46 (-2.52, 1.58)** | **0.36 (-1.61, 2.28)** | **-0.25 (-2.16, 1.67)** | **-0.26 (-2.18, 1.69)** | **-0.29 (-2.56, 2.05)** | **BEV5_CCNU90** |  |  |  |  |  |  |  |  |  |  |
| **0.04(-1.26, 1.5)** | **-0.05 (-1.98, 2.07)** | **0.76 (-0.13, 1.81)** | **0.18 (-1.63, 2.16)** | **0.16 (-1.64, 2.18)** | **0.14 (-2.06, 2.52)** | **0.41 (-1.48, 2.49)** | **CCNU** |  |  |  |  |  |  |  |  |  |
| **0.46(-1.46, 2.51)** | **0.37 (-2, 2.94)** | **1.19 (-0.46, 2.98)** | **0.6 (-1.69, 3.05)** | **0.58 (-1.71, 3.06)** | **0.55 (-2.04, 3.37)** | **0.83 (-1.5, 3.36)** | **0.42 (-0.99, 1.83)** | **CED** |  |  |  |  |  |  |  |  |
| **-0.23(-2.1, 1.79)** | **-0.31 (-2.65, 2.22)** | **0.5 (-1.11, 2.27)** | **-0.08 (-2.36, 2.35)** | **-0.09 (-2.35, 2.37)** | **-0.13 (-2.73, 2.64)** | **0.15 (-2.17, 2.65)** | **-0.26 (-1.65, 1.12)** | **-0.68 (-2.08, 0.69)** | **CED_CCNU** |  |  |  |  |  |  |  |
| **0.72(-1.94, 3.56)** | **0.63 (-2.41, 3.87)** | **1.44 (-1.01, 4.09)** | **0.86 (-2.08, 4.01)** | **0.83 (-2.1, 4.01)** | **0.81 (-2.39, 4.24)** | **1.09 (-1.91, 4.29)** | **0.67 (-1.64, 3.07)** | **0.24 (-1.6, 2.2)** | **0.93 (-1.35, 3.33)** | **CED_GEF** |  |  |  |  |  |  |
| **0.53(-1.32, 2.56)** | **0.45 (-1.88, 3)** | **1.27 (-0.35, 3.02)** | **0.68 (-1.57, 3.12)** | **0.67 (-1.57, 3.13)** | **0.64 (-1.95, 3.44)** | **0.91 (-1.41, 3.42)** | **0.5 (-0.89, 1.9)** | **0.07 (-1.89, 2.08)** | **0.76 (-1.2, 2.71)** | **-0.16 (-2.95, 2.51)** | **ENZ** |  |  |  |  |  |
| **1.18(-0.4, 3.02)** | **1.11 (-1.05, 3.46)** | **1.93 (-0.17, 4.16)** | **1.32 (-0.71, 3.59)** | **1.32 (-0.74, 3.59)** | **1.29 (-1.13, 3.9)** | **1.57 (-0.52, 3.88)** | **1.15 (-1.02, 3.37)** | **0.73 (-1.87, 3.38)** | **1.42 (-1.18, 4.04)** | **0.49 (-2.8, 3.71)** | **0.66 (-1.9, 3.26)** | **FOT** |  |  |  |  |
| **-0.8(-3.15, 1.5)** | **-0.88 (-3.64, 1.87)** | **-0.06 (-2.2, 2.02)** | **-0.66 (-3.32, 2)** | **-0.67 (-3.32, 2.02)** | **-0.71 (-3.63, 2.29)** | **-0.41 (-3.14, 2.29)** | **-0.83 (-2.82, 0.97)** | **-1.27 (-3.68, 1.02)** | **-0.59 (-2.97, 1.68)** | **-1.55 (-4.62, 1.45)** | **-1.34 (-3.76, 0.91)** | **-2.02 (-4.92, 0.83)** | **GAL** |  |  |  |
| **0.09(-2.27, 2.44)** | **0.01 (-2.75, 2.8)** | **0.84 (-1.37, 2.94)** | **0.23 (-2.43, 2.95)** | **0.2 (-2.43, 2.96)** | **0.19 (-2.74, 3.19)** | **0.48 (-2.27, 3.25)** | **0.07 (-1.97, 1.88)** | **-0.38 (-2.81, 1.94)** | **0.31 (-2.12, 2.6)** | **-0.65 (-3.77, 2.38)** | **-0.44 (-2.88, 1.84)** | **-1.14 (-4.04, 1.75)** | **0.9 (-0.76, 2.57)** | **GAL_CCNU** |  |  |
| **-0.54(-2.56, 1.62)** | **-0.62 (-3.13, 2.01)** | **0.21 (-1.6, 2.07)** | **-0.39 (-2.78, 2.12)** | **-0.41 (-2.81, 2.16)** | **-0.44 (-3.11, 2.41)** | **-0.15 (-2.61, 2.42)** | **-0.56 (-2.16, 0.96)** | **-0.99 (-3.1, 1.08)** | **-0.31 (-2.4, 1.74)** | **-1.24 (-4.12, 1.5)** | **-1.08 (-3.18, 0.97)** | **-1.74 (-4.45, 0.93)** | **0.25 (-2.1, 2.76)** | **-0.62 (-3.04, 1.89)** | **REG** |  |
| **-0.41(-1.9, 1.05)** | **-0.5 (-2.59, 1.6)** | **0.33 (-1.68, 2.28)** | **-0.27 (-2.26, 1.68)** | **-0.29 (-2.26, 1.71)** | **-0.3 (-2.67, 2.05)** | **-0.01 (-2.09, 2.02)** | **-0.45 (-2.56, 1.5)** | **-0.87 (-3.45, 1.52)** | **-0.18 (-2.75, 2.2)** | **-1.13 (-4.36, 1.9)** | **-0.95 (-3.48, 1.4)** | **-1.6 (-3.96, 0.54)** | **0.4 (-2.38, 3.13)** | **-0.49 (-3.3, 2.25)** | **0.13 (-2.51, 2.59)** | **RIN_BEV** |

Table S 10 League table of head-to-head comparisons of all treatments on 6m-PFS rate

Data are RRs (95% CI) in the column-defining treatment compared with the row-defining treatment, and RRs higher than 1 favour the column-defining treatment.

Abbrevations: RR, risk ratio.

4) SUCRA and cumulative probability plots


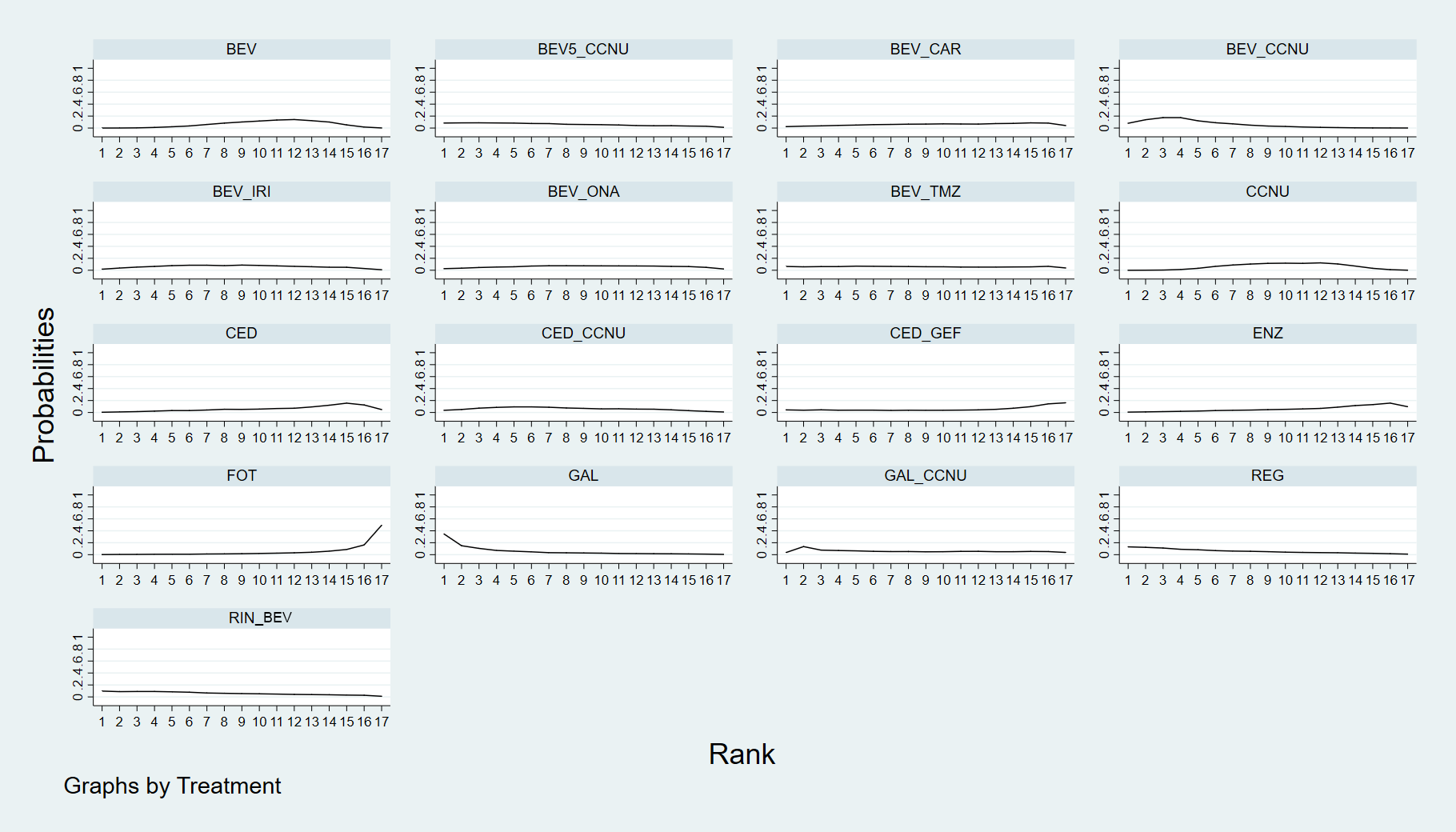


Figure S 7 Possibility of ranks for therapies involved in the model of 6m-PFS rate.


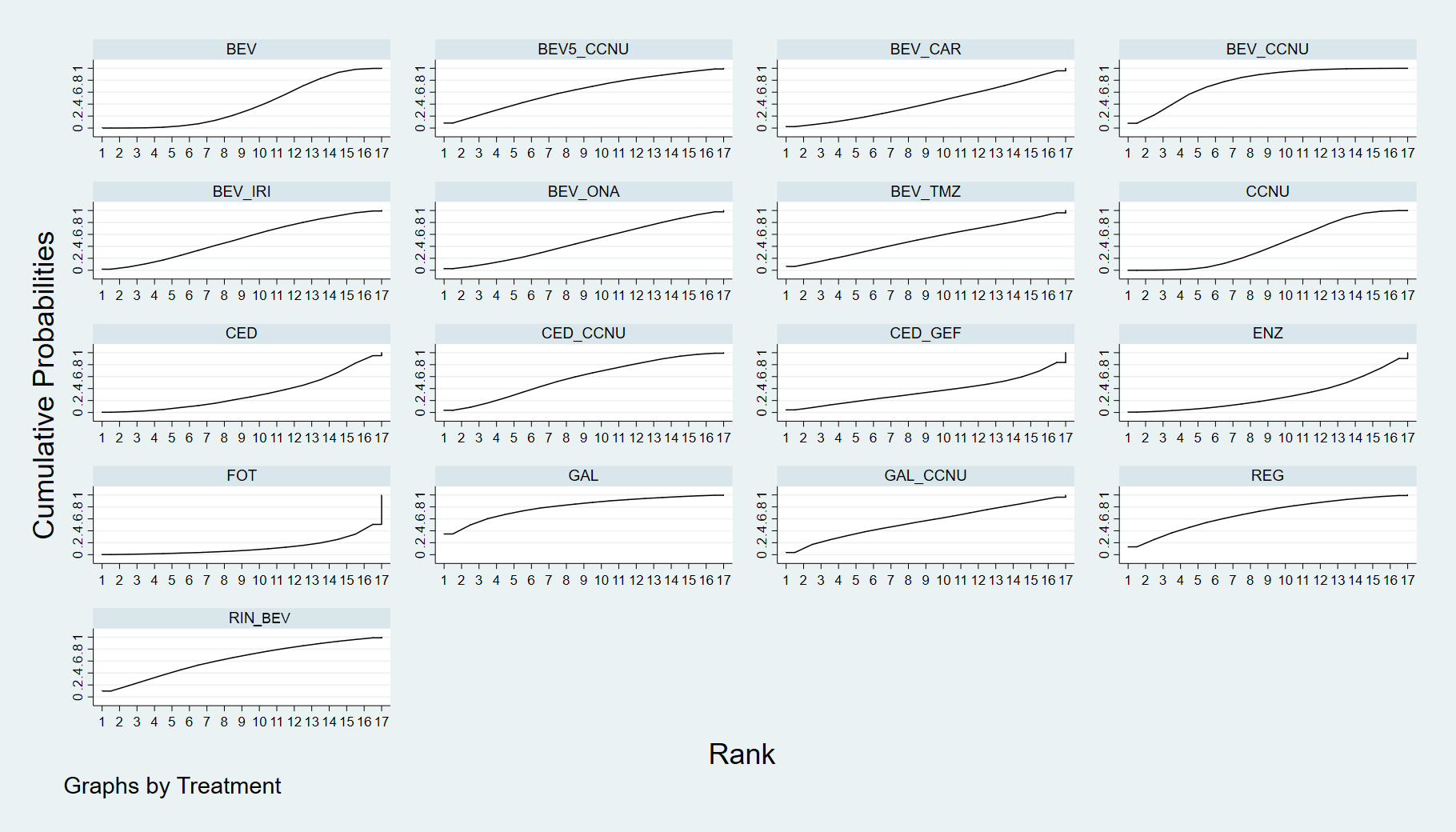


Figure S 8 Cumulative possibility of ranks for therapies involved in the model of 6m-PFS rate

| +---------------------------------------+ | | | | |  |  |  |  |
| --- | --- | --- | --- | --- | --- | --- | --- | --- |
| \| | Treatment | \| | SUCRA | \| | PrBest | \| | MeanRank | \| |
| \| | GAL | \| | 80.3 | \| | 34.7 | \| | 4.2 | \| |
| \| | BEV_CCNU | \| | 76.8 | \| | 8 | \| | 4.7 | \| |
| \| | REG | \| | 68.4 | \| | 13.1 | \| | 6.1 | \| |
| \| | RIN_BEV | \| | 63.1 | \| | 9.8 | \| | 6.9 | \| |
| \| | BEV5_CCNU | \| | 61.6 | \| | 8.4 | \| | 7.1 | \| |
| \| | CED_CCNU | \| | 57.7 | \| | 3.7 | \| | 7.8 | \| |
| \| | GAL_CCNU | \| | 55.4 | \| | 3.6 | \| | 8.1 | \| |
| \| | BEV_IRI | \| | 52.2 | \| | 1.8 | \| | 8.7 | \| |
| \| | BEV_TMZ | \| | 52 | \| | 6.3 | \| | 8.7 | \| |
| \| | BEV_ONA | \| | 48.4 | \| | 2.7 | \| | 9.3 | \| |
| \| | CCNU | \| | 43.3 | \| | 0 | \| | 10.1 | \| |
| \| | BEV_CAR | \| | 43 | \| | 2.4 | \| | 10.1 | \| |
| \| | BEV | \| | 38.9 | \| | 0 | \| | 10.8 | \| |
| \| | CED_GEF | \| | 35.9 | \| | 4.4 | \| | 11.2 | \| |
| \| | CED | \| | 31.7 | \| | 0.4 | \| | 11.9 | \| |
| \| | ENZ | \| | 29 | \| | 0.6 | \| | 12.4 | \| |
| \| | FOT | \| | 12.3 | \| | 0.2 | \| | 15 | \| |
| \|-----------+-------+--------+----------\| | | | | |  |  |  |  |
| +---------------------------------------+ | | | | |  |  |  |  |

Table S 11 SUCRA of therapies involved in the model of 6m-PFS rate

Abbrevations: SUCRA, surface under the cumulative ranking curve; PrBest, possibility to be the best.

# 5. Supplementary results for overall survival

1) Network plot


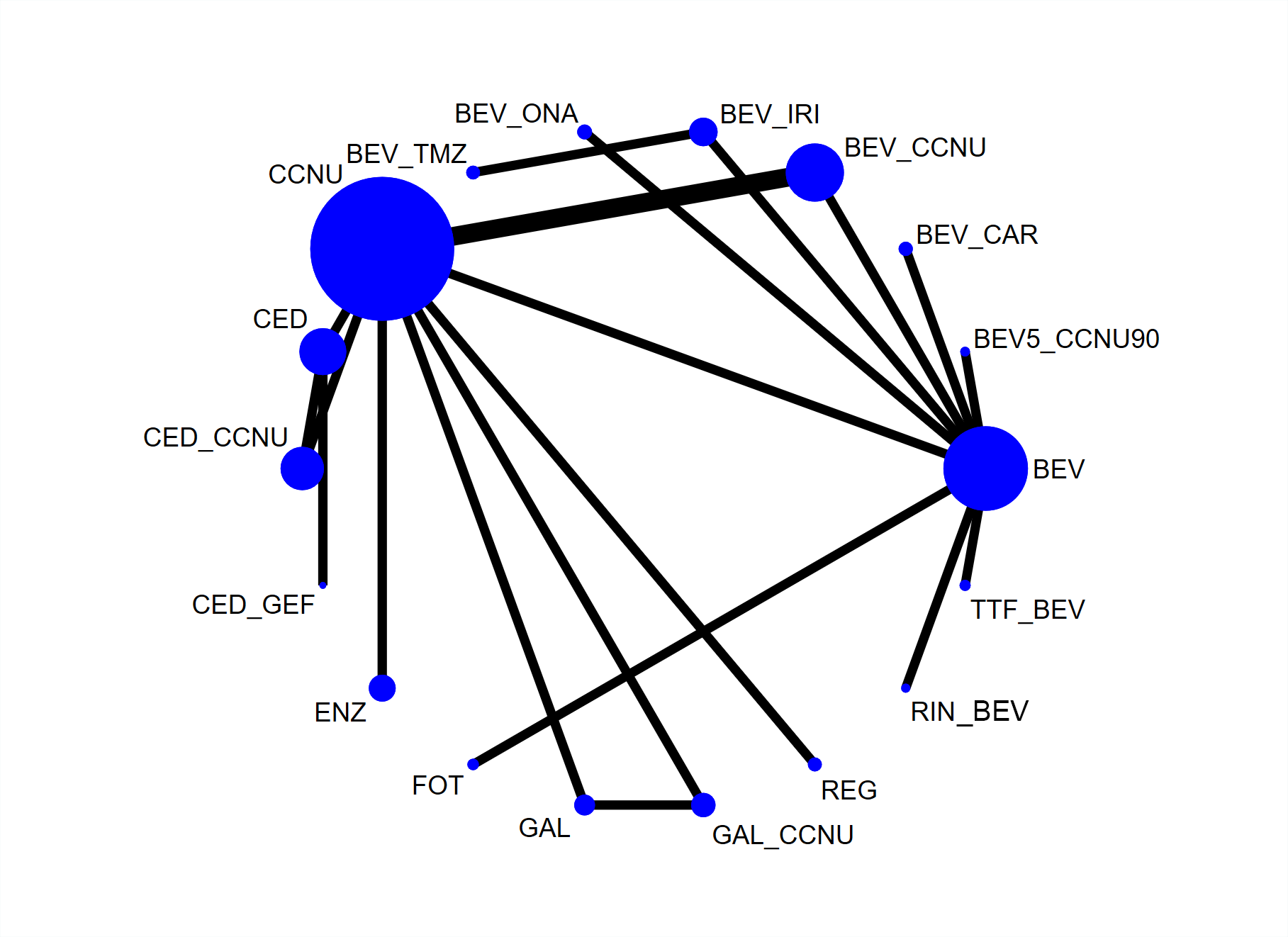


Figure S 9 Network plot of all comparisons involved in model of OS

Size of every solid circle is proportional to the number of total sample size.

2) Publication bias


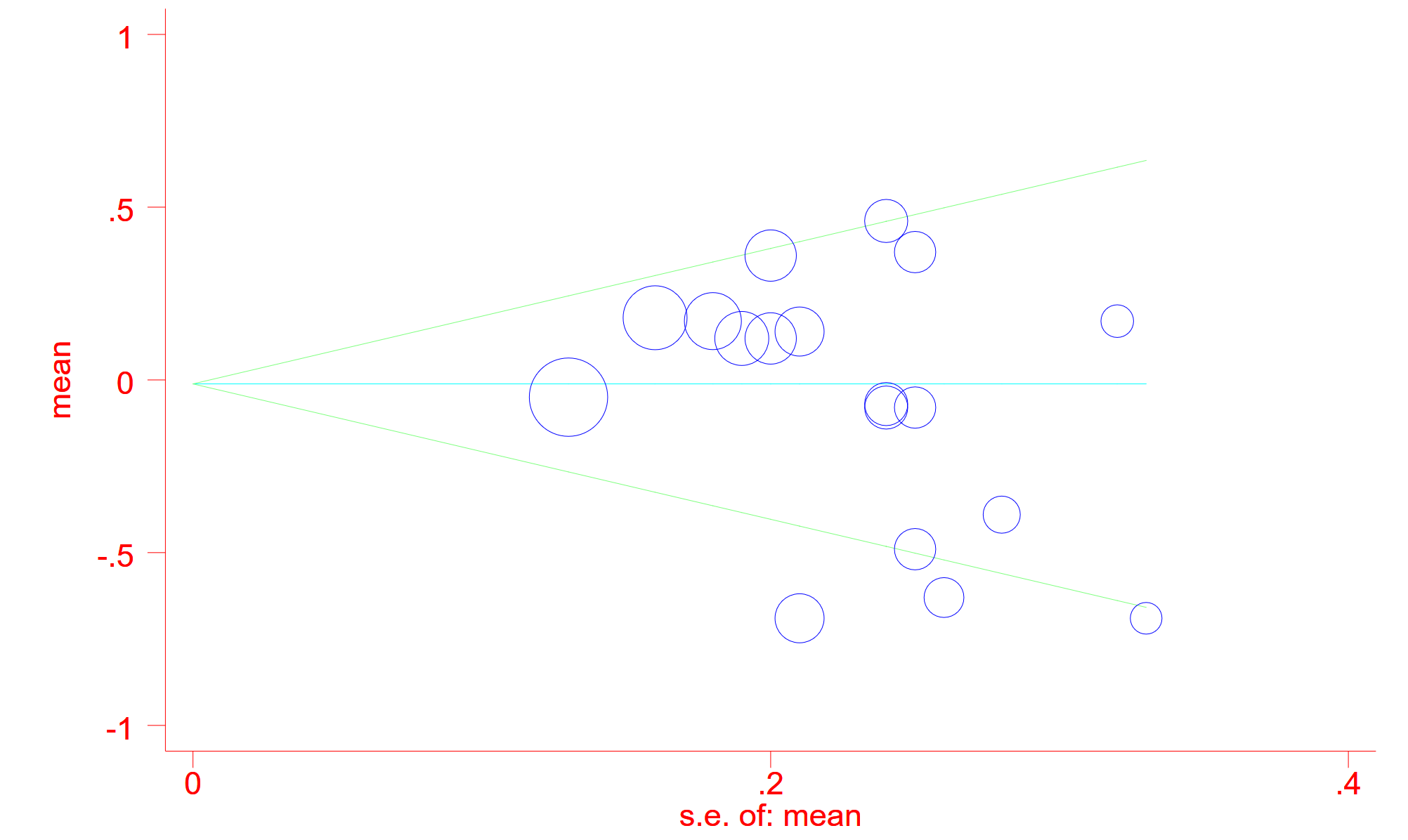


Begg's Test: Pr > |z| = 0.093

Egger's test: P > |t| = 0.240

3) Forest plot of relative effect compared with CCNU


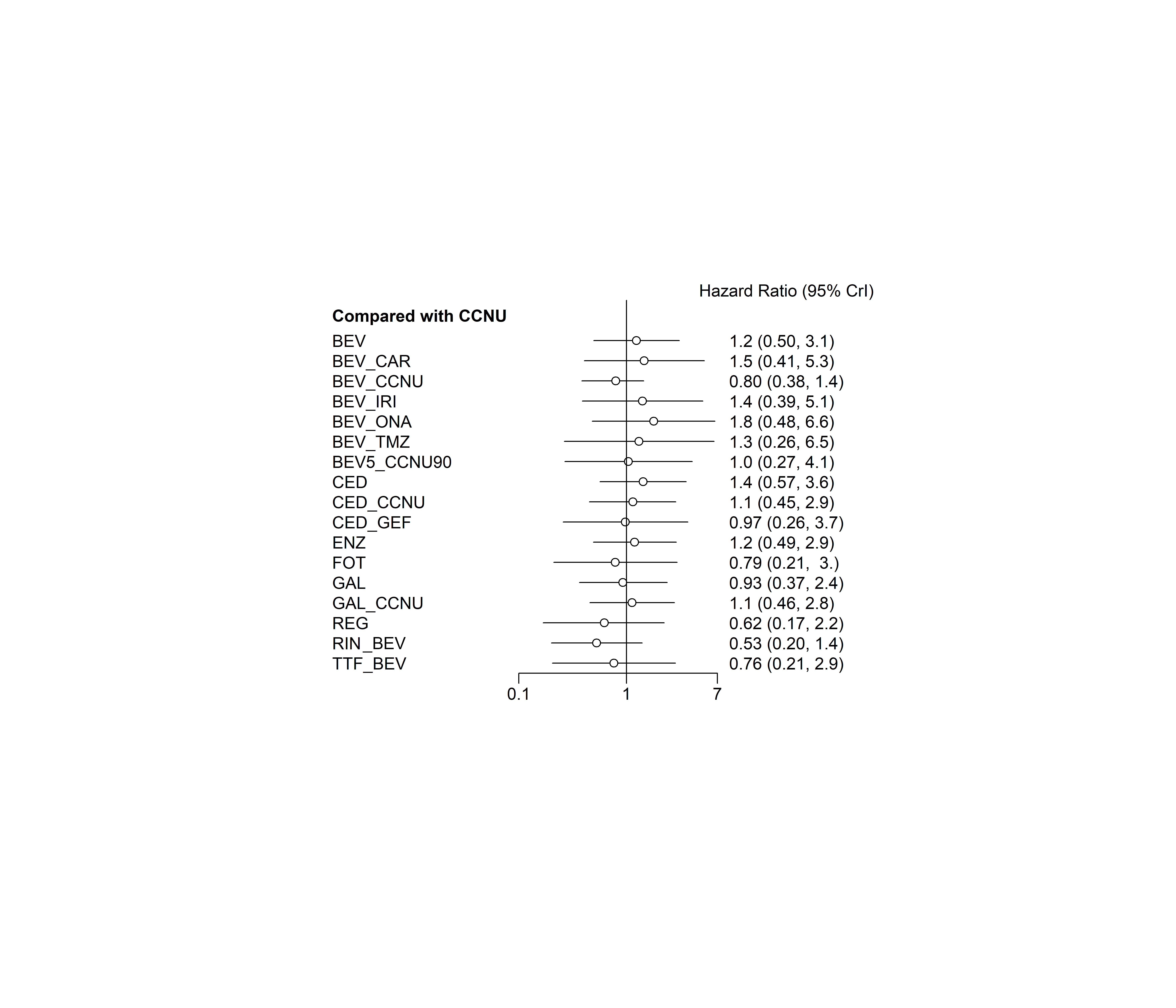


Figure S 10 Forest plot of relative effects compared with CCNU

All therapies were compared with CCNU about efficacy on OS.

3) Head-to-head comparisons of all treatments

| **BEV** |  |  |  |  |  |  |  |  |  |  |  |  |  |  |  |  |  |
| --- | --- | --- | --- | --- | --- | --- | --- | --- | --- | --- | --- | --- | --- | --- | --- | --- | --- |
| **-0.16 (-1.08, 0.74)** | **BEV_CAR** |  |  |  |  |  |  |  |  |  |  |  |  |  |  |  |  |
| **0.43 (-0.43, 1.44)** | **0.6 (-0.63, 1.98)** | **BEV_CCNU** |  |  |  |  |  |  |  |  |  |  |  |  |  |  |  |
| **-0.12 (-1.05, 0.81)** | **0.05 (-1.25, 1.33)** | **-0.55 (-1.94, 0.69)** | **BEV_IRI** |  |  |  |  |  |  |  |  |  |  |  |  |  |  |
| **-0.37 (-1.33, 0.59)** | **-0.2 (-1.52, 1.11)** | **-0.8 (-2.21, 0.47)** | **-0.25 (-1.57, 1.07)** | **BEV_ONA** |  |  |  |  |  |  |  |  |  |  |  |  |  |
| **-0.03 (-1.37, 1.3)** | **0.14 (-1.48, 1.73)** | **-0.46 (-2.18, 1.09)** | **0.09 (-0.87, 1.04)** | **0.34 (-1.29, 1.95)** | **BEV_TMZ** |  |  |  |  |  |  |  |  |  |  |  |  |
| **0.16 (-0.85, 1.19)** | **0.33 (-1.01, 1.7)** | **-0.27 (-1.72, 1.06)** | **0.29 (-1.08, 1.66)** | **0.53 (-0.85, 1.93)** | **0.2 (-1.46, 1.88)** | **BEV5_CCNU90** |  |  |  |  |  |  |  |  |  |  |  |
| **0.2 (-0.68, 1.11)** | **0.37 (-0.89, 1.66)** | **-0.22 (-0.95, 0.37)** | **0.32 (-0.95, 1.63)** | **0.57 (-0.72, 1.9)** | **0.23 (-1.37, 1.86)** | **0.04 (-1.32, 1.43)** | **CCNU** |  |  |  |  |  |  |  |  |  |  |
| **-0.16 (-1.43, 1.13)** | **0.01 (-1.56, 1.59)** | **-0.59 (-1.78, 0.46)** | **-0.04 (-1.61, 1.54)** | **0.21 (-1.38, 1.81)** | **-0.13 (-1.96, 1.72)** | **-0.33 (-1.97, 1.33)** | **-0.36 (-1.28, 0.54)** | **CED** |  |  |  |  |  |  |  |  |  |
| **0.06 (-1.22, 1.34)** | **0.23 (-1.35, 1.81)** | **-0.38 (-1.58, 0.69)** | **0.18 (-1.39, 1.77)** | **0.43 (-1.17, 2.05)** | **0.09 (-1.74, 1.95)** | **-0.11 (-1.76, 1.56)** | **-0.15 (-1.06, 0.78)** | **0.21 (-0.68, 1.11)** | **CED_CCNU** |  |  |  |  |  |  |  |  |
| **0.22 (-1.36, 1.85)** | **0.39 (-1.42, 2.27)** | **-0.2 (-1.76, 1.22)** | **0.34 (-1.47, 2.23)** | **0.6 (-1.26, 2.48)** | **0.25 (-1.8, 2.36)** | **0.06 (-1.84, 2)** | **0.03 (-1.3, 1.36)** | **0.39 (-0.59, 1.37)** | **0.18 (-1.15, 1.51)** | **CED_GEF** |  |  |  |  |  |  |  |
| **0.03 (-1.24, 1.3)** | **0.19 (-1.36, 1.77)** | **-0.4 (-1.58, 0.63)** | **0.15 (-1.42, 1.71)** | **0.4 (-1.21, 1.99)** | **0.05 (-1.78, 1.91)** | **-0.14 (-1.76, 1.51)** | **-0.18 (-1.07, 0.72)** | **0.18 (-1.09, 1.47)** | **-0.03 (-1.32, 1.24)** | **-0.21 (-1.8, 1.41)** | **ENZ** |  |  |  |  |  |  |
| **0.46 (-0.5, 1.4)** | **0.63 (-0.68, 1.93)** | **0.03 (-1.39, 1.29)** | **0.58 (-0.75, 1.89)** | **0.84 (-0.53, 2.17)** | **0.49 (-1.14, 2.12)** | **0.29 (-1.1, 1.68)** | **0.26 (-1.07, 1.55)** | **0.62 (-0.99, 2.2)** | **0.4 (-1.22, 1.99)** | **0.24 (-1.67, 2.08)** | **0.44 (-1.19, 2.01)** | **FOT** |  |  |  |  |  |
| **0.27 (-1.03, 1.59)** | **0.43 (-1.13, 2.03)** | **-0.17 (-1.38, 0.93)** | **0.38 (-1.2, 2.01)** | **0.64 (-0.98, 2.29)** | **0.3 (-1.54, 2.2)** | **0.1 (-1.54, 1.78)** | **0.07 (-0.87, 1.02)** | **0.43 (-0.89, 1.76)** | **0.21 (-1.1, 1.54)** | **0.04 (-1.6, 1.69)** | **0.24 (-1.06, 1.55)** | **-0.2 (-1.81, 1.45)** | **GAL** |  |  |  |  |
| **0.08 (-1.18, 1.37)** | **0.25 (-1.29, 1.84)** | **-0.35 (-1.53, 0.71)** | **0.2 (-1.35, 1.79)** | **0.45 (-1.13, 2.06)** | **0.11 (-1.71, 1.97)** | **-0.09 (-1.71, 1.56)** | **-0.12 (-1.02, 0.79)** | **0.24 (-1.04, 1.53)** | **0.03 (-1.27, 1.31)** | **-0.14 (-1.76, 1.47)** | **0.06 (-1.22, 1.34)** | **-0.38 (-1.97, 1.22)** | **-0.19 (-1.13, 0.75)** | **GAL_CCNU** |  |  |  |
| **0.69 (-0.24, 1.61)** | **0.86 (-0.45, 2.15)** | **0.26 (-1.14, 1.49)** | **0.8 (-0.49, 2.11)** | **1.05 (-0.27, 2.38)** | **0.71 (-0.88, 2.35)** | **0.52 (-0.87, 1.9)** | **0.49 (-0.82, 1.76)** | **0.85 (-0.74, 2.4)** | **0.64 (-0.95, 2.18)** | **0.47 (-1.41, 2.29)** | **0.67 (-0.92, 2.23)** | **0.23 (-1.08, 1.55)** | **0.42 (-1.2, 2)** | **0.61 (-0.98, 2.18)** | **REG** |  |  |
| **0.84 (-0.48, 2.17)** | **1.01 (-0.6, 2.62)** | **0.4 (-0.83, 1.5)** | **0.95 (-0.65, 2.58)** | **1.2 (-0.41, 2.84)** | **0.87 (-0.99, 2.75)** | **0.67 (-1, 2.36)** | **0.63 (-0.33, 1.6)** | **1 (-0.33, 2.32)** | **0.78 (-0.56, 2.1)** | **0.6 (-1.04, 2.25)** | **0.81 (-0.51, 2.11)** | **0.37 (-1.24, 2.05)** | **0.57 (-0.78, 1.9)** | **0.75 (-0.57, 2.06)** | **0.14 (-1.46, 1.78)** | **RIN_BEV** |  |
| **0.49 (-0.47, 1.44)** | **0.65 (-0.67, 1.97)** | **0.04 (-1.37, 1.33)** | **0.6 (-0.71, 1.93)** | **0.86 (-0.49, 2.21)** | **0.52 (-1.11, 2.16)** | **0.33 (-1.09, 1.73)** | **0.28 (-1.04, 1.57)** | **0.64 (-0.95, 2.22)** | **0.43 (-1.17, 2.03)** | **0.26 (-1.61, 2.1)** | **0.46 (-1.14, 2.04)** | **0.03 (-1.32, 1.38)** | **0.21 (-1.42, 1.82)** | **0.4 (-1.2, 1.99)** | **-0.2 (-1.54, 1.13)** | **-0.35 (-1.98, 1.27)** | **TTF_BEV** |

Table S 12 League table of head-to-head comparisons of all treatments on OS

Data are HRs (95% CI) in the column-defining treatment compared with the row-defining treatment, and HRs lower than 1 favour the column-defining treatment.

Abbrevations: HR, hazard ratio. Other abbrevations were shown in the legend of Figure 2.

4) SUCRA and cumulative probability plots


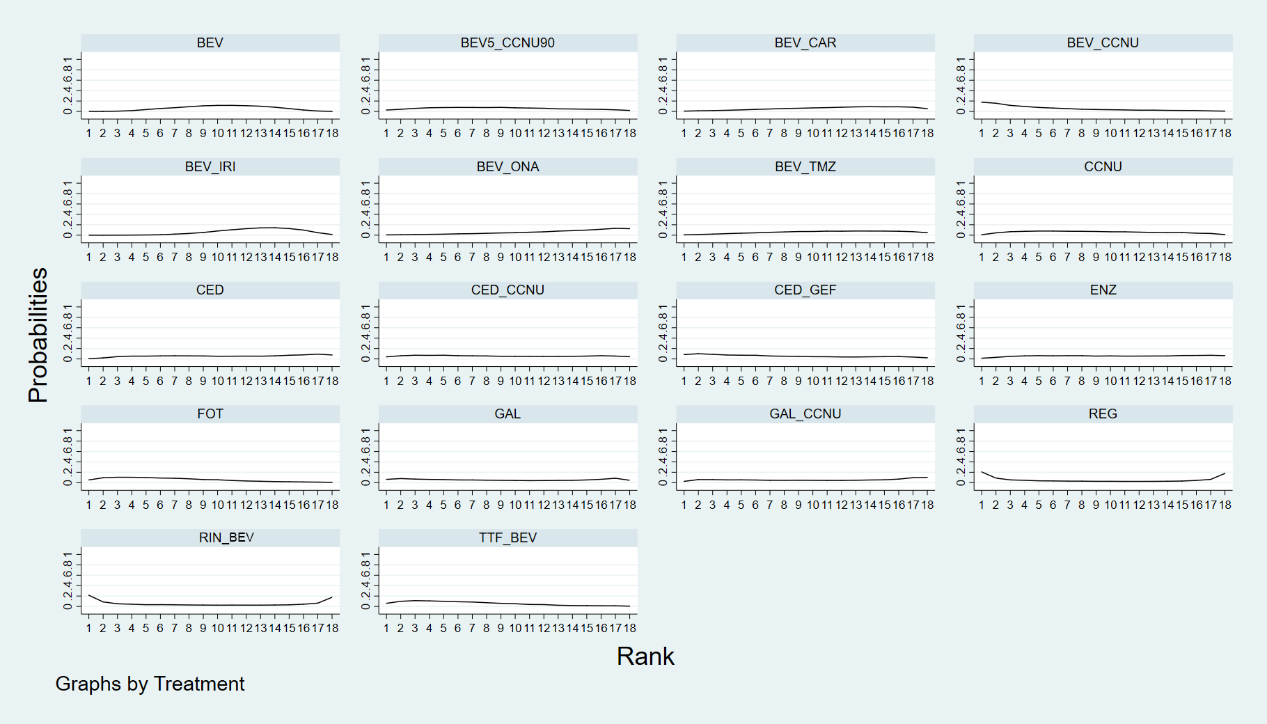


Figure S 11 Possibility of ranks for therapies involved in the model of OS

Abbrevations were shown in the legend of Figure 2.


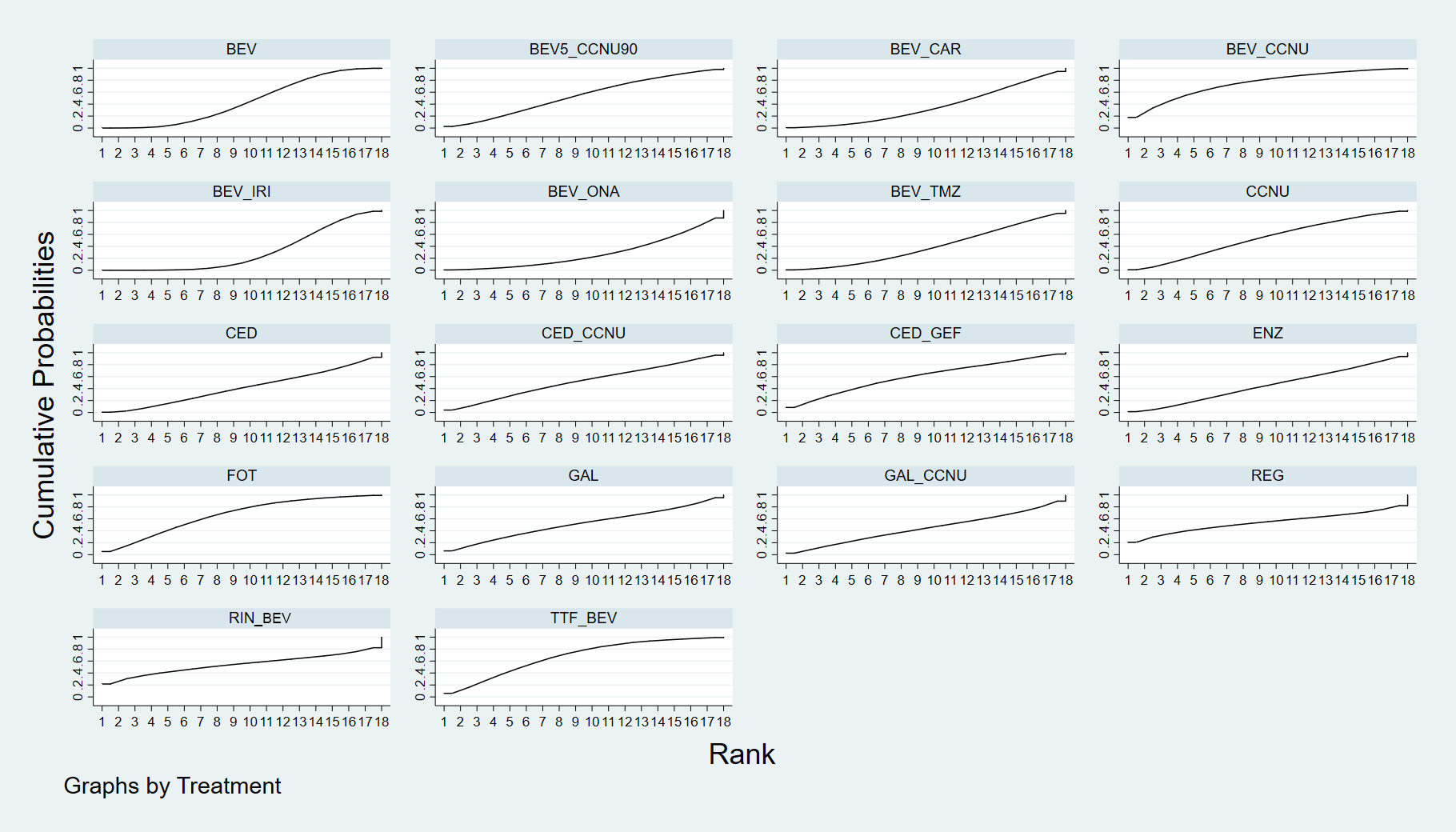


Figure S 12 Cumulative possibility of ranks for therapies involved in the model of OS

Abbrevations were shown in the legend of Figure 2.

| +-----------------------------------------+ | | | | |  |  |  |  |
| --- | --- | --- | --- | --- | --- | --- | --- | --- |
| \| | Treatment | \| | SUCRA | \| | PrBest | \| | MeanRank | \| |
| \| | BEV_CCNU | \| | 74.2 | \| | 17.7 | \| | 5.4 | \| |
| \| | TTF_BEV | \| | 67.9 | \| | 6 | \| | 6.5 | \| |
| \| | FOT | \| | 66.6 | \| | 5.3 | \| | 6.7 | \| |
| \| | CED_GEF | \| | 60.4 | \| | 8.4 | \| | 7.7 | \| |
| \| | BEV5_CCNU90 | \| | 54 | \| | 2.6 | \| | 8.8 | \| |
| \| | CCNU | \| | 53.9 | \| | 0.9 | \| | 8.8 | \| |
| \| | REG | \| | 53.9 | \| | 20.7 | \| | 8.8 | \| |
| \| | RIN_BEV | \| | 54.3 | \| | 21.6 | \| | 8.8 | \| |
| \| | CED_CCNU | \| | 52.1 | \| | 4.1 | \| | 9.1 | \| |
| \| | GAL | \| | 51.9 | \| | 6.4 | \| | 9.2 | \| |
| \| | ENZ | \| | 45.3 | \| | 1.4 | \| | 10.3 | \| |
| \| | BEV | \| | 44.6 | \| | 0 | \| | 10.4 | \| |
| \| | GAL_CCNU | \| | 44.3 | \| | 2.6 | \| | 10.5 | \| |
| \| | CED | \| | 41.5 | \| | 0.4 | \| | 10.9 | \| |
| \| | BEV_TMZ | \| | 39.6 | \| | 0.7 | \| | 11.3 | \| |
| \| | BEV_CAR | \| | 36.3 | \| | 0.6 | \| | 11.8 | \| |
| \| | BEV_IRI | \| | 30.7 | \| | 0 | \| | 12.8 | \| |
| \| | BEV_ONA | \| | 28.4 | \| | 0.6 | \| | 13.2 | \| |
| \|-------------+-------+--------+----------\| | | | | |  |  |  |  |
| +-----------------------------------------+ | | | | |  |  |  |  |

Table S 13 SUCRA of therapies involved in the model of OS

Abbrevations: SUCRA, surface under the cumulative ranking curve; PrBest, possibility to be the best. Other abbrevations were shown in the legend of Figure 2.

#

# 6. Sensitivity analysis

Because rindopepimut plus bevacizumab was researched in patients with EGFRvIII mutation, sensitivity analysis was done and aimed to excluding trials which involved patients with specific molecular biomarkers.

a. ORR

| BEV |  |  |  |  |  |  |  |  |  |  |  |  |  |
| --- | --- | --- | --- | --- | --- | --- | --- | --- | --- | --- | --- | --- | --- |
| -0.58 (-3.19, 2.03) | BEV_CAR |  |  |  |  |  |  |  |  |  |  |  |  |
| 0.17 (-2.27, 2.59) | 0.74 (-2.81, 4.31) | BEV_CCNU |  |  |  |  |  |  |  |  |  |  |  |
| -0.26 (-2.69, 2.16) | 0.33 (-3.24, 3.82) | -0.43 (-3.85, 3.01) | BEV_IRI |  |  |  |  |  |  |  |  |  |  |
| 0.2 (-2.26, 2.67) | 0.79 (-2.84, 4.38) | 0.04 (-3.46, 3.49) | 0.47 (-2.97, 3.91) | BEV_ONA |  |  |  |  |  |  |  |  |  |
| 0.22 (-3.23, 3.64) | 0.81 (-3.56, 5.06) | 0.03 (-4.14, 4.27) | 0.46 (-1.99, 2.93) | 0 (-4.24, 4.22) | BEV_TMZ |  |  |  |  |  |  |  |  |
| 1.53 (-1.18, 4.5) | 2.15 (-1.67, 6) | 1.37 (-2.25, 5.17) | 1.8 (-1.85, 5.6) | 1.35 (-2.35, 5.13) | 1.34 (-3.05, 5.82) | BEV5_CCNU90 |  |  |  |  |  |  |  |
| 2.34 (-0.34, 5.24) | 2.91 (-0.8, 6.8) | 2.18 (-0.51, 5.12) | 2.6 (-1.03, 6.36) | 2.13 (-1.48, 5.94) | 2.14 (-2.22, 6.58) | 0.77 (-3.17, 4.75) | CCNU |  |  |  |  |  |  |
| 1.87 (-1.75, 5.74) | 2.45 (-2.02, 7.1) | 1.72 (-1.93, 5.55) | 2.12 (-2.27, 6.68) | 1.65 (-2.69, 6.23) | 1.66 (-3.35, 6.79) | 0.28 (-4.38, 5.07) | -0.45 (-2.99, 2.07) | CED |  |  |  |  |  |
| 1.7 (-1.98, 5.53) | 2.27 (-2.22, 6.93) | 1.55 (-2.13, 5.39) | 1.97 (-2.46, 6.49) | 1.49 (-2.87, 6.04) | 1.51 (-3.5, 6.59) | 0.11 (-4.54, 4.88) | -0.62 (-3.16, 1.91) | -0.18 (-2.62, 2.31) | CED_CCNU |  |  |  |  |
| 1.51 (-2.89, 6.11) | 2.08 (-3.01, 7.39) | 1.36 (-3.09, 6) | 1.77 (-3.26, 6.95) | 1.29 (-3.69, 6.55) | 1.32 (-4.32, 7.01) | -0.08 (-5.32, 5.35) | -0.83 (-4.39, 2.78) | -0.36 (-2.88, 2.17) | -0.2 (-3.69, 3.33) | CED_GEF |  |  |  |
| 2.98 (-0.79, 6.89) | 3.58 (-1.01, 8.23) | 2.8 (-0.95, 6.77) | 3.25 (-1.22, 7.83) | 2.78 (-1.69, 7.36) | 2.76 (-2.28, 7.93) | 1.38 (-3.35, 6.21) | 0.65 (-2.03, 3.28) | 1.13 (-2.56, 4.71) | 1.29 (-2.36, 4.93) | 1.49 (-3.01, 5.91) | ENZ |  |  |
| 1.29 (-1.27, 3.99) | 1.87 (-1.79, 5.59) | 1.13 (-2.38, 4.77) | 1.55 (-2.01, 5.2) | 1.09 (-2.48, 4.75) | 1.09 (-3.25, 5.46) | -0.27 (-4.13, 3.58) | -1.05 (-4.92, 2.75) | -0.61 (-5.19, 3.96) | -0.41 (-5.02, 4.2) | -0.21 (-5.48, 4.96) | -1.71 (-6.39, 2.98) | FOT |  |
| 2.28 (-1.58, 6.33) | 2.83 (-1.79, 7.69) | 2.13 (-1.75, 6.21) | 2.55 (-1.97, 7.26) | 2.09 (-2.48, 6.78) | 2.08 (-3.1, 7.33) | 0.71 (-4.11, 5.59) | -0.05 (-2.91, 2.81) | 0.4 (-3.4, 4.24) | 0.58 (-3.21, 4.36) | 0.77 (-3.78, 5.35) | -0.69 (-4.51, 3.18) | 0.98 (-3.69, 5.78) | REG |

Table S 14 League table of head-to-head comparisons of all treatments on ORR, exclude trial of rindopepimut plus bevacizumab

b. 6m PFS rate

| BEV |  |  |  |  |  |  |  |  |  |  |  |  |  |  |  |
| --- | --- | --- | --- | --- | --- | --- | --- | --- | --- | --- | --- | --- | --- | --- | --- |
| 0.07 (-1.43, 1.59) | BEV_CAR |  |  |  |  |  |  |  |  |  |  |  |  |  |  |
| -0.74 (-2.09, 0.66) | -0.8 (-2.82, 1.25) | BEV_CCNU |  |  |  |  |  |  |  |  |  |  |  |  |  |
| -0.15 (-1.48, 1.17) | -0.22 (-2.23, 1.78) | 0.6 (-1.35, 2.46) | BEV_IRI |  |  |  |  |  |  |  |  |  |  |  |  |
| -0.13 (-1.51, 1.23) | -0.19 (-2.25, 1.84) | 0.62 (-1.38, 2.52) | 0.03 (-1.88, 1.93) | BEV_ONA |  |  |  |  |  |  |  |  |  |  |  |
| -0.13 (-2.01, 1.78) | -0.19 (-2.6, 2.22) | 0.62 (-1.73, 2.94) | 0.03 (-1.31, 1.4) | 0 (-2.31, 2.33) | BEV_TMZ |  |  |  |  |  |  |  |  |  |  |
| -0.39 (-1.84, 1.06) | -0.45 (-2.57, 1.63) | 0.37 (-1.69, 2.3) | -0.23 (-2.22, 1.74) | -0.26 (-2.25, 1.77) | -0.27 (-2.67, 2.13) | BEV5_CCNU90 |  |  |  |  |  |  |  |  |  |
| 0.03 (-1.31, 1.55) | -0.03 (-2.03, 2.12) | 0.76 (-0.16, 1.83) | 0.16 (-1.67, 2.25) | 0.16 (-1.7, 2.24) | 0.14 (-2.12, 2.61) | 0.4 (-1.51, 2.55) | CCNU |  |  |  |  |  |  |  |  |
| 0.47 (-1.46, 2.62) | 0.41 (-2.05, 3.02) | 1.21 (-0.48, 3.05) | 0.62 (-1.71, 3.16) | 0.58 (-1.74, 3.18) | 0.59 (-2.08, 3.47) | 0.84 (-1.55, 3.45) | 0.44 (-1.01, 1.88) | CED |  |  |  |  |  |  |  |
| -0.21 (-2.12, 1.92) | -0.28 (-2.69, 2.33) | 0.53 (-1.15, 2.33) | -0.06 (-2.38, 2.47) | -0.08 (-2.39, 2.45) | -0.1 (-2.78, 2.79) | 0.17 (-2.2, 2.76) | -0.25 (-1.66, 1.18) | -0.68 (-2.09, 0.73) | CED_CCNU |  |  |  |  |  |  |
| 0.75 (-1.92, 3.65) | 0.69 (-2.39, 3.96) | 1.5 (-0.99, 4.18) | 0.91 (-2.05, 4.11) | 0.88 (-2.11, 4.09) | 0.87 (-2.37, 4.34) | 1.13 (-1.89, 4.39) | 0.74 (-1.64, 3.15) | 0.29 (-1.56, 2.22) | 0.98 (-1.36, 3.38) | CED_GEF |  |  |  |  |  |
| 0.52 (-1.39, 2.61) | 0.46 (-1.94, 3.05) | 1.25 (-0.39, 3.05) | 0.67 (-1.61, 3.2) | 0.64 (-1.66, 3.17) | 0.65 (-2, 3.48) | 0.91 (-1.45, 3.46) | 0.5 (-0.92, 1.89) | 0.06 (-1.96, 2.1) | 0.73 (-1.28, 2.73) | -0.24 (-3.03, 2.52) | ENZ |  |  |  |  |
| 1.22 (-0.41, 3.06) | 1.16 (-1.09, 3.52) | 1.98 (-0.22, 4.25) | 1.38 (-0.72, 3.6) | 1.35 (-0.77, 3.63) | 1.36 (-1.15, 3.93) | 1.61 (-0.6, 3.95) | 1.2 (-1.07, 3.44) | 0.76 (-1.93, 3.39) | 1.46 (-1.24, 4.06) | 0.47 (-2.86, 3.69) | 0.71 (-1.99, 3.3) | FOT |  |  |  |
| -0.8 (-3.11, 1.61) | -0.87 (-3.59, 1.98) | -0.06 (-2.14, 2.05) | -0.65 (-3.26, 2.1) | -0.69 (-3.28, 2.16) | -0.69 (-3.58, 2.38) | -0.43 (-3.08, 2.42) | -0.83 (-2.73, 0.97) | -1.27 (-3.61, 1.04) | -0.59 (-2.91, 1.7) | -1.57 (-4.55, 1.43) | -1.33 (-3.66, 0.96) | -2.04 (-4.98, 0.92) | GAL |  |  |
| 0.1 (-2.27, 2.58) | 0.02 (-2.75, 2.91) | 0.85 (-1.3, 3.02) | 0.24 (-2.4, 3.07) | 0.23 (-2.47, 3.09) | 0.21 (-2.73, 3.36) | 0.47 (-2.24, 3.36) | 0.07 (-1.89, 1.97) | -0.37 (-2.76, 2.04) | 0.32 (-2.06, 2.69) | -0.67 (-3.71, 2.4) | -0.42 (-2.84, 1.92) | -1.12 (-4.09, 1.87) | 0.9 (-0.8, 2.64) | GAL_CCNU |  |
| -0.57 (-2.62, 1.65) | -0.63 (-3.16, 2.06) | 0.18 (-1.64, 2.11) | -0.41 (-2.84, 2.2) | -0.44 (-2.88, 2.18) | -0.45 (-3.22, 2.49) | -0.18 (-2.67, 2.48) | -0.59 (-2.21, 0.99) | -1.03 (-3.19, 1.12) | -0.34 (-2.47, 1.78) | -1.33 (-4.2, 1.53) | -1.09 (-3.21, 1.03) | -1.8 (-4.5, 0.96) | 0.23 (-2.17, 2.67) | -0.65 (-3.14, 1.81) | REG |

Table S 15 League table of head-to-head comparisons of all treatments on 6m PFS rate, exclude trial of rindopepimut plus bevacizumab

c. OS

| BEV |  |  |  |  |  |  |  |  |  |  |  |  |  |  |  |  |
| --- | --- | --- | --- | --- | --- | --- | --- | --- | --- | --- | --- | --- | --- | --- | --- | --- |
| -0.17 (-1.09, 0.76) | BEV_CAR |  |  |  |  |  |  |  |  |  |  |  |  |  |  |  |
| 0.45 (-0.46, 1.44) | 0.61 (-0.66, 1.97) | BEV_CCNU |  |  |  |  |  |  |  |  |  |  |  |  |  |  |
| -0.11 (-1.06, 0.81) | 0.05 (-1.27, 1.36) | -0.56 (-1.95, 0.72) | BEV_IRI |  |  |  |  |  |  |  |  |  |  |  |  |  |
| -0.36 (-1.34, 0.6) | -0.19 (-1.55, 1.12) | -0.81 (-2.22, 0.49) | -0.25 (-1.6, 1.09) | BEV_ONA |  |  |  |  |  |  |  |  |  |  |  |  |
| -0.04 (-1.39, 1.31) | 0.13 (-1.52, 1.74) | -0.48 (-2.17, 1.11) | 0.08 (-0.89, 1.05) | 0.32 (-1.32, 2) | BEV_TMZ |  |  |  |  |  |  |  |  |  |  |  |
| 0.17 (-0.87, 1.22) | 0.33 (-1.07, 1.72) | -0.28 (-1.73, 1.1) | 0.28 (-1.1, 1.7) | 0.53 (-0.89, 1.96) | 0.2 (-1.5, 1.9) | BEV5_CCNU90 |  |  |  |  |  |  |  |  |  |  |
| 0.2 (-0.72, 1.11) | 0.37 (-0.94, 1.67) | -0.24 (-0.98, 0.38) | 0.32 (-0.99, 1.64) | 0.56 (-0.78, 1.91) | 0.24 (-1.4, 1.87) | 0.03 (-1.37, 1.42) | CCNU |  |  |  |  |  |  |  |  |  |
| -0.16 (-1.48, 1.14) | 0.01 (-1.6, 1.59) | -0.6 (-1.82, 0.48) | -0.05 (-1.66, 1.57) | 0.2 (-1.43, 1.84) | -0.11 (-2.02, 1.74) | -0.33 (-2, 1.34) | -0.36 (-1.3, 0.57) | CED |  |  |  |  |  |  |  |  |
| 0.07 (-1.25, 1.36) | 0.23 (-1.39, 1.82) | -0.38 (-1.6, 0.71) | 0.18 (-1.43, 1.79) | 0.42 (-1.21, 2.06) | 0.11 (-1.8, 1.97) | -0.11 (-1.79, 1.55) | -0.14 (-1.08, 0.8) | 0.22 (-0.67, 1.13) | CED_CCNU |  |  |  |  |  |  |  |
| 0.23 (-1.43, 1.86) | 0.4 (-1.5, 2.26) | -0.22 (-1.8, 1.26) | 0.34 (-1.54, 2.25) | 0.59 (-1.33, 2.5) | 0.27 (-1.85, 2.39) | 0.06 (-1.91, 2) | 0.03 (-1.35, 1.4) | 0.39 (-0.61, 1.38) | 0.17 (-1.18, 1.52) | CED_GEF |  |  |  |  |  |  |
| 0.02 (-1.27, 1.31) | 0.18 (-1.42, 1.79) | -0.42 (-1.61, 0.66) | 0.13 (-1.45, 1.73) | 0.38 (-1.21, 1.99) | 0.05 (-1.81, 1.93) | -0.16 (-1.81, 1.51) | -0.19 (-1.09, 0.73) | 0.17 (-1.13, 1.49) | -0.05 (-1.35, 1.27) | -0.21 (-1.86, 1.44) | ENZ |  |  |  |  |  |
| 0.46 (-0.5, 1.43) | 0.63 (-0.72, 1.95) | 0.01 (-1.38, 1.32) | 0.57 (-0.77, 1.94) | 0.82 (-0.55, 2.21) | 0.5 (-1.17, 2.16) | 0.29 (-1.16, 1.71) | 0.26 (-1.07, 1.6) | 0.62 (-0.99, 2.28) | 0.4 (-1.22, 2.05) | 0.23 (-1.67, 2.16) | 0.44 (-1.16, 2.07) | FOT |  |  |  |  |
| 0.27 (-1.08, 1.61) | 0.43 (-1.2, 2.05) | -0.17 (-1.4, 0.96) | 0.38 (-1.24, 2.02) | 0.63 (-1.01, 2.29) | 0.31 (-1.59, 2.2) | 0.1 (-1.6, 1.78) | 0.07 (-0.9, 1.04) | 0.42 (-0.91, 1.78) | 0.21 (-1.12, 1.56) | 0.03 (-1.64, 1.72) | 0.25 (-1.08, 1.57) | -0.2 (-1.84, 1.45) | GAL |  |  |  |
| 0.08 (-1.22, 1.39) | 0.25 (-1.36, 1.85) | -0.36 (-1.56, 0.75) | 0.2 (-1.41, 1.82) | 0.44 (-1.17, 2.1) | 0.12 (-1.75, 2) | -0.09 (-1.77, 1.59) | -0.13 (-1.05, 0.81) | 0.24 (-1.08, 1.58) | 0.02 (-1.3, 1.36) | -0.15 (-1.81, 1.52) | 0.06 (-1.25, 1.36) | -0.37 (-2.01, 1.25) | -0.19 (-1.15, 0.77) | GAL_CCNU |  |  |
| 0.69 (-0.25, 1.63) | 0.86 (-0.46, 2.16) | 0.25 (-1.14, 1.53) | 0.81 (-0.53, 2.14) | 1.05 (-0.3, 2.4) | 0.74 (-0.93, 2.37) | 0.52 (-0.88, 1.91) | 0.48 (-0.82, 1.81) | 0.85 (-0.76, 2.47) | 0.63 (-0.98, 2.26) | 0.46 (-1.43, 2.37) | 0.67 (-0.93, 2.27) | 0.23 (-1.12, 1.57) | 0.42 (-1.23, 2.06) | 0.61 (-1.01, 2.23) | REG |  |
| 0.49 (-0.49, 1.46) | 0.65 (-0.68, 1.98) | 0.04 (-1.38, 1.35) | 0.61 (-0.75, 1.95) | 0.84 (-0.52, 2.23) | 0.53 (-1.14, 2.18) | 0.32 (-1.12, 1.75) | 0.29 (-1.06, 1.62) | 0.64 (-0.99, 2.27) | 0.43 (-1.22, 2.05) | 0.25 (-1.65, 2.17) | 0.47 (-1.15, 2.09) | 0.02 (-1.35, 1.4) | 0.22 (-1.44, 1.88) | 0.41 (-1.23, 2.02) | -0.21 (-1.54, 1.17) | TTF_BEV |

Table S 16 League table of head-to-head comparisons of all treatments on OS, exclude trial of rindopepimut plus bevacizumab

# 7. Changes to the protocol

1) We used 6-month PFS rate instead of PFS as a primary outcome, since the former could be analyzed with farther limited missing data and was a representative symbol for local control.

2) We did descriptive statistics for adverse effects rather than quantificational analysis of the tolerance of therapies, because of distinctive principles in the assessment of adverse effects among RCTs.
